# Hierarchical joint analysis of marginal summary statistics—Part I: Multipopulation fine mapping and credible set construction

**DOI:** 10.1002/gepi.22562

**Published:** 2024-04-12

**Authors:** Jiayi Shen, Lai Jiang, Kan Wang, Anqi Wang, Fei Chen, Paul J. Newcombe, Christopher A. Haiman, David V. Conti

**Affiliations:** 1 Department of Population and Public Health Sciences, Division of Biostatistics, Keck School of Medicine, University of Southern California, Los Angeles, California, USA; 2 Department of Population and Public Health Science, Center for Genetic Epidemiology, Keck School of Medicine, University of Southern California, Los Angeles, California, USA; 3 MRC Biostatistics Unit, University of Cambridge, Cambridge, UK; 4 Norris Comprehensive Cancer Center, Keck School of Medicine, University of Southern California, Los Angeles, California, USA

**Keywords:** diverse populations, fine-mapping, GWAS, summary statistics

## Abstract

Recent advancement in genome-wide association studies (GWAS) comes from not only increasingly larger sample sizes but also the shift in focus towards underrepresented populations. Multipopulation GWAS increase power to detect novel risk variants and improve fine-mapping resolution by leveraging evidence and differences in linkage disequilibrium (LD) from diverse populations. Here, we expand upon our previous approach for single-population fine-mapping through Joint Analysis of Marginal SNP Effects (JAM) to a multipopulation analysis (mJAM). Under the assumption that true causal variants are common across studies, we implement a hierarchical model framework that conditions on multiple SNPs while explicitly incorporating the different LD structures across populations. The mJAM framework can be used to first select index variants using the mJAM likelihood with different feature selection approaches. In addition, we present a novel approach leveraging the ideas of mediation to construct credible sets for these index variants. Construction of such credible sets can be performed given any existing index variants. We illustrate the implementation of the mJAM likelihood through two implementations: mJAM-SuSiE (a Bayesian approach) and mJAM-Forward selection. Through simulation studies based on realistic effect sizes and levels of LD, we demonstrated that mJAM performs well for constructing concise credible sets that include the underlying causal variants. In real data examples taken from the most recent multipopulation prostate cancer GWAS, we showed several practical advantages of mJAM over other existing multipopulation methods.

## 1 | INTRODUCTION

The development of high-throughput genotyping and genotype imputation has boosted the application of genome-wide association studies (GWAS) which is now a standard approach to identify susceptibility loci or genomic regions for many complex diseases and traits (Buniello et al., 2019; Schaid et al., 2018). However, the linkage disequilibrium (LD) of single-nucleotide polymorphisms (SNPs) makes it challenging to determine the true causal variant(s) within a region or to further prioritize genetic variants for functional studies (Schaid et al., 2018; Visscher et al., 2012). Fine-mapping is a post-GWAS approach which seeks to specify the underlying causal variant and quantify the strength of effect.

More recent and advanced fine-mapping approaches attempt to jointly or conditionally analyze all SNPs within a region, and include stepwise regression (Schaid et al., 2018), penalized regression (Ayers & Cordell, 2010; Cho et al., 2009; Hastie, 2020; Tibshirani, 1996), and Bayesian methods (Chen et al., 2015; Guan & Stephens, 2011; Hormozdiari et al., 2014; Zou et al., 2022). Many of these approaches can work on either individual-level data or GWAS summary statistics (Benner et al., 2016; Newcombe et al., 2016; Verzilli et al., 2008; Yang et al., 2012a) that use reference samples to estimate the correlations between SNPs. Differences between the methods are due to variations in the assumptions for causal effects and algorithms for model selection (Benner et al., 2016; Newcombe et al., 2016; Verzilli et al., 2008; Yang et al., 2012a). For example, conditional stepwise selection is an automatic procedure of fitting regression models: at each step, a new SNP is evaluated and potentially added to or removed from the model based on its conditional significance that can be measured by conditional *p*-values, Bayesian information criterion (BIC), model $R^{2}$, and so forth. On the other hand, a Bayesian approach, FINEMAP, (Benner et al., 2016) places a Gaussian prior for causal effect estimates while the original implementation of the Joint Analysis of Marginal SNP Effects (JAM) (Newcombe et al., 2016) invokes a Cholesky transformation on the linear regression likelihood and adopts a *g*-prior for effect estimates. Existing fine-mapping approaches still faces challenges such as instability of model selection or computational inefficiency when there are a large number of high-correlated SNPs. For example, conditional stepwise selection can be very unstable with a large number of highly correlated SNPs resulting in *p*-values of the signals in the final selected model that tend to be conservative (Schaid et al., 2018). Penalized regression, such as lasso (Tibshirani, 1996) and elastic net (Cho et al., 2009), are potentially more stable because the penalty term encourages shrinkage of effect estimates towards zero resulting in sparsity and robust estimation. However, penalized models often do not perform well with highly correlated SNPs and they do not represent the uncertainty in effect estimation and model selection (Schaid et al., 2018). Fully Bayesian methods such as CAVIAR (Hormozdiari et al., 2014) can capture uncertainty in model selection by computing posterior probabilities for models within the model space. However, their computational demands grow rapidly with increasing number of SNPs.

Leveraging the information across multiple ethnic groups or ancestry populations can enhance the power of fine-mapping (Asimit et al., 2016; Conti et al., 2021; Mahajan et al., 2014). The larger sample size benefited from aggregating results from multiple studies across populations may lead to the identification of novel signals with small effects or rare frequencies that might be missed by individual studies. In addition, different ancestry groups may have distinct LD structures due to different evolutionary and migration histories (Kichaev & Pasaniuc, 2015; Teo et al., 2010). For example, compared to non-African ancestry populations, African ancestry populations have smaller LD blocks with weaker correlations as the number of recombination events for each region is expected to be higher (Campbell & Tishkoff, 2008), making it easier to disentangle the LD structure and pinpoint potential causal signals. Therefore, integrating the difference in the LD structures across populations can potentially narrow the credible set that a causal variant resides in and improve the resolution of the fine-mapping signal (Hu et al., 2017; Morris, 2011).

Leveraging previous work on the joint analysis of GWAS summary statistics (Jiang et al., 2021; Newcombe et al., 2016), we present an extension of single-population fine-mapping through JAM to a multipopulation setting by fitting a 2-stage hierarchical model, “mJAM.” The first stage models the joint SNP-outcome relationship within a population where the second stage models the population-specific effect and the overall effect in a fixed-effect model. The mJAM likelihood allows for different feature selection procedures to be performed on summary statistics obtained from multiple populations. This includes Bayesian variable selection approaches that also yield credible sets or more conventional approaches for only selecting certain SNPs. In addition, we propose a novel definition of credible set probability that can be used to construct credible sets given a set of index variants in a multipopulation analysis. We illustrate this flexibility with two computationally efficient implementations of mJAM: “mJAM-SuSiE” for Bayesian variable selection with native SuSiE credible sets, and “mJAM-Forward” for frequentist forward selection of index SNPs and subsequent credible set construction. Through simulation studies with realistic effect size and various patterns of LD, we compare mJAM-SuSiE and mJAM-Forward with other multipopulation approaches, including the most commonly used fixed-effect meta-analysis, COJO (Yang et al., 2012a) with ancestrally pooled LD structure and meta-analyzed summary statistics, and MsCAVIAR (Lapierre et al., 2021), a Bayesian fine-mapping approach that allows for an arbitrary number of causal variants in a region. We then applied these methods to three known risk regions for prostate cancer to demonstrate the practical advantages of mJAM.

## 2 | MATERIALS AND METHODS

### 2.1 | Multipopulation JAM

To simplify notation and without loss of generality, we consider the scenario with three populations. Within each population and for a given set of p SNPs within each region, a linear phenotypic model is used.

(1) $$y^{\left(i\right)}=G^{\left(i\right)}\beta^{\left(i\right)}+\epsilon^{\left(i\right)},\;\text{for}\;i=1,\;2,\;3,$$

where $y^{\left(i\right)}$ is a $N^{\left(i\right)}\times 1$ vector of mean-centered phenotypic trait values for the $i^{th}$ population, with $N^{\left(i\right)}$ being the GWAS sample size of the $i^{\text{th}}$ population; $G^{\left(i\right)}$ is a $N^{\left(i\right)}\times p$ matrix of individual-level genotype data for the $i^{th}$ population, where each SNP has been centered to its mean to avoid fitting an intercept; $\beta^{\left(i\right)}\in R^{P}$ denotes the joint effect of the given set of p SNPs in the $i^{\text{th}\;}$ population. $\epsilon^{\left(i\right)}\;\sim \;N\left(0,\sigma^{2}I_{N^{\left(i\right)}}\right)$ is the random error in the linear phenotypic model whereas $\sigma^{2}$ is the variance of the error term, that is, assumed to be the same across all populations.

Under a mixed-effect model, the relationship between population-specific joint effect $\beta^{\left(i\right)}$ and the pooled effect, denoted as $\beta_{\mathrm{global}}$, can be expressed as

(2) $$\left(\begin{array}{l} \beta^{\left(1\right)}\\ \beta^{\left(2\right)}\\ \beta^{\left(3\right)} \end{array}\right)=\left(\begin{array}{c} I_{P}\\ I_{P}\\ I_{P} \end{array}\right)\beta_{\text{global}}+\delta ,$$

where $I_{P}$ is a p by p identity matrix and δ is a 3p×1 vector of random error that captures the deviation of population-specific effect $\beta^{\left(i\right)}$ from the pooled effect $\beta_{\text{global}}$, that is, the between-population heterogeneity of the effect sizes. When δ=0, Equation (2) is reduced to a fixed-effect model where the true SNP effects are the same across populations.

In multipopulation JAM, we assume δ=0 and adopt a fixed-effect approach. Then Equations (1) and (2) together form a two-stage model when individual-level data are available. The first stage is three separate linear phenotypic models whereas the second stage fits a fixed-effect meta-analysis model that combines all populations together. By replacing the $\beta^{(i)\prime }s$ in Equation (1) with Equation (2), Equation (1) then becomes

(3) $$\begin{array}{l} y^{\left(i\right)}=G^{\left(i\right)}\beta^{\left(i\right)}+\epsilon^{\left(i\right)}=G^{\left(i\right)}\left(I_{P}\beta_{\text{global}}+\delta^{\prime }\right)+\epsilon^{\left(i\right)}\\ \;=G^{\left(i\right)}I_{P}\beta_{\text{global}}+\epsilon^{\left(i\right)}, \end{array}$$

where $\delta^{\prime }$ represents the same source of error as δ but with length p, and $\delta^{\prime }=0$ by the fixed-effect model assumption. Equation (3) can also be written in a form akin to a mega-regression that incorporates the individual-level data of all populations:

(4) $$\left(\begin{array}{l} y^{\left(1\right)}\\ y^{\left(2\right)}\\ y^{\left(3\right)} \end{array}\right)=\left(\begin{array}{lll} G^{\left(1\right)} & 0 & 0\\ 0 & G^{\left(2\right)} & 0\\ 0 & 0 & G^{\left(3\right)} \end{array}\right)\left(\begin{array}{l} I_{P}\\ I_{P}\\ I_{P} \end{array}\right)\beta_{\mathrm{global}\;}+\epsilon^{\prime },$$

where $\epsilon^{\prime }\;\sim \;N\left(0,\sigma^{2}I_{N}\right)$ f and $N=\sum_{i}N^{\left(i\right)}$ is the random error in the combined linear phenotypic model. With summary data in which only the marginal effect sizes and their standard errors are available, it is also possible to estimate the joint effect size, $\beta_{\mathrm{global}}$, with an additional reference sample that estimates the correlation structures between the SNPs (Schaid et al., 2018; Yang et al., 2012b). Thus, Equation (4) can be written with only GWAS summary statistics to form a modified mJAM likelihood after linear transformation:

(5) $$\begin{array}{c} {\left(G_{c}I_{c}\right)}^{\prime }y_{c}\;\sim \;N\left(\left({\left(G_{c}I_{c}\right)}^{\prime }G_{c}I_{c}\right)\beta_{global},\right.\\ \;\left.\sigma^{2}\left({\left(G_{c}I_{c}\right)}^{\prime }G_{c}I_{c}\right)\right) \end{array}$$

where $G_{c},y_{c},I_{c}$ denotes $\left(\begin{array}{lll} G^{(1)} & 0 & 0\\ 0 & G^{(2)} & 0\\ 0 & 0 & G^{(3)} \end{array}\right),\left(\begin{array}{l} y^{(1)}\\ y^{(2)}\\ y^{(3)} \end{array}\right)$ and $\left(\begin{array}{l} I_{P}\\ I_{P}\\ I_{P} \end{array}\right)$ in Equation (4) respectively. By expanding each matrix, we have $G_{c}I_{c}y_{c}=\sum_{i=1}^{3}\;G^{(i)\prime }y^{(i)}$ and ${\left(G_{c}I_{c}\right)}^{\prime }G_{c}I_{c}=\sum_{i=1}^{3}\;G^{(i)\prime }G^{(i)}$ where $G^{(i)\prime }G^{(i)}$ and $G^{(i)\prime }y^{(i)}$ are populationspecific statistics and can be estimated by populationspecific GWAS summary statistics and a reference genotype matrix. Population-specific missing variants in the summary statistics or in the reference genotype can also be incorporated while calculating $G_{c}I_{c}y_{c}$ and ${(G_{c}I_{c})}^{\prime }G_{c}I_{c}$. Detailed derivation can be found in Supporting Information Methods.

### 2.2 | Index SNP selection and credible set construction for fine mapping

mJAM establishes a multi-SNP model within each population with corresponding population-specific LD, while jointly estimating a fixed-effects summary estimate of effect. The mJAM likelihood, outlined in Equation (4), can be integrated into various existing feature selection methods, provided they are compatible with inclusion of the mJAM statistics (${(G_{c}I_{c})}^{\prime }G_{c}I_{c}$ and $G_{c}I_{c}y_{c}$ in Equation (5)). Possible approaches for index SNP selection in mJAM includes stepwise selection (Schaid et al., 2018), Ridge regression (Hastie, 2020), and Bayesian approaches such as the “Sum of Single Effects” model (SuSiE) (Zou et al., 2022).

We adopt a forward selection approach based on conditional *p*-value for index SNP selection because of its computational efficiency and straightforward interpretation. We define our implementation of “mJAM-Forward” as a two-step approach in which a first step relies on a conventional stepwise forward selection to select an additional index SNP based on its corresponding *p*-value from a mJAM model conditional on any previous index SNP(s). We incorporate a *g*-prior to stabilize effect estimates (Goel & Zellner, 1986) (details in Supporting Information Methods). $p=5\times 10^{-8}$ is used as the default threshold because it aligns with the conventional genome-wide significance threshold in most GWAS studies. And the variants selected can be interpreted as “conditionally genome-wide significant” where “conditionally” means conditional on previous index SNPs in a region (if there is any). Setting a stringent conditional *p*-value threshold (i.e., smaller *p*-value) will make mJAM-Forward more conservative in terms of index SNP selection while a looser threshold (i.e., larger *p*-value) can possibly select more variants with weaker conditional significance at the risk of including more false positives.

To ensure stable estimates in forward selection under the likelihood presented in Equations (3)–(5), we avoid fitting models with highly correlated SNPs by including a LD pruning process in mJAM-Forward (Algorithm 1). Every time a new index SNP is selected, and its credible set is constructed, variants whose squared correlation $\left(r^{2}\right)$ with the index SNP exceeds a predefined threshold will be removed, and will not be considered for future rounds of index SNP selection. Since $r^{2}$ may differ across populations, the pruning threshold is determined by both a within-population cutoff (SNP will be pruned if its $r^{2}$ exceed this cutoff in *any* population) and an across-population cutoff (SNP will be pruned if its $r^{2}$ exceed this cutoff in *all* populations), both of which can be specified by users. The default we set is $r^{2}\geq 0.5$ for within-population and $r^{2}\geq 0.2$ for across-population.

**FIGURE 1:**
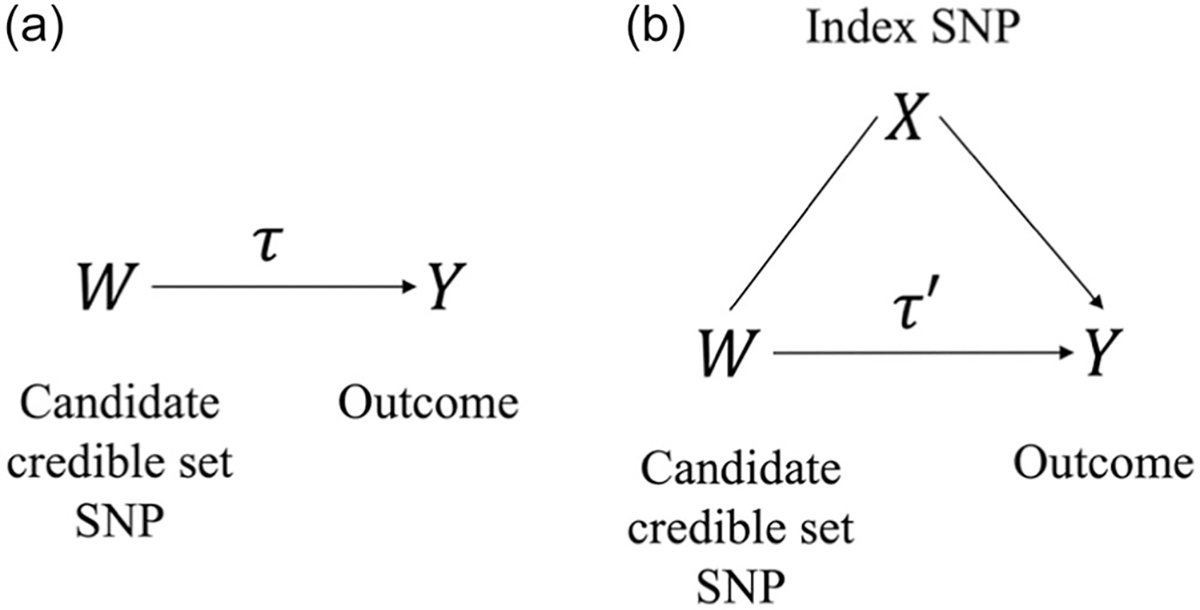
Conceptual graph of the correlations between index single-nucleotide polymorphisms (SNP), candidate credible set SNP, and the outcome. (a) Model with the candidate credible set SNP, W. τ is conceptually the “total effect” of W on Y. (b) Model with W and X, the index SNP. τ′ is conceptually the “direct effect” of W on Y.

The second step for mJAM-Forward is to define a multipopulation credible set for each index SNP. Instead of using $r^{2}$, which can vary a lot by populations, to identify credible set SNPs for a given index SNP, we propose a novel probability to define credible set SNPs with effect across populations that are captured by a given index SNP (Figure 1). Here, we fit two mJAM models for each candidate credible set SNP, W, located within a region of an index SNP, X. These models demonstrate that the candidate credible set SNP is: (1) associated with phenotype marginally and (2) that the index SNP mediates the effect of the candidate SNP on the phenotype. The first model includes W by itself to yield a probability that W is associated with the phenotype. This model also provides a posterior distribution for the marginal effect for the candidate credible set SNP.

(6) $$Pr\left(M_{W}|\mathrm{Data}\right)=\frac{p\left(M_{W}\right)BF\left[M_{W}\;:\;M_{\mathrm{Null}}\right]}{\sum_{w}p\left(M_{W}\right)BF\left[M_{W}\;:\;M_{\mathrm{Null}}\right]}$$

where $p\left(M_{W}\right)$ is the prior density of one-SNP model that includes W and $BF\left[M_{W}:M_{\mathrm{Null}}\right]$ is the Bayes factor of one-SNP model with W to the null model. See Supporting Information Methods for detailed expression of $BF\left[M_{W}:M_{\mathrm{Null}}\right]$ with the incorporation of a *g*-prior of the effect estimates. The second model conditions on the index SNP, X, to obtain a posterior estimate for an adjusted effect estimate for the credible set SNP. Borrowing from a mediation framework (Robins & Greenland, 1992), we then calculate the probability that the index SNP mediates the candidate credible set SNP effect using the two models (Figure 1).

(7) $$Pr(\mathrm{Mediation}|\mathrm{Data})=Pr\left(\left|\tau_{W}-\tau_{W}^{\prime }\right|>|t||\tau_{W}=\tau_{W}^{\prime }\right)$$

where $\tau_{W}$ is the total effect of the candidate credible set SNP on the outcome, $\tau_{W}^{\prime }$ is the direct effect and t is the observed difference between the total effect and the direct effect. A strong mediation effect indicates that the observed marginal effect of the candidate credible set SNP on the phenotype is mainly due to its indirect effect through its strong correlation with the index SNP, and not due to a direct effect on the phenotype. These two model probabilities are then combined to calculate the probability that a candidate SNP is a credible set SNP, Posterior Credible Set Probability (PCSP).


(8) $$PCSP_{W}\;:=Pr\left(M_{W}|Data\right)\cdot Pr((\mathrm{Mediation}|\mathrm{Data})$$

PCSP are then scaled over all SNPs in the region and used to define a ρ-level credible set of cross-population SNPs. The default is to use 95% credible set.

**Algorithm 1.** Pseudo algorithm for fitting mJAM-Forward and credible set construction in a region

Input data: ${\overset{ˆ}{\beta }}^{\left(i\right)},\;se({\overset{ˆ}{\beta }}^{\left(i\right)})$, sample size of GWAS $N_{GWAS}$ Effect Allele Frequencies ${EAF}^{\left(i\right)},$ $G_{R}^{(i)}$ for each study indexed by *i*Input arguments: LD threshold for index SNP selection, conditional *p*-value threshold	
1.	*Compute mJAM statistics* ${\left(G_{c}I_{c}\right)}^{\prime }G_{c}I_{c},{\left(G_{c}I_{c}\right)}^{\prime }y_{c}$ *and* $y_{c^{\prime }}y_{c}$.
2.	*Compute marginal mJAM P-values under g prior specification for all testing SNPs in the region*.
3.	*While the smallest conditional P-value (or marginal P-value in the first round only) is smaller than given threshold:*
4.	*Identify the testing SNP with the smallest conditional P-value as the next index SNP*.
5.	*Construct credible set of the new index SNP*.
6.	*Prune out SNPs in LD with the new index SNP based on LD threshold*.
7.	*Compute the conditional mJAM P-value for all remaining SNPs in the region*.
8.	*Stop until no SNP in the region has conditional P-value smaller than threshold*.
	*Return index SNP(s), corresponding conditional* p-*value(s) and credible set(s)*.

We also integrate the mJAM likelihood and summary statistics into a Bayesian selection method that indicates index SNPs and simultaneously estimates credible set SNPs, “mJAM-SuSiE” (Wang et al., 2020). The “Sum of Single Effects” Model (SuSiE) provides an alternative approach for variants selection by modeling the common joint effect $\beta_{global}$ in Equations (4) and (5) as a sum of “single-effect” vectors, each with one non-zero effect. The susieR package (Zou et al., 2022) provides the implementation of SuSiE algorithm not only with inputs as individual-level data, but also with inputs as sufficient statistics (statistics that are sufficient for estimating the regression coefficients under the SuSiE model). For implementation with mJAM, the form of SuSiE sufficient statistics is essentially the same as that of mJAM statistics $\left({(G_{c}I_{c})}^{\prime }G_{c}I_{c}\;\text{and}\;{(G_{c}I_{c})}^{\prime }y_{c}\right)$ in Equation (5). See Supplemental Algorithm 1 in Supporting Information Methods for the pseudo-algorithm of fitting mJAM-SuSiE in detail.

### 2.3 | Simulation studies

We conducted a simulation study to compare the performance of the two mJAM implementations (mJAM-SuSiE and mJAM-Forward) with three commonly used alternative approaches: fixed-effect meta-analysis (“FE”; an inverse-variance weighted average of the marginal estimates), COJO (Yang et al., 2012a) (a conditional stepwise selection based on conditional *p*-values) and MsCAVIAR (Lapierre et al., 2021) (a multipopulation extension of the Bayesian fine-mapping approach “CAVIAR”). We performed two sets of scenarios: (1) simulated correlation structures with the same block LD structures across populations (Figure S1); and (2) simulated correlation based on real genetic correlation structures observed in the study cohort from Elucidating Loci Involved in Prostate Cancer Susceptibility (ELLIPSE) OncoArray Consortium (Conti et al., 2021) (Figure S2).

We evaluated fine-mapping model performance through two perspectives: the performance of index SNP selection and the performance of credible sets. To compare the performance of index SNP selection across these multipopulation approaches, we use three metrics: number of selected index SNPs, sensitivity/power, and positive predictive value (PPV). To be more specific, sensitivity of index SNP selection is defined as the proportion of 500 simulations where the true causal SNP was selected in an index SNP. PPV of index SNP selection is defined as the proportion of causal SNPs selected as an index over all selected index SNPs, averaged over 500 simulations. For mJAM-SuSiE and mJAM-Forward, index SNP is defined as the SNP with the highest posterior probability in each 95% credible set. MsCAVIAR outputs one “causal set” of SNPs that contain *all* causal SNPs with probability at least 95%. The interpretation of a causal set deviates from that of a credible set when the number of causal variants in a set is greater than one. Thus, for comparing credible set performance only, we defined the index SNP of a MsCAVIAR causal set as the SNP with top-m SNPs with the highest posterior probability of having a causal effect if there are m true causal SNPs in simulations. When comparing size of credible/causal sets across methods, we used average set size for MsCAVIAR, which is defined by the size of a 95% MsCAVIAR set divided by the number of true causal variant(s) in simulation (this is also the number of maximum causal variants set in the MsCAVIAR program). In addition, since MsCAVIAR, mJAM-SuSiE, and mJAM-Forward output credible set(s) within each region, we compared credible set performance through the size of each credible set, sensitivity/power, PPV, and empirical coverage. The sensitivity of credible set is defined as the proportion of 500 simulations where the true causal SNP was selected in a credible set whereas PPV refers to the number of true causal SNP(s) selected in a credible set, if any, divided by the corresponding credible set size, averaged over 500 simulations. Since FE is a non-Bayesian approach and it does not perform joint analysis, we considered the group of SNPs with meta-analyzed *p*-values less than a Bonferroni-corrected significance level as a single credible set for the purpose of performance comparison.

### 2.4 | Applied examples

To illustrate mJAM on real data, we applied the methods on three regions using summary statistics from the latest cross-ancestry prostate cancer association study (Wang et al., 2023) across four ancestry groups, including 122,188 prostate cancer cases and 604,640 controls of European ancestry, 19,391 cases and 61,608 controls of African ancestry, 10,809 cases and 95,790 controls of East Asian ancestry, and 3931 cases and 26,405 controls from Hispanic populations. Within each region, we applied mJAM-Forward to select index SNP(s) using population-specific summary statistics and reference dosage for each population. Then we constructed mJAM credible set(s) by including top SNPs ranked by their mJAM posterior probabilities until those SNPs included in the credible set reached a cumulative posterior probability of 95%. LD reference panels were obtained from the Prostate Cancer Association Group to Investigate Cancer-Associated Alterations in the Genome and Collaborative Oncological Gene-Environment Study Consortium [PRACTICAL iCOGS], the Elucidating Loci Involved in Prostate Cancer Susceptibility OncoArray Consortium [ELLIPSE OncoArray] (Conti et al., 2021), leading to a total of 89,627 samples of European ancestry, 8303 samples of African ancestry, 1479 samples of East Asian ancestry and 2249 samples of Hispanic population. Results from mJAM-Forward are also compared with those from mJAM-SuSiE, COJO, and MsCAVIAR. Analysis was restricted to SNPs with marginal meta-analyzed *p*-value <10^−3^ and minor allele frequency (MAF) >2%. All the SNP positions in the following sections are based on genome build version of GRCh37/hg19.

## 3 | RESULTS

### 3.1 | Simulation study on artificial LD

Under the baseline scenario (50 SNPs in total, one causal SNP with an effect size of 0.03 which is equivalent to heritability of 0.03%, three studies per population, and balanced sample size across populations), the 95% credible sets from mJAM-Forward, mJAM-SuSiE, and MsCAVIAR were well calibrated to the specified coverage level (Figure S3). Similarly, the SNP-level probabilities produced by these three methods are well calibrated and adhered to the expected values, as shown in Figure S4a. Moreover, all three methods exhibited robust performance in distinguishing true causal SNPs from non-causals based on SNP-level probability under the baseline scenario with independent LD (Figure S4b), as shown by the average SNP-level probability of true causal SNPs being substantially higher than that among noncausal SNPs. It is also worth noting that the SNP-level posterior probabilities in mJAM-Forward credible sets (i.e., posterior credible set probability or PCSP) are unique in combining information on the marginal association between the credible set SNP and the outcome and the mediation effect of the index SNP on the candidate SNP, as detailed in the methods section. The PCSP can also be viewed as a weighted version of the model probability $Pr\left(M_{W}|\right.Data)$ where the weights are the mediation probability $Pr\left(|\tau_{W}-\tau_{W}^{\prime }|>|t||\tau_{W}=\tau_{W}^{\prime }\right)$. By assigning differential weights to the model probabilities, mJAM-Forward’s credible set size is reduced because of the prioritization of SNPs with a higher mediation probability (Figure S5).

**FIGURE 2:**
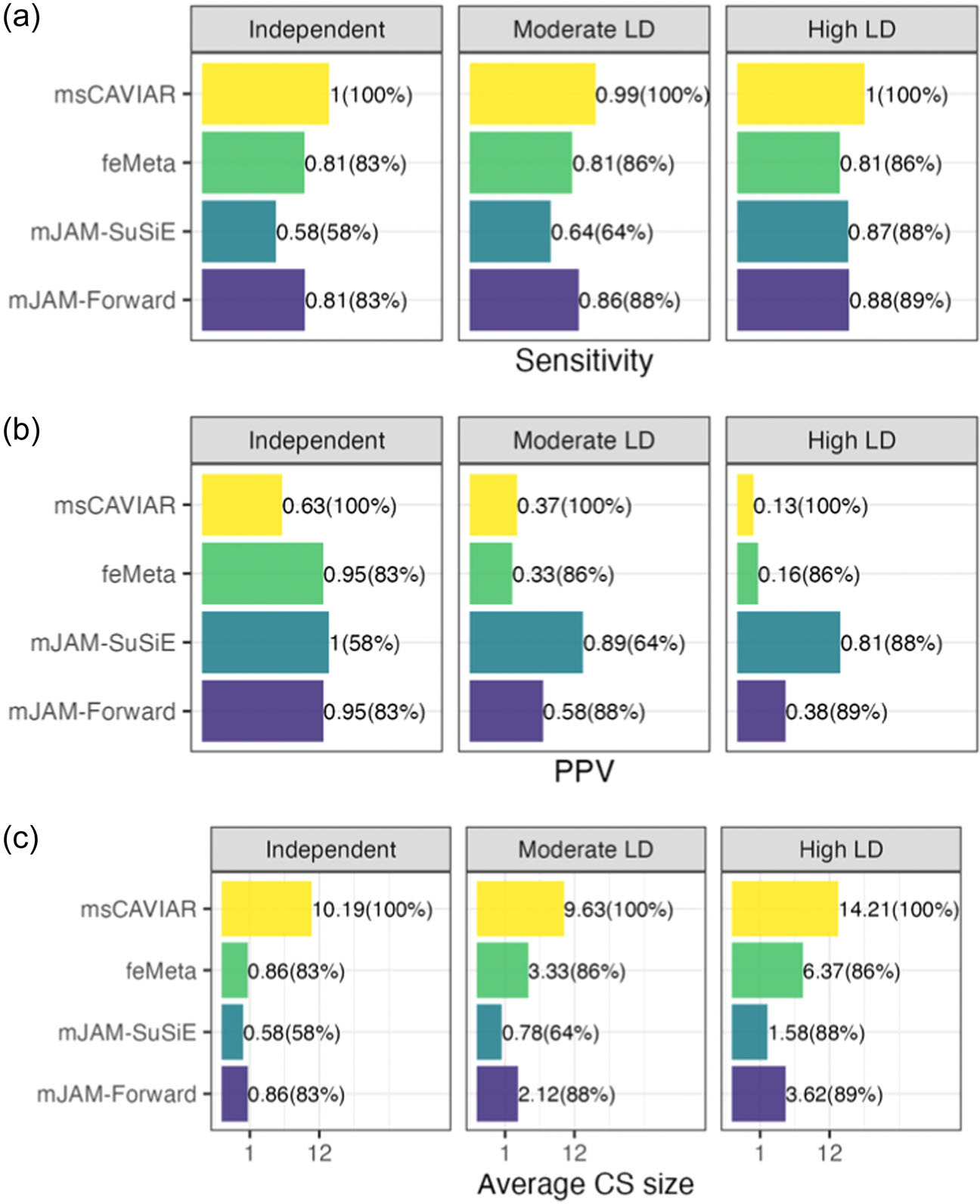
Credible set performance in simulation studies with artificial linkage disequilibrium structure. (a) Sensitivity, that is, the proportion of 500 simulations where the true causal single-nucleotide polymorphisms (SNP) was selected in a credible set. (b) Positive Predictive Value (PPV), that is, the proportion of true causal SNP over the credible set size, averaged over 500 iterations. (c) Average CS size. Brackets indicate the percentage of 500 simulations in which at least one credible set was returned by the corresponding method. If no credible set was returned in a simulation, then sensitivity was defined as 0, PPV as N/A (meaning this simulation does not contribute towards the average PPV shown in the figure), and the credible set size as 0.

Both mJAM-Forward and mJAM-SuSiE preserved relatively high sensitivity in terms of including the true causal SNP in its credible set (sensitivity = 0.86 and 0.64 respectively, Figure 2a). Although MsCAVIAR had the highest sensitivity (0.99) under the baseline scenario, its average credible set size was much larger (9.63 for MsCAVIAR; 2.12 for mJAM-Forward and 0.78 for mJAM-SuSiE, Figure 2c), thus leading to a much lower PPV (0.37, Figure 2b). mJAM-SuSiE had the highest PPV (0.89) among the methods we compared, meaning that it had the highest proportion of true causal over the total number of credible set SNPs on average, followed by mJAM-Forward (0.58) (Figure 2b). In terms of credible set sensitivity, PPV, and average CS size the methods had similar patterns of performance for scenarios expanded beyond the baseline to various LD structures, imbalanced sample size across populations, three causal SNPs (Figure S6), varying number of population-specific studies per population (Figure S7), and larger regions with 1000 SNPs (Figure S8).

**FIGURE 3:**
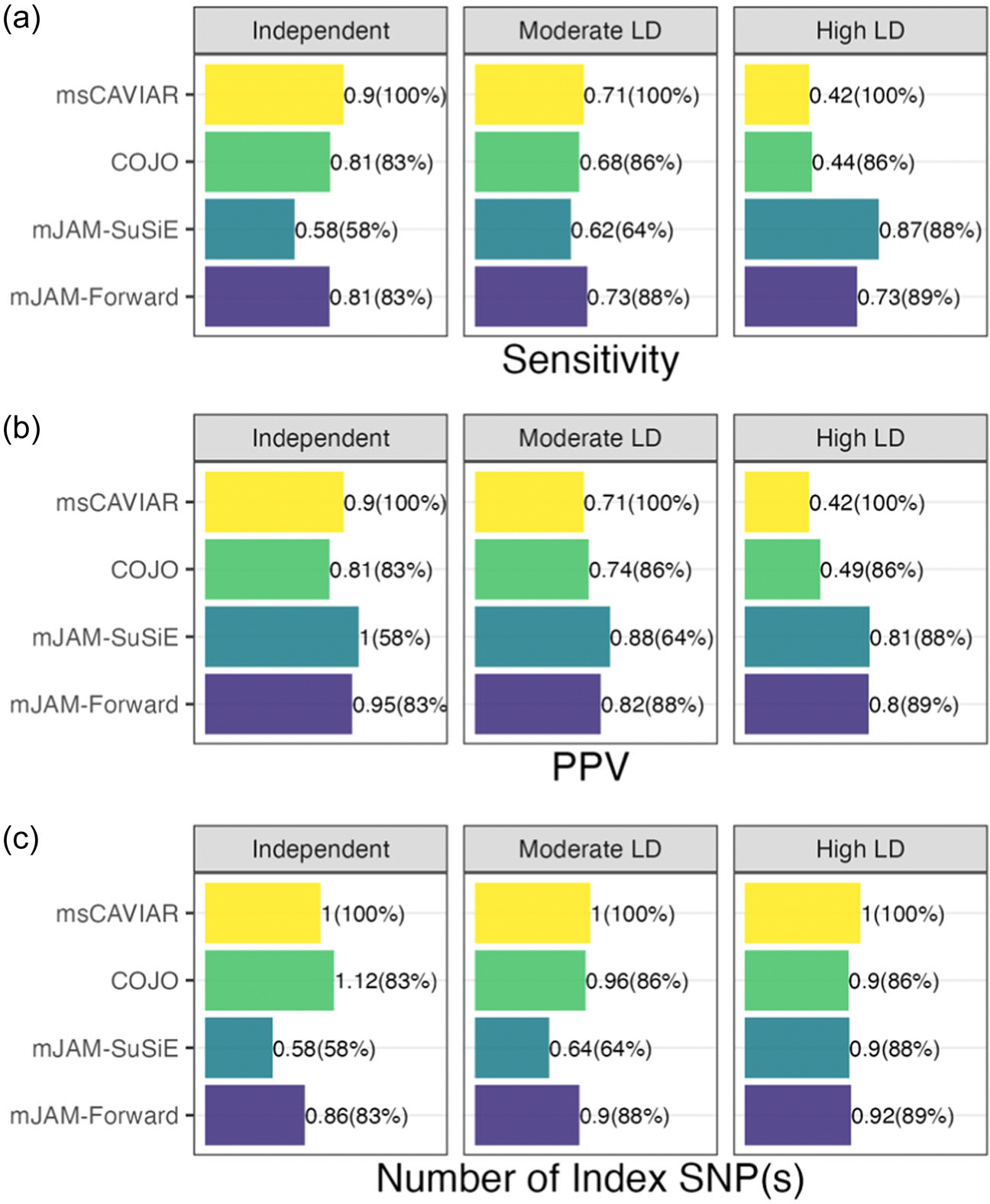
Performance of index single-nucleotide polymorphisms (SNP(s)) selection in simulation studies with artificial linkage disequilibrium structure. (a) Sensitivity, that is, the proportion of 500 simulations where the true causal SNP was selected in an index SNP. (b) Positive Predictive Value (PPV), that is, the proportion of causal SNP selected as an index over all selected indices, averaged over 500 iterations. (c) Number of index SNP(s) selected, averaged over 500 iterations. Brackets indicate the percentage of 500 simulations in which at least one credible set was returned by the corresponding method. If no index SNP was returned in a simulation, then sensitivity was defined as 0, PPV as N/A (meaning this simulation does not contribute towards the average PPV shown in the figure), and the number of index SNP as 0.

In terms of identifying the true causal variant as an index SNP (i.e., sensitivity), mJAM-Forward and MsCAVIAR had the best performance under moderate LD scenarios (Figure 3a) with a sensitivity was 0.73, and 0.71 respectively. However, for these two methods, mJAM-Forward had a better PPV (0.82) compared to MsCAVIAR (0.71). In comparison, mJAM-SuSiE had poor sensitivity (0.62) but a higher PPV (0.88). COJO had a similar performance with mJAM-Forward under independent LD scenarios but its sensitivity and PPV worsened compared to mJAM-Forward as the level of LD increases. Though COJO performs a similar stepwise selection as mJAM-Forward, unlike mJAM-Forward that specifically accounted for population-specific LD, COJO uses meta-analyzed marginal summary statistics and pooled LD panel which makes it difficult to identify the true common variants through disentangling the population-specific LD structure. All methods selected on average 1 index SNP among 500 simulations, close to the true number of causals (Figure 3c).

When the pairwise correlation within each LD block increased, the average credible set sizes for all methods increased correspondingly (Figure 2c). As a result, under high LD scenarios, the PPV of identifying the true causal (s) in a credible set decreased to a noticeable extent for MsCAVIAR, mJAM-Forward, and FE (Figure 2b). Though mJAM-Forward’s PPV dropped due to the increase in credible set sizes on average, mJAM-Forward was still able to retain a sensitivity of 0.88 under the high LD scenario. mJAM-SuSiE achieved the highest PPV (0.81, Figure 2b) among all methods under high LD scenarios while retaining relatively high sensitivity and small credible set size. However, mJAM-SuSiE’s sensitivity was relatively low compared to mJAM-Forward and MsCAVIAR under independent or moderate LD scenarios (Figure 2a).

The difference in credible set sensitivity and PPV, as discussed above, is partially due to the different approaches have different handling of low confidence credible sets when the power is low. MsCAVIAR produces one set of variants that collectively contain m causal variants with high probability where m is the user-defined maximum number of causal SNPs in a region. When there is not sufficient power to detect the signal in the observed data, MsCAVIAR may still produce a causal set but the size of MsCAVIAR causal set will increase to capture the uncertainty. On the other hand, mJAM-Forward, mJAM-SuSiE, COJO, and FE may return no significant results at all when the power is low. Under the baseline scenario, mJAM-SuSiE did not select any index SNP in 36% of the simulations; mJAM-Forward in 12%, COJO in 14%, and FE in 14%. These two different ways of capturing high uncertainty or showing low confidence result in MsCAVIAR being compromised more in terms of PPV, mJAM-SuSiE being compromised more in terms of sensitivity and some averaged credible set size smaller than 1 in Figure 2. To remove the artifacts due to the discrepancy in the reporting of MsCAVIAR versus other methods, we restricted the analysis to only the simulations when all methods return at least one credible set. Alternatively, we reported the SNP with the smallest marginal *p*-value from the fixed-effect meta-analysis (i.e., the lead SNP) instead when an approach returned no credible sets at all, as often done in practice. As shown in Figure S9, under either of these two alternative analyses, there is noticeable improvement in terms of credible set sensitivity for all approaches except MsCAVIAR. MsCAVIAR’s PPV showed substantial improvements under independent and moderate LD scenarios when we restricted the analysis to the subset where all approaches returned at least one credible set while mJAM-SuSiE’s PPV remained highest across all LD scenarios.

**FIGURE 4:**
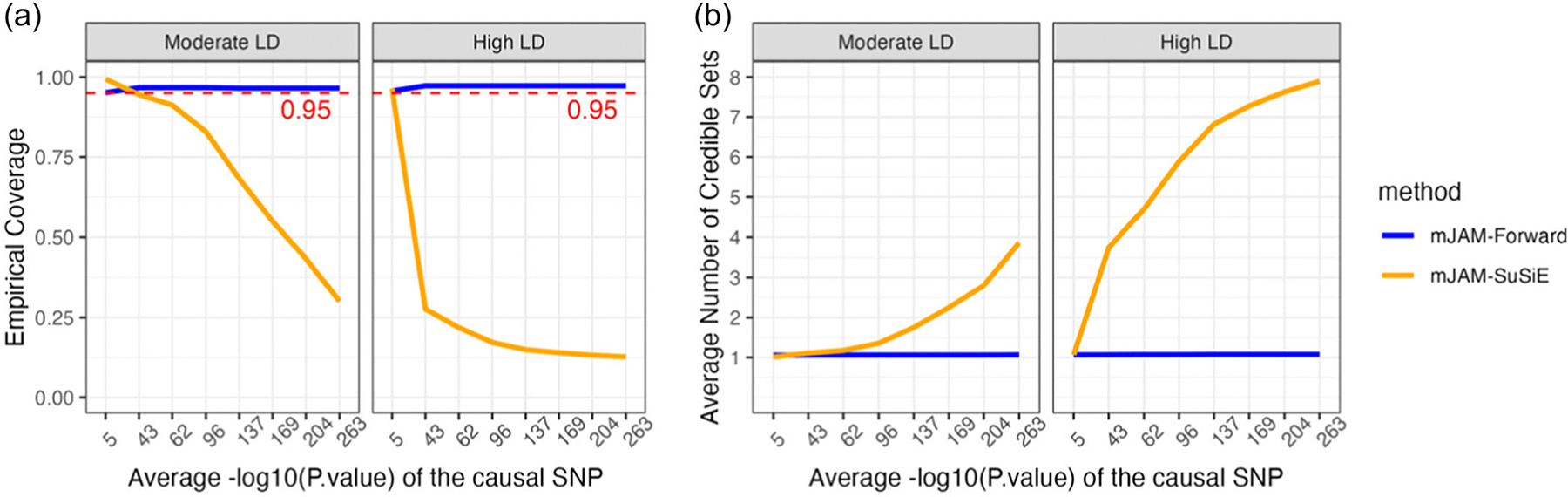
Credible set behavior of mJAM-SuSiE and mJAM-Forward as causal single-nucleotide polymorphism (SNP) significance increases. Simulations were conducted under the baseline scenario setting (one causal SNP out of 50 SNPs in total which are divided into five linkage disequilibrium blocks) with varying effect sizes. As a result of increasing effect sizes, the average empirical −log10(*p*-value) of the causal SNP ranged from 5 to 263, covering most situations seen in practice. Red dashed line indicates requested coverage which is set to be 0.95 for both methods. (a) Empirical credible set coverage; (b) average number of credible sets selected among 500 simulations.

Despite mJAM-SuSiE’s outstanding performance under high LD scenarios with moderate causal effect size, we noticed that its results were very sensitive to the marginal significance of the true causal SNPs. To represent a real-life situation where a lead variant within a region has an extremely significant marginal *p*-value, we expanded the simulation study with increasing significance of the true causal SNP by simulating larger effect size while keeping other settings the same as the baseline scenario with 1 true causal SNP. As a result, the average −log10(*p*-value) of the true causal ranges from 5 to 263 (mimicking significance often found in well-powered GWAS). Under increasingly high-power scenarios, mJAM-Forward consistently selected 1 credible set regardless of the significance of the true causal whereas the average number of credible sets by mJAM-SuSiE increased as the statistical significance (i.e., effective power) increased (Figure 4b). As a result, mJAM-SuSiE selected more false positive SNPs within the credible sets when the true causal SNP has high observed marginal significance. In addition, the empirical coverage of mJAM-SuSiE’s credible sets dropped below the expected level quickly after the true causal SNP became more significant (Figure 4a). In contrast, mJAM-Forward’s credible sets remained well-calibrated. As shown in Figure S10, when the true causal variant has a large effect size, the number of mJAM-SuSiE’s credible sets is sensitive to the number of non-zero effects allowed (one of the parameters in native SuSiE algorithm, denoted as L). One possible way to control such inflation depicted in Figure 4 is to set L to exactly 1, as shown in Figure S10. However, it is worth noting that setting L=1 poses a key assumption that there is at most one signal in a region, which may not always be a reasonable assumption in practice. Therefore, we don’t recommend setting L=1 in practice unless there is enough prior/external evidence to support that this is only 1 potential causal variant in the region.

To explore the impact of two types of missingness on the performance of mJAM-Forward, we modified our simulation studies with artificial LD structure to include a missing SNP in LD with the causal SNP, or with the missing SNP as the causal SNP itself. The flexibility of mJAM likelihood (Equation 2) allows us to incorporate SNPs with missing information in some studies or populations in the analysis. We found that when the missing SNP is in LD with the causal SNP, mJAM-Forward has stable performance in comparison to when there is no missingness (Figure S11). When the causal SNP is missing, mJAM-Forward still preserves the power both to select the causal SNP as the index SNP and to include the causal SNP in its credible set.

Though mJAM is developed based on a continuous outcome (Equations (1)–(5)), we also explored its application to summary statistics form a binary outcome in the form of log odds ratios and corresponding standard errors. To fairly compare the performance of all methods under a linear-outcome to that under a binary-outcome, the effect size (in terms of log odds ratios) was specifically chosen to yield about 80% empirical power for identifying the causal variants through a fixed-effect meta-analysis. As shown in Figure S12, the relative performance between approaches (both in terms of credible sets and index SNPs) remained similar when all these approaches were applied to binary-outcome summary statistics.

### 3.2 | Simulation study on real LD

When applied to the simulated data on the 120-SNP region on chromosome 2, mJAM-Forward, mJAM-SuSiE, and MsCAVIAR selected on average around one index SNP whereas COJO selected 1.5 index SNPs, indicating a slight increase in false positive signals. mJAM-Forward had highest sensitivity and PPV of identifying the true causal from a complicated LD structure as an index SNP (Table 1). In terms of credible set performance, MsCAVIAR demonstrated high empirical coverage of its credible set as well as high sensitivity compared to the other two mJAM methods. However, such high sensitivity and PPV was achieved at the cost of a much larger size for the credible sets. The average size of the 95% CS of MsCAVIAR is 56.52, even larger than the number of SNPs that reached marginal genome-wide significant (5 × 10^−8^) in a fixed-effect meta-analysis (48.88). On the other hand, the average credible set size for mJAM-Forward and mJAM-SuSiE was 19.70 and 18.37 respectively. Meanwhile, both approaches preserved reasonably high sensitivity and empirical coverage.

### 3.3 | Applied example 1: A single-hit region on chromosome 12

**FIGURE 5:**
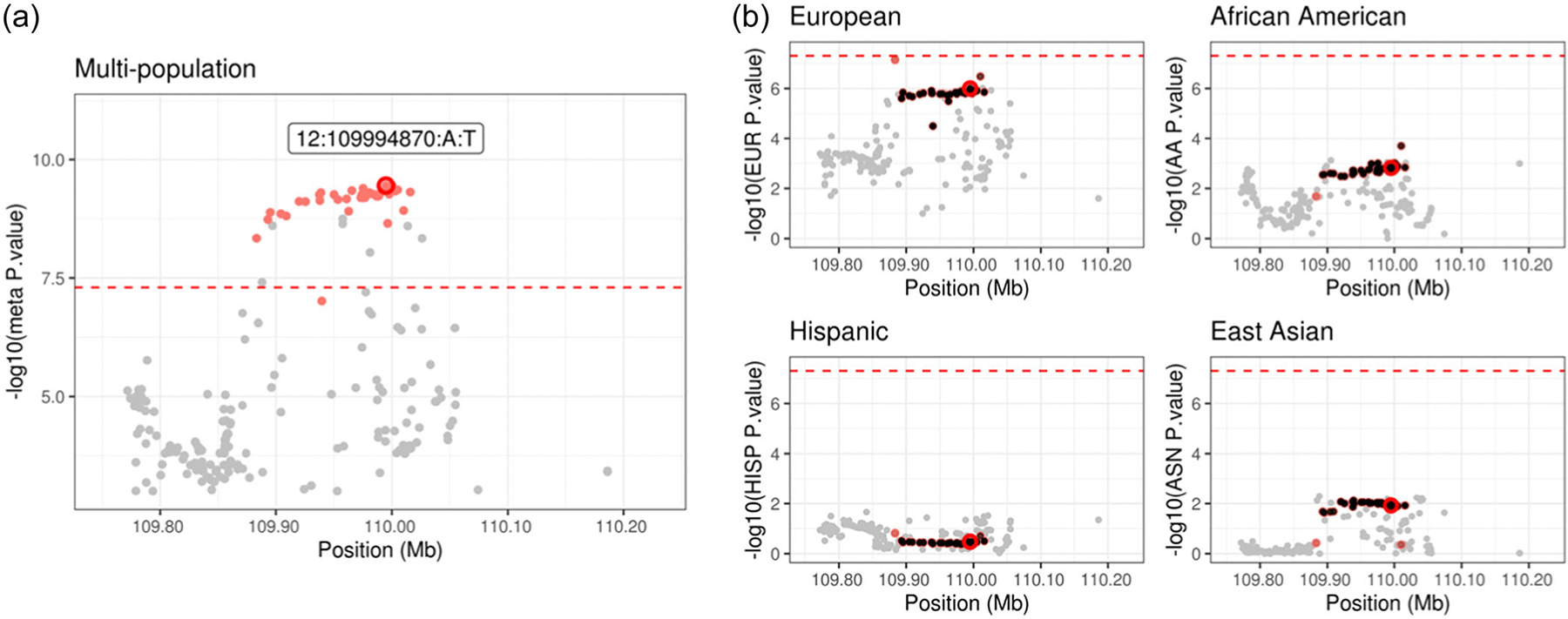
Manhattan plot for mJAM-Forward credible sets at chromosome 12 position 109194870 to 110794870. (a) *y*-axis is meta-analyzed −log10(*p*-value) from multipopulation analysis; index variant is labelled with GRCh37/hg19 reference assembly co-ordinates. (b) *y*-axis is −log10(*p*-value) from population-specific analysis.

The first applied example is a 1013 kb region on chromosome 12 which consists of 276 SNPs. The lead variant in this region, 12:109994870:A:T (genome build version: GRCh37/hg19) is a nonsynonymous mutation in *MMAB* which is reportedly upregulated in the tumor tissues of PCa samples (Quan et al., 2022). Figure S13 shows the LD structure for the four ancestry groups in this analysis. None of the SNPs in this region reached genome-wide significance in any population-specific analyses (Figure 5b) but after multipopulation meta-analysis 48 SNPs are genome-wide significant (Figure 5a). By setting a conditional *p*-value threshold at 5 × 10^−8^, mJAM-Forward identified one index SNP at 12:109994870:A:T (meta $p=3.5\times 10^{-10}$) with a corresponding 95% credible set of 41 SNPs. The median $r^{2}$ between the credible set SNPs with the index SNP is 0.998 for European LD, 0.979 for African, 0.990 for Hispanic and 0.996 for East Asian. COJO identified the same index SNP, 12:109994870:A:T. MsCAVIAR reported a slightly larger 95% credible set than mJAM-Forward, consisting of 45 SNPs (Figure S14A). The index SNP of MsCAVIAR’s credible set is 12:109998097:A:G (meta $p=3.7\times 10^{-10}$) whose $r^{2}$ with 12:109994870:A:T is greater than 0.99 in all four ancestry populations. This index SNP, 12:109998097:A:G, is included in a mJAM-Forward credible set only when coverage is increased to 99%; whereas the index SNP for mJAM-Forward, 12:109994870:A:T, is included in the 95% MsCAVIAR credible set. mJAM-SuSiE estimates a single 95% credible set with 28 total SNPs and a unique single index SNP, 12:109996343:A:C (meta *P*-value = 2.2 × 10^−9^) which is also included in both credible sets of mJAM-Forward and MsCAVIAR. The median $r^{2}$ within a credible set is also greater than 0.99 for all ancestry populations (Table S1). The index SNP from mJAM-Forward was also included in its credible set (Figure S14B).

### 3.4 | Applied example 2: Asian-driven signals on chromosome 10

**FIGURE 6:**
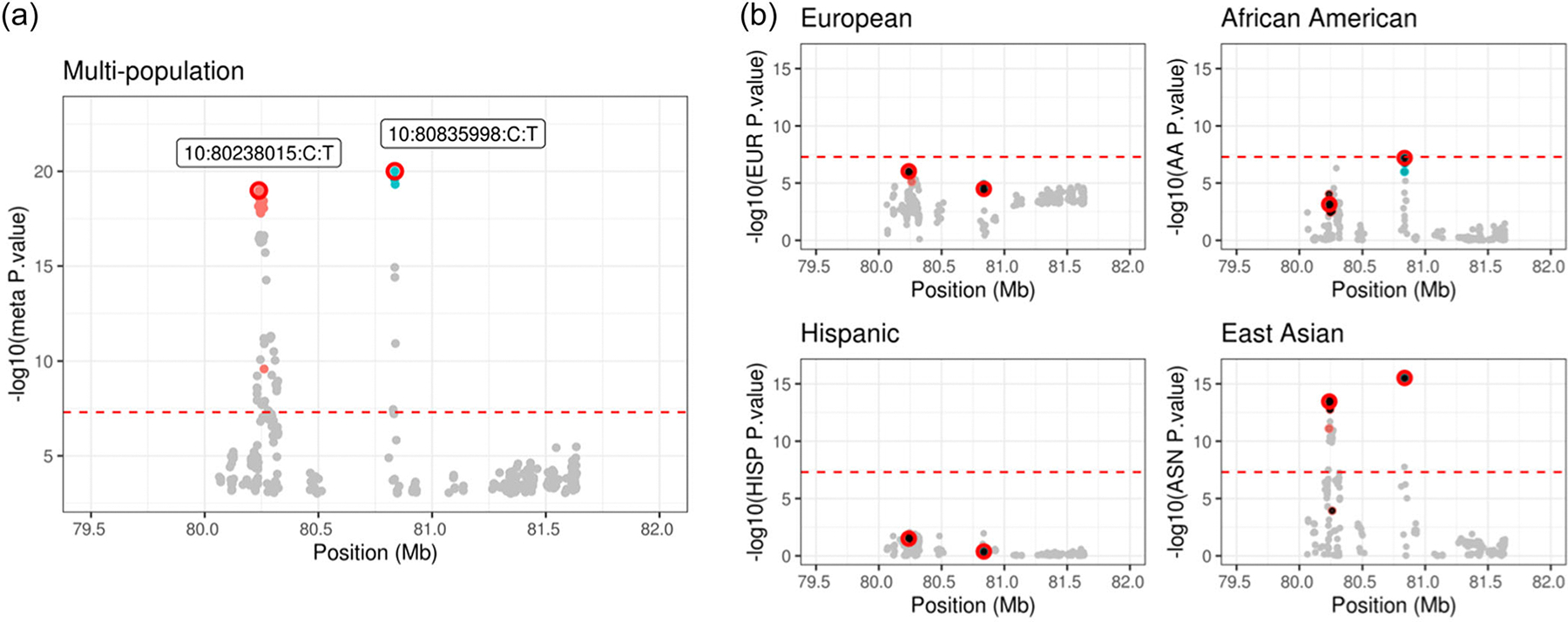
Manhattan plot for mJAM-Forward credible sets at chromosome 10 position 79436999 to 81635998. (a) *y*-axis is meta-analyzed −log10(*p*-value) from multipopulation analysis; index variants are labelled with GRCh37/hg19 reference assembly co-ordinates. (b) *y*-Axis is −log10(*p*-value) from population-specific analysis.

As a second example, we conducted an analysis on a chromosome 10 region which consists of 412 SNPs after QC and spans around 1571 kb. The lead variant in this region, 10:80835998:C:T, is one of the 269 variants associated with PCa in Conti et al. (2021) Figure S15 shows the LD structure in this region separately for European, African, East Asian, and Hispanic ancestry populations. This region contains two clear signals with meta-analyzed $p\;<10^{-15}$, which are mainly driven by the results from East Asian and African ancestry populations (Figure 6). In this example, mJAM-Forward identified two index SNPs, 10:80835998:C:T (meta $p=9\times 10^{-21}$) and 10:80238015:C:T (meta $p\;=1\times 10^{-19}$) (Figure 6a). The 95% mJAM-Forward credible set for the first index SNP, 10:80835998:C:T, contains three SNPs in total and there are 45 SNPs in the credible set for the second index SNP. The minimum $r^{2}$ between the mJAM-Forward credible set SNPs with its own index SNP is no less than 0.95 in European, East Asian, and Hispanic ancestry populations, and no less than 0.81 in African ancestry populations (Table S2). COJO identified two index SNPs, 10:80835998:C:T and 10:80240493:A:G. 10:80835998:C:T is the same as one of the index SNPs selected by mJAM-Forward and 10:80240493:A:G is included in the mJAM-Forward 95% credible set of 10:80238015:C:T. Since MsCAVIAR does not report more than one distinctive credible set, we split this region into two adjacent regions and applied MaCAVIAR on these two subregions separately. MsCAVIAR selected the same 3-SNP 95% credible set (Figure S16) with index SNP being 10:80835998:C:T, and another 45-SNP credible set with index SNP being 10:80238015:C:T where 42 of them are replicated in the mJAM-Forward credible set. mJAM-SuSiE also identified the same 3-SNP credible set (95%) with the same index SNP 10:80835998:C:T but did not identify any credible set around 10:80238015:C:T. Instead, it reported two additional credible sets at 10:80260938:V1 (meta $p=2\times 10^{-10}$) and 10:80476778:V1 (meta $p=4\times 10^{-4}$) (Figure S16), and the credible set size is 2 and 5 respectively.

### 3.5 | Applied example 3: Secondary signal within 40 kb region of a leading SNP

**FIGURE 7:**
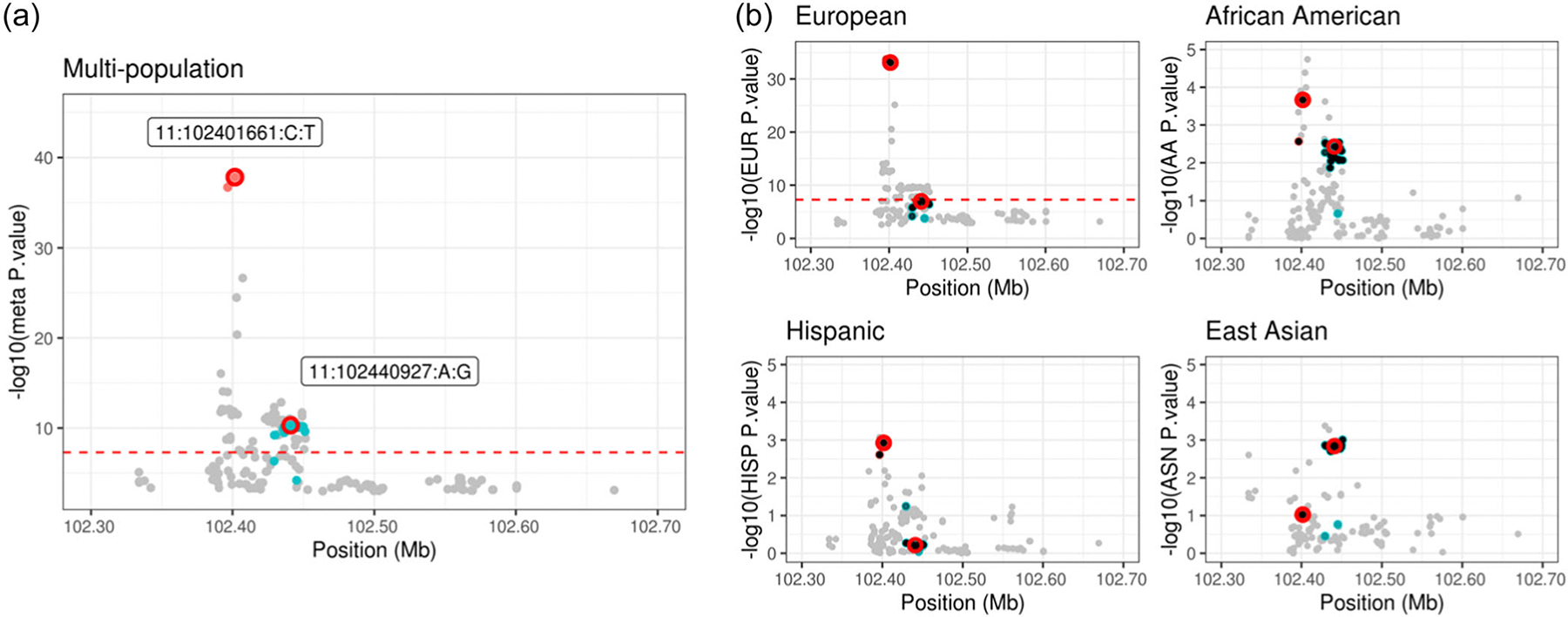
Manhattan plot for mJAM-Forward credible sets at chromosome 11 position 101601661 to 103201661. (a) *y*-axis is meta-analyzed −log10(*p*-value) from multipopulation analysis; index variants are labelled with GRCh37/hg19 reference assembly co-ordinates. (b) *y*-axis is −log10(*p*-value) from population-specific analysis.

The third applied example illustrates a scenario where there is a secondary signal within close proximity of the leading SNP in a chromosome 11 region. Many of the marginally significant variants in this region are upstream or intronic of the MMP7 and *MMP20* genes. *MMP7* and *MMP20* encode matrix metalloproteinases, a family that play a crucial role in the breakdown of extracellular matrix components, and are often upregulated in malignant tissues (Xu & Peng, 2015). This region spans 335.5 kb and consists of 191 SNPs. The population-specific LD structure and Manhattan plot of multi-population meta-analysis results are shown in Figure S17 and Figure 7. The lead variant, 11:102401661:C:T, has a multipopulation meta-analyzed *p*-value of 1. 5 × 10^−38^ and mJAM-Forward identified a secondary index SNP, 11:102440927:A:G, only 39 kb away which has a meta *p*-value of 4. 9 × 10^−11^. The $r^{2}$ between these two index SNPs is less than 0.01 in all four ancestry populations (Figure S17), suggesting statistical independence between these two SNPs. In each mJAM-Forward credible sets, the median $r^{2}$ between credible set SNPs and their corresponding index SNP is between 0.99 and 1 across the four ancestry groups (Table S3). COJO selected the same primary index SNP, 11:102401661:C:T, and a different secondary index, 11:102433309:A:G, which has a meta *p*-value of 1. 3 × 10^−7^ and is highly correlated with 11:102440927:A:G ($r^{2}=0.79$ in EUR; 0.55 in AA; 0.87 in LA and 0.99 in ASN). mJAM-SuSiE also selected two credible sets in this region: the first set has 2 SNPs which are both replicated in mJAM-Forward’s first credible set; the second set has 26 SNPs where 24 of them are found in mJAM-Forward’s second set. However, the index SNP of the second set in mJAM-SuSiE is one with lower marginal significance (meta $p=6.\;3\;\times 10^{-5}$) compared to mJAM-Forward.

Both mJAM-SuSiE and mJAM-Forward are able to identify multiple sets within one region without any predefined number of causal variants. On the other hand, the implementation of MsCAVIAR requires users to specify the maximum number of causal variants in a region to enumerate all possible causal configurations. Gauging the possible number of causal variants can be difficult when secondary signals are located close to the lead variant. In this example, the secondary signal is located only 39 kb away from the leading variant, and visual inspection of the Manhattan plot (Figure 7) suggests only one peak. Even if we specify the number of causal variants to be two when applying MsCAVIAR to this region, MsCAVIAR reports only one credible set such that the posterior probability of this set containing 2 causal variants is at least 0.95. Thus, it becomes difficult to separate the selected credible set SNPs into two distinctive groups. When the number of causal variants is set to two, MsCAVIAR selected 24 SNPs among which the 2 SNPs with highest posterior probability are 11:102401661:C:T and 11:102396607:C:T (Figure S18). However, these two SNPs are in high LD and thus are likely linked to a single underlying causal signal and not indicative of multiple independent signals.

## 4 | DISCUSSION

As integrating studies from ancestrally diverse populations may increase power to detect novel risk variants and improve fine-mapping resolution (Asimit et al., 2016; Mahajan et al., 2016, 2022), we extend our previous single-population fine-mapping through JAM to a multi-population approach, mJAM. mJAM requires only population-specific summary statistics and population-specific reference LD panels, which are more accessible than individual-level data to many researchers. mJAM explicitly incorporates the different LD structures across populations to yield conditional estimates of SNP effects from a single joint model. The mJAM framework can be used to first select index SNPs using existing feature selection approaches, such as forward stepwise selection, (Schaid et al., 2018) Bayesian model selection (Chen et al., 2015; Guan & Stephens, 2011; Zou et al., 2022), or regularized regression (Ayers & Cordell, 2010; Hastie, 2020). To demonstrate this flexibility, we have implemented mJAM through two implementations of feature selection: mJAM-SuSiE (a Bayesian approach) and mJAM-forward selection. We also combine the forward selection implementation with a second step to identify credible set SNPs. This step works given any set of index SNPs within a region by estimating a posterior credible set probability (PCSP) for a SNP defined as a combination of two component probabilities: one models the marginal association between the candidate SNP and the outcome; the other models the mediation effect of the index SNP on the candidate SNP, borrowing from a mediation framework. These PCSPs are then used to construct credible sets. PCSP can also be viewed as a weighted version of the model probability $Pr\left(M_{W}|\right.\;Data)$ where the weights are the mediation probability $Pr\left(\left|\tau_{W}-\tau_{W}^{\prime }\right|>|t||\tau_{W}=\tau_{W}^{\prime }\right)$. Assigning differential weights to the model probabilities can reduce credible set size by prioritizing SNPs with a higher mediation probability. More importantly, mediation probability associates potential credible set SNPs to a particular index SNP by fitting two-SNP or multi-SNP models illustrated in Figure 1b, as opposed to focusing on the posterior inclusion probability of each individual SNP and then forming a set of SNPs that collectively contain at least one or more causal SNP with high confidence. The closed-formed expression for PCSP allows computational efficient construction of credible sets, compared to other Bayesian approaches that often use computationally intensive algorithms to obtain posterior distributions (Table S4). It also allows credible set construction from any index SNP list allowing researchers to apply other feature selection methods or use existing lists or knowledge to determine index SNPs.

The two-stage model framework utilized in mJAM builds upon previous work highlighting the use of hierarchical JAM (hJAM) (Jiang et al., 2021), an approach for the joint analysis of marginal summary statistics that incorporates a prior information matrix. This matrix characterizes the SNPs and can include information such as SNP effects on gene expression analogous to TWAS or on intermediates biomarkers analogous to Mendelian randomization. mJAM is an extension to hJAM in that it replaces the prior information matrix in hJAM with a stacked identity matrix, $\left(\begin{array}{c} I_{P}\\ I_{P}\\ I_{P} \end{array}\right)$, as described in Methods section. The stacked identify matrix can be interpreted as our prior believe on the joint SNP effect estimates that all populations share the same true effect sizes.

In a set of realistic simulation settings, both mJAM implementations demonstrated the ability to infer the number of independent signals within a region, to differentiate signals from noise, and to achieve a sufficient level of sensitivity while preserving high fine-mapping resolution through small-sized credible sets. We also investigated the impact of imbalanced sample size across populations on model performance and demonstrated that all methods showed a similar decrease in terms of sensitivity and PPV when the sample size is imbalanced but the total sample size remains constant (Figures S6–8). mJAM is described using three populations in simulation studies and we apply mJAM to real data with four distinct populations. In practice, mJAM can be used to analyze a large number of studies or population-specific summary statistics facilitating flexibility in application. Thus, analyses do not need to be limited to aggregating continental ancestry populations, but can include numerous, more specific ancestry-appropriate reference panels to aggregate data across many studies (Figure S7). However, as with all summary statistic approaches that rely on reference panels, the ability to disentangle highly correlated SNPs will be driven by the sample sizes (Benner et al., 2017) and LD within and between the reference panels used (Peterson et al., 2019). In addition, another practical limitation to many summary statistics-based approaches is the requirement for complete summary statistics and reference data for all SNPs across all studies and populations analyzed (Jiang et al., 2018). Missingness can be due to the difference in genotyping arrays used by different studies, or rare variants not being captured due to limited sample size in certain studies. Filtering too many variants might be dangerous because as less information is used to disentangle the LD structure within each region and potentially missing the causal variant. An important feature of mJAM is that it will work even in the presence of differential missingness across studies or populations utilizing all information that is available.

In the simulation study with artificial LD structures, mJAM-SuSiE resulted in outstanding performance under high LD scenarios, achieving both high sensitivity and high PPV. However, as the significance of the causal variant(s) within a region increases, mJAM-SuSiE tends to break down selecting more false positive signals with each in separate credible sets. This results in a substantial decrease in the empirical coverage of mJAM-SuSiE credible sets. In practice, we recommend limiting the application of mJAM-SuSiE to only regions with SNPs with modest marginal statistically significance or to screen for any potential false positive credible sets before interpreting mJAM-SuSiE’s credible sets after estimation.

As with the original JAM for single-population fine-mapping (Newcombe et al., 2016), mJAM is developed under a continuous outcome model, but can be applied to binary-outcome summary statistics. In simulation studies, we showed that all comparative methods, including mJAM-Forward and mJAM-SuSiE, have similar performance when applied to linear-outcome summary statistics and binary-outcome summary statistics (in the form of log odds ratios and their standard errors) when the empirical power is similar. Transformation from logistic effect to linear effect under GWAS setting is also available (Pirinen et al., 2013), and is provided as an option in the R package.

We also carried out a case study of prostate cancer where mJAM is applied to the latest multi-population summary statistics, including samples of European, African, Hispanic, and East Asian ancestries. We chose three prostate cancer susceptible regions that have been either reported before or have shown some functional evidence. Through these three different regions with different characteristics in number of estimated independent signals and underlying LD within and between populations, we demonstrated the practical advantages of mJAM-Forward, including allowing more than one causal variant within a region, outputting individual credible sets corresponding to each index, and easily interpretable index variants with conditional estimates. In addition to the three applied examples shown here, mJAM has been applied to perform index variants selection across all regions in the latest multipopulation prostate cancer GWAS (Wang et al., 2023), leading to the identification of 187 novel variants associated with PCa. It is also worth noting that unlike simulation studies, the “true” variants in real data examples are unknown and real data examples are only for illustrating mJAM in practice, not for performance comparison between methods.

For all approaches that use marginal summary statistics and reference data, careful consideration and construction of the correlation matrices is important. This includes using a reference panel with ancestry and LD that matches the population in which the original marginal summary statistics were estimated (Benner et al., 2017; Li & Ritchie, 2021). Most methods also require that the correlation matrix used is full rank and positive-definite which is often driven by the sample size of the data and the frequency of the SNPs. For mJAM, such consideration must be considered across all populations used in the analysis. In mJAM-Forward, a LD pruning process is included to ensure stable estimates of the joint effect sizes; in both mJAM-Forward and mJAM-SuSiE, small positive constant will be to the diagonal of ${\left(G_{c}I_{c}\right)}^{\prime }G_{c}I_{c}$ to modify(“regularize”) the variance–covariance matrix if it cannot be inverted (Hormozdiari et al., 2014; Zou et al., 2022). Even so, for rare variants, mJAM estimates of multipopulation effect and standard errors that can be different from the marginal meta-analyzed estimates which use inverse-variance weighting. mJAM estimation from summary statistics assume Hardy-Weinberg equilibrium which some variants, especially rare variants, might not satisfy. In addition, many rare variants will also have large effect sizes and large standard errors from the population-specific summary statistics thus resulting in more uncertainty in multipopulation analysis compared to variants that are common across all populations. Secondly, in regions with extremely significant lead variants from a well-powered GWAS, even small degrees of LD can pull the marginal and conditional effect estimates of other variants away from the null. Thus, false positive signals might be selected if we apply the same threshold for index SNP selection and LD pruning. For such regions, researchers may consider setting a higher significance threshold for secondary signal selection and a more stringent LD threshold for pruning out correlated signals.

One limitation of mJAM is its key assumption that causal variants and their effect sizes are similar across all populations. mJAM is built upon a fixed-effect model framework because there exists evidence suggesting that common causal variants tend to have consistent effect sizes across populations (Marigorta & Navarro, 2013; Shi et al., 2020; Zanetti & Weale, 2018). The fixed-effect framework not only allows us to build from the fine-mapped results a multipopulation PRS model that can be applied to all individuals without estimating ancestry or collecting self-identified ancestry information, but also provides a practical advantage of an efficient model fitting process. As a sensitivity analysis of mis-specification of a fixed-effect model on truly heterogeneous populations, we demonstrate in extended simulation scenarios (Figure S19) that up to 50% variation in population-specific effect sizes does not change the credible set performance of all the methods substantially. Only in extreme cases when one population’s effect size is reduced to zero do all methods show significant performance decline through either poor sensitivity (mJAM-Forward and mJAM-SuSiE) or enlarged set sizes (MsCAVIAR). Though the current mJAM implementation cannot perform multipopulation fine-mapping and provide population-specific joint effect estimates simultaneously, mJAM is still able to provide an informative set of variants that contain the causal variant(s) with high probability when the model is mis-specified.

In conclusion, mJAM offers a flexible and efficient hierarchical modeling framework for multipopulation fine-mapping that first selects index variants and then constructs credible sets. We also proposed a novel definition of credible set probability based on the idea of mediation analysis to construct credible sets around any given index variants. In future research, we plan to relax the current mJAM assumption to allow for heterogeneous effect sizes across populations. For example, a direct extension can be to include an additional variance component in mJAM-Forward to capture the heterogeneity across population-specific effect sizes, similar to the between-study variance in a random-effect meta-analysis model. However, careful consideration of the underlying drivers of observed heterogeneity of effects needs to be considered in the context of fine-mapping and interpretation of results. In addition to possible heterogeneity resulting from gene-gene or gene-environment interaction, differential LD can lead to observed effect heterogeneity when noncausal variants have different LD across populations with a common underlying causal variant. In such instances, incorporation of the heterogeneity for inference could lead to spurious results or complications with interpretation (Han & Eskin, 2011; Morris, 2011; Yuan et al., 2023). Other potential future directions include follow-up functional analyses based on mJAM credible sets and polygenic risk score models based on mJAM fine-mapped results. mJAM is currently available as a R package for fine-mapping of specific regions and can easily be adapted for genome-wide applications.

## Supplementary Material

Supp Methods

Supp Figures Tables

## Figures and Tables

**TABLE 1:** Comparison of model performance on data simulated from real LD structure.

		Method				
		mJAM-Forward	mJAM-SuSiE	FE	COJO	MsCAVIAR

Credible set performance	Sensitivity^a^	0.930	0.910	0.972	-	0.994
	PPV^b^	0.064	0.069	0.024	-	0.022
	CS size^c^	19.70	18.37	48.88	-	56.62
	CS coverage^d^	0.934	0.940	-	-	1.000
Index SNP performance	Sensitivity^e^	0.218	0.174	-	0.186	0.134
	PPV^f^	0.219	0.021	-	0.144	0.134
	Number of selected index	1.00	0.97	-	1.51	1.00

Abbreviations: CS, credible set; FE, fixed-effect meta-analysis; LD, linkage disequilibrium; PPV, positive predictive value.

a Proportion of true causal SNPs being selected in a credible set, averaged over 500 simulations.

b Proportion of true causal SNPs over the total number of selected credible set SNPs, averaged over 500 simulations.

c Total number of SNPs included in a credible set, averaged over all 95% credible sets in 500 simulations.

d Proportion of 95% credible sets in 500 simulations that included at least one true causal SNP.

e Proportion of true causal SNPs being selected as an index SNP, averaged over 500 simulations.

f Proportion of true causal SNPs over the total number of selected index SNPs, averaged over 500 simulations.

## Data Availability

Data sharing is not applicable to this article as no new data were created or analyzed in this study. Both mJAM-Forward and mJAM-SuSiE are available as an R package at https://github.com/USCbiostats/hJAM/tree/master/R. The codes used for simulations and real data examples are available at https://github.com/USCbiostats/hJAM/tree/master/manuscript_codes.
