# Supplementary material for: Hierarchical joint analysis of marginal summary statistics—Part I: Multipopulation fine mapping and credible set construction
Source: Genet Epidemiol. Author manuscript; Available in PMC 2025 Apr 9. (PMC11980956; doi:10.1002/gepi.22562)
Supplement: Supp Methods [file NIHMS2066909-supplement-Supp_Methods.docx]

**Supplemental Material and Methods**

**Multi-population Joint Analysis of Marginal Summary Statistics (mJAM)**

To simplify notation and without loss of generality, we consider the scenario with three populations. Within each population, we can express the relationship between the genotype and the phenotype in a linear model as

|  | $\boldsymbol{y}^{\left( i \right)}=\boldsymbol{G}^{\left( i \right)}\boldsymbol{\beta}^{(i)}+\boldsymbol{\epsilon}^{(i)}$, for $i=1, 2, 3$ | ( 1 ) |
| --- | --- | --- |

where $\boldsymbol{y}^{\left( i \right)}$ is a $N^{\left( i \right)}\times1$ vector of mean-centered phenotypic trait value, with $N^{\left( i \right)}$ being the sample size of the $i^{th}$ population; $\boldsymbol{G}^{\left( \boldsymbol{i} \right)}$ is a $N^{\left( i \right)}\times p$ matrix of the individual-level genotype where each SNP has been centered to its mean; $\boldsymbol{\beta}^{(i)}$ is a $p \times1$ vector of the joint effect of the SNPs in the $i^{th}$ population; and $\boldsymbol{\epsilon}^{(i)} \sim N(0, \sigma^{2}I_{N^{\left( i \right)}})$ is the random error in the linear phenotypic model whereas $\sigma^{2}$ is the variance of the error term that is assumed to be the same across all populations.

Under a mixed-effect model, we can express the relationship between $\boldsymbol{\beta}^{(i)}$ and the pooled effect, $\boldsymbol{\beta}_{global}$, as the following

|  | $\left( \begin{matrix} \boldsymbol{\beta}^{(1)} \\ \boldsymbol{\beta}^{(2)} \\ \boldsymbol{\beta}^{(3)} \end{matrix} \right)=\left( \begin{matrix} \boldsymbol{I}_{P} \\ \boldsymbol{I}_{P} \\ \boldsymbol{I}_{P} \end{matrix} \right)\boldsymbol{\beta}_{\mathrm{global}}+\boldsymbol{\delta}$ | ( 2 ) |
| --- | --- | --- |

where $\boldsymbol{I}_{P}$ is the $p$ by $p$ identity matrix and $\boldsymbol{\delta}$ is a $3p\times1$ vector of random error that captures the deviation of population-specific effect $\boldsymbol{\beta}^{(i)}$ from the pooled effect $\boldsymbol{\beta}_{global}$, i.e., the between-population heterogeneity of the effect sizes. When $\boldsymbol{\delta}\boldsymbol{=0}$, Eq(2) is reduced to a fixed-effect model where the true SNP effects are the same across populations. In mJAM, we assume $\boldsymbol{\delta}\boldsymbol{=0}$ and adopt a fixed-effect approach given the reasons described in the main text. Under the fixed-effect model, Eq(1) becomes

|  | $\boldsymbol{y}^{\left( i \right)}=\boldsymbol{G}^{\left( i \right)}\boldsymbol{\beta}^{(i)}+\boldsymbol{\epsilon}^{(i)}=\boldsymbol{G}^{\left( i \right)}\left( \boldsymbol{I}_{P}\boldsymbol{\beta}_{\mathrm{global}}+\boldsymbol{\delta}^{\boldsymbol{'}} \right)+\boldsymbol{\epsilon}^{\left( i \right)}=\boldsymbol{G}^{\left( i \right)}\boldsymbol{I}_{P}\boldsymbol{\beta}_{\mathrm{global}}+\boldsymbol{\epsilon}^{(i)}$, for $i=1, 2, 3$ | ( 3 ) |
| --- | --- | --- |

where $\boldsymbol{\delta}^{\boldsymbol{'}}$ represents the same source of error as $\delta$ but with length $p$, and $\boldsymbol{\delta}^{\boldsymbol{'}}\boldsymbol{=0}$ by the fixed-effect model assumption. Eq (3) can also be written as

|  | $\left( \begin{matrix} \boldsymbol{y}^{(1)} \\ \boldsymbol{y}^{(2)} \\ \boldsymbol{y}^{(3)} \end{matrix} \right)=\left( \begin{matrix} \boldsymbol{G}^{(1)} & 0 & 0 \\ 0 & \boldsymbol{G}^{(2)} & 0 \\ 0 & 0 & \boldsymbol{G}^{(3)} \end{matrix} \right)\left( \begin{matrix} \boldsymbol{I}_{\boldsymbol{P}} \\ \boldsymbol{I}_{\boldsymbol{P}} \\ \boldsymbol{I}_{\boldsymbol{P}} \end{matrix} \right)\boldsymbol{\beta}_{global} + \boldsymbol{\epsilon}'$ | ( 4 ) |
| --- | --- | --- |

where $\boldsymbol{\epsilon}'\sim N(0, \sigma^{2}I_{N})$ and $N=\sum_{i} N^{(i)}$is the random error in the combined linear phenotypic model.

**mJAM likelihood with summary statistics**

Eq (4) presents the mJAM model when individual-level data is available. When only summary statistics is available, we can adapt Eq (4) and derive a likelihood model that only involves terms that can be estimated from summary statistics. Denote $\boldsymbol{G}_{c} := \left( \begin{matrix} \boldsymbol{G}^{(1)} & 0 & 0 \\ 0 & \boldsymbol{G}^{(2)} & 0 \\ 0 & 0 & \boldsymbol{G}^{(3)} \end{matrix} \right),$ $\boldsymbol{y}_{c}:=\left( \begin{matrix} \boldsymbol{y}^{(1)} \\ \boldsymbol{y}^{(2)} \\ \boldsymbol{y}^{(3)} \end{matrix} \right)$and $\boldsymbol{I}_{c}:=\left( \begin{matrix} \boldsymbol{I}_{\boldsymbol{P}} \\ \boldsymbol{I}_{\boldsymbol{P}} \\ \boldsymbol{I}_{\boldsymbol{P}} \end{matrix} \right)$. Using Eq (4) we can derive the distribution of $(\boldsymbol{G}_{c}\boldsymbol{I}_{c})'\boldsymbol{y}_{c}$ by multiplying the matrix $\boldsymbol{G}_{c}\boldsymbol{I}_{c}$ on every term, then we have:

|  | $(\boldsymbol{G}_{c}\boldsymbol{I}_{c})'\boldsymbol{y}_{c} \sim MVN\left( \left( {\left( \boldsymbol{G}_{c} \boldsymbol{I}_{c} \right)^{\boldsymbol{'}}\boldsymbol{G}}_{c} \boldsymbol{I}_{c} \right)\boldsymbol{\beta}_{global}, \sigma^{2}\left( {\left( \boldsymbol{G}_{c} \boldsymbol{I}_{c} \right)^{\boldsymbol{'}}\boldsymbol{G}}_{c} \boldsymbol{I}_{c} \right) \right)$ | ( 5 ) |
| --- | --- | --- |

And the maximum likelihood estimator of $\boldsymbol{\beta}_{global}$ can be written as

|  | ${\hat{\boldsymbol{\beta}}}_{global}=\left( {\left( \boldsymbol{G}_{c} \boldsymbol{I}_{c} \right)^{\boldsymbol{'}}\boldsymbol{G}}_{c} \boldsymbol{I}_{c} \right)^{-1}(\boldsymbol{G}_{c}\boldsymbol{I}_{c})'\boldsymbol{y}_{c}$ and var(${\hat{\boldsymbol{\beta}}}_{global}$) =$\sigma^{2}\left( {\left( \boldsymbol{G}_{c} \boldsymbol{I}_{c} \right)^{\boldsymbol{'}}\boldsymbol{G}}_{c} \boldsymbol{I}_{c} \right)^{-1}$ | ( 6 ) |
| --- | --- | --- |

where the estimation of $(\boldsymbol{G}_{c}\boldsymbol{I}_{c})'\boldsymbol{y}_{c}$, $\left( {\left( \boldsymbol{G}_{c} \boldsymbol{I}_{c} \right)^{\boldsymbol{'}}\boldsymbol{G}}_{c} \boldsymbol{I}_{c} \right)$ and also $\boldsymbol{y}_{c}^{T}\boldsymbol{y}_{c}$ from summary statistics are discussed in the later section “Estimation of sufficient statistics using summary data”.

**Fine mapping by mJAM sufficient statistics**

Zellner’s informative $g$-prior^1^ is a well adopted prior choice for variable selection in normal linear models because it is computationally efficient in evaluating the marginal likelihoods. $g$-prior is a conjugate Normal-Gamma prior for linear model $\boldsymbol{Y}=\boldsymbol{G\beta}+\boldsymbol{\epsilon}$ where

|  | $\boldsymbol{\beta}\vert\phi\sim N\left( \tilde{\boldsymbol{\beta}}, \frac{g}{\phi}\left( \boldsymbol{G}^{\boldsymbol{'}}\boldsymbol{G} \right)^{-1} \right), p\left( \phi\right)\propto\frac{1}{\phi}$ | ( 7 ) |
| --- | --- | --- |

and $\tilde{\boldsymbol{\beta}}$ is the prior mean effect, which is usually set to 0 in genetic models, and $\phi$ is the precision parameter (inverse of the variance of $\boldsymbol{\epsilon}$). $\tilde{\boldsymbol{\beta}}$ and $g$ are chosen by the users based on previous knowledge or belief. $g$ controls the amount of information in the prior relative to the observed data. A smaller $g$ means that one has a stronger belief in the prior information whereas as $g\to\infty$ the influence from the prior will vanish.

To perform fine-mapping analysis based on Eq(5), we impose the Zellner’s $g$-prior on $\boldsymbol{\beta}_{global}$:

|  | $\boldsymbol{\beta}_{global}\vert\sigma^{2}, \boldsymbol{G}_{c}, \boldsymbol{y}_{c}\sim N\left( \frac{g}{g+1}{\hat{\boldsymbol{\beta}}}_{global}, \frac{\sigma^{2}g}{g+1}\left( {\left( \boldsymbol{G}_{c} \boldsymbol{I}_{c} \right)^{\boldsymbol{'}}\boldsymbol{G}}_{c} \boldsymbol{I}_{c} \right)^{-1} \right)$ | ( 8 ) |
| --- | --- | --- |

|  | $\sigma^{2}\vert\boldsymbol{G}_{c}, \boldsymbol{y}_{c} \sim IG\left( \frac{n}{2}, \frac{s^{2}}{2}+\frac{1}{2(g+1)}\left( {\hat{\boldsymbol{\beta}}}_{global} \right)^{T}{\left( \boldsymbol{G}_{c} \boldsymbol{I}_{c} \right)^{\boldsymbol{'}}\boldsymbol{G}}_{c} \boldsymbol{I}_{c} {\hat{\boldsymbol{\beta}}}_{global} \right)$ | ( 9 ) |
| --- | --- | --- |

where $\sigma^{2}$ is the error variance in Eq (4), and $s^{2}= \left( \boldsymbol{y}_{c}- \boldsymbol{G}_{c}{\boldsymbol{I}_{c}\hat{\boldsymbol{\beta}}}_{global} \right)^{T}\left( \boldsymbol{y}_{c}- \boldsymbol{G}_{c}{\boldsymbol{I}_{c}\hat{\boldsymbol{\beta}}}_{global} \right)={(n-k-1)\hat{\sigma}}^{2}$ is the residual sum of squares, $\hat{\sigma}^{2}$ is then an unbiased estimate of $\sigma^{2}$. ${\hat{\boldsymbol{\beta}}}_{global}$ is the frequentist marginal likelihood estimate (MLE) of the joint SNP effect $\boldsymbol{\beta}_{global}$.

Then the marginal posterior distribution of $\boldsymbol{\beta}_{global}$ is:

|  | $\boldsymbol{\beta}_{global}\vert\boldsymbol{G}_{c}, \boldsymbol{y}_{c} \sim T_{p}\left( N,\frac{g}{g+1}{\hat{\boldsymbol{\beta}}}_{global}, \frac{g\left( s^{2}+\left( {\hat{\boldsymbol{\beta}}}_{global} \right)^{T}{\left( \boldsymbol{G}_{c} \boldsymbol{I}_{c} \right)^{\boldsymbol{'}}\boldsymbol{G}}_{c} \boldsymbol{I}_{c} {\hat{\boldsymbol{\beta}}}_{global}/(g+1) \right)}{n(g+1)}\left( {\left( \boldsymbol{G}_{c} \boldsymbol{I}_{c} \right)^{\boldsymbol{'}}\boldsymbol{G}}_{c} \boldsymbol{I}_{c} \right)^{-1} \right)$ | ( 10 ) |
| --- | --- | --- |

where $T_{p}$ denotes $p$-dimentional $T$ distribution with $N$ degrees of freedom, $N=\sum_{i} N^{(i)}$ is the total sample size of all population-specific studies combined. And the posterior mean and variance of $\boldsymbol{\beta}_{global}$ is:

|  | $E[\boldsymbol{\beta}_{global}\vert\boldsymbol{G}_{c}, \boldsymbol{y}_{c}]=\frac{g}{g+1}{\hat{\boldsymbol{\beta}}}_{global}$ | ( 11 ) |
| --- | --- | --- |

|  | $Var[\boldsymbol{\beta}_{global}\vert\boldsymbol{G}_{c}, \boldsymbol{y}_{c}]=\frac{g\left( s^{2}+\left( {\hat{\boldsymbol{\beta}}}_{global} \right)^{T}{\left( \boldsymbol{G}_{c} \boldsymbol{I}_{c} \right)^{\boldsymbol{'}}\boldsymbol{G}}_{c} \boldsymbol{I}_{c} {\hat{\boldsymbol{\beta}}}_{global}/(g+1) \right)}{n(g+1)}\left( {\left( \boldsymbol{G}_{c} \boldsymbol{I}_{c} \right)^{\boldsymbol{'}}\boldsymbol{G}}_{c} \boldsymbol{I}_{c} \right)^{-1}$ | ( 12 ) |
| --- | --- | --- |

Following Fernández et al.’s work^2^, we recommend setting $g=N=\sum_{i} N^{(i)}$ for model simplicity. When the total sample size is relatively large, such choice of $g$ will put more weight on the MLE of $\boldsymbol{\beta}_{global}$, ${\hat{\boldsymbol{\beta}}}_{global}$. As $N\to\infty$, the posterior mean of $\boldsymbol{\beta}_{global}$ will converge to ${\hat{\boldsymbol{\beta}}}_{global}$ and the posterior variance of $\boldsymbol{\beta}_{global}$ will converge to $var\left( {\hat{\boldsymbol{\beta}}}_{global} \right)= \sigma^{2}\left( {\left( \boldsymbol{G}_{c} \boldsymbol{I}_{c} \right)^{\boldsymbol{'}}\boldsymbol{G}}_{c} \boldsymbol{I}_{c} \right)^{-1}$.

The first step of mJAM-Forward (Algorithm 1 in main text) is to select index variants based on conditional significance of all variants in a region. This conditional significance is defined by the p-value under the above $g$ prior formulation by calculating a critical value (i.e. estimated effect size divided by its standard error) based on the $g$ prior posterior. To be more specific, based on Equation (10), this critical value should follow a standardized $T$ distribution which can be approximated by a standard normal distribution. Then a two-sided p-value can be calculated by comparing this critical value to a standard normal distribution. The critical value and the p-value calculated are identical to those from a traditional frequentist approach without the $g$ prior (since terms related to $g$ cancel when $g=n$). By adopting a $g$ prior on $\boldsymbol{\beta}_{global}$, the relative significance of all the variants in the region remain consistent with a frequentist approach without the $g$ prior, but the effect size estimation will be more robust as the posterior mean effect $E[\boldsymbol{\beta}_{global}| \boldsymbol{G}_{c}, \boldsymbol{y}_{c}]$ has a shrinkage factor $\frac{g}{g+1}$ preventing issues of overfitting.

**Estimation of sufficient statistics using summary data**

Based on the above expression of the posterior mean and variance of $\boldsymbol{\beta}_{global}$, it is straightforward that the posterior inference on $\boldsymbol{\beta}_{global}$ only depends on ${\left( \boldsymbol{G}_{c} \boldsymbol{I}_{c} \right)^{\boldsymbol{'}}\boldsymbol{G}}_{c} \boldsymbol{I}_{c}$, $\boldsymbol{G}_{c}\boldsymbol{I}_{c}\boldsymbol{y}_{c}$, and residual sum of squares $\boldsymbol{y}_{c}^{T}\boldsymbol{y}_{c}$. By expanding the matrices in each term, we can show that these statistics can be evaluated using just population-specific summary statistics:

- The $p\times p$ matrix, ${\boldsymbol{(}\boldsymbol{G}_{c} \boldsymbol{I}_{c}\boldsymbol{)'G}}_{c} \boldsymbol{I}_{c}=\boldsymbol{I}_{c}'\boldsymbol{G}_{c}'\boldsymbol{G}_{c}\boldsymbol{I}_{c}=\left( \begin{matrix} \boldsymbol{G}^{(1)}' & \boldsymbol{G}^{(2)}' & \boldsymbol{G}^{(3)}' \end{matrix} \right)\left( \begin{matrix} \boldsymbol{G}^{(1)} \\ \boldsymbol{G}^{(2)} \\ \boldsymbol{G}^{(3)} \end{matrix} \right) = \sum_{i=1}^{3} \boldsymbol{G}^{(i)}'\boldsymbol{G}^{(i)}$
- The p-vector, $\boldsymbol{(}\boldsymbol{G}_{c} \boldsymbol{I}_{c}\boldsymbol{)'}\left( \begin{matrix} \boldsymbol{y}^{(1)} \\ \boldsymbol{y}^{(2)} \\ \boldsymbol{y}^{(3)} \end{matrix} \right)=\left( \begin{matrix} \boldsymbol{G}^{(1)}' & \boldsymbol{G}^{(2)}' & \boldsymbol{G}^{(3)}' \end{matrix} \right)\left( \begin{matrix} \boldsymbol{y}^{(1)} \\ \boldsymbol{y}^{(2)} \\ \boldsymbol{y}^{(3)} \end{matrix} \right)= \sum_{i=1}^{3} \boldsymbol{G}^{(i)}'\boldsymbol{y}^{(i)}$
- The sum of squares, $\left( \begin{matrix} \boldsymbol{y}^{(1)}' & \boldsymbol{y}^{(2)}' & \boldsymbol{y}^{(3)}' \end{matrix} \right)\left( \begin{matrix} \boldsymbol{y}^{(1)} \\ \boldsymbol{y}^{(2)} \\ \boldsymbol{y}^{(3)} \end{matrix} \right)=\sum_{i=1}^{3} \boldsymbol{y}^{(i)}'\boldsymbol{y}^{(i)}$
- The sample size of each GWAS, $N^{\left( 1 \right)}, N^{\left( 2 \right)}, N^{\left( 3 \right)}$

Thus, we can estimate $\boldsymbol{G}^{\boldsymbol{(i)}}\boldsymbol{'}\boldsymbol{G}^{\boldsymbol{(i)}}$**,** $\boldsymbol{G}^{(\boldsymbol{i})}\boldsymbol{'}\boldsymbol{y}^{(\boldsymbol{i})}$, $\boldsymbol{y}^{\boldsymbol{(i)}}\boldsymbol{'}\boldsymbol{y}^{\boldsymbol{(i)}}$ for each population individually and then sum up the population-specific statistics to obtain the mJAM summary statistics. To simply notions, we will drop the superscripts and just use $\boldsymbol{G'G}, \boldsymbol{G'y}, \boldsymbol{y'y}$ and $N$ in the following discussion.

**Estimation of**$\boldsymbol{G'G}$

Following Yang *et al*.’s work^3^, the variance-covariance matrix of the GWAS samples,$\boldsymbol{G'G}$, can be estimated from the LD structure from a reference panel, and effect allele frequencies from the GWAS summary statistics data.

Let $\boldsymbol{W}$ = {$w_{ij}$} be the genotype matrix of the reference panel with sample size $m$, and $w_{ij}$ denotes the genotype for the $j^{th}$ SNP for the $i^{th}$ individual in the reference panel. Standardize $\boldsymbol{W}$ by letting $w_{ij}=-2f_{i},1-2f_{i},2-2f_{i}$, where $f_{i}$ is the allele frequency of SNP $i$ in the reference data. Due to the difference in sample sizes of the reference data and the GWAS data, we cannot use $\boldsymbol{W}’\boldsymbol{W}$ to estimate $\boldsymbol{G'G}$ directly. To adjust for such difference, define

|  | $\boldsymbol{B}=\boldsymbol{D}^{1/2}\boldsymbol{D}_{\boldsymbol{w}}^{-1/2}\boldsymbol{W' W} \boldsymbol{D}_{\boldsymbol{w}}^{-1/2} \boldsymbol{D}^{1/2}$ | ( 13 ) |
| --- | --- | --- |

where $\boldsymbol{D}=\{D_{j}\}$ is the diagonal matrix of $\boldsymbol{G'G}$, with $D_{j}=\sum_{i=1}^{N} g_{ij}^{2}$ and $g_{ij}$ being the mean-centered genotype for the $j^{th}$ SNP for the $i^{th}$ individual in the discovery GWAS sample. Similarly, let $\boldsymbol{D}_{\boldsymbol{W}}$ be the diagonal matrix of $\boldsymbol{W}’\boldsymbol{W}$, with $D_{W\left( j \right)}=\sum_{i=1}^{m} w_{ij}^{2}$. $\boldsymbol{B}$ is then an estimate for the variance-covariance matrix of the GWAS samples $\boldsymbol{G'G}$, adjusted for the sample size in the GWAS sample. Since we only have summary-level data for the GWAS sample and thus $D_{j}=\sum_{i=1}^{N} g_{ij}^{2}$ is unknown, we use $D_{j}=2p_{j}\left( 1-p_{j} \right)N$ to estimate $\sum_{i=1}^{N} g_{ij}^{2}$ under the Hardy–Weinberg Equilibrium (HWE), where $p_{j}$ is the allele frequency of SNP $j$ in the GWAS sample. More detailed derivation can be found in the Online Methods section in Yang *et al*.’s work^3^.

**Estimation of** $\boldsymbol{G'y}$

The total trait burden of each SNP, $\boldsymbol{G'y}$**,** can be estimated from effect allele frequencies and marginal SNP effect estimates when HWE and an additive genetic model is assumed^4^. Define $\boldsymbol{z}:=\boldsymbol{G'y}$. To estimate $\boldsymbol{z}$ from summary statistics data, the first step is to construct estimates of the genotype counts $n_{mg}$ for SNP $m$ where $g$ takes value of 0,1,2. The subsequent step is estimate the trait mean within each genotype group, denoted as $\bar{y}_{j0}$, $\bar{y}_{j1}$, $\bar{y}_{j2}$, as the following

|  | ${\hat{\bar{y}}}_{m0} = -\frac{\hat{n}_{m1}\hat{\beta}_{j}+2\hat{n}_{m2}\hat{\beta}_{m}}{N}$, ${\hat{\bar{y}}}_{m1}={\hat{\bar{y}}}_{m0}+\hat{\beta}_{m},$ and ${\hat{\bar{y}}}_{m2}={\hat{\bar{y}}}_{m0}+2\hat{\beta}_{m}$ | ( 14 ) |
| --- | --- | --- |

where $\hat{\beta}_{m}$ is the marginal additive SNP effect estimate for SNP $m$ from the GWAS data, and $N$ is the GWAS sample size. The genotype counts $n_{mg}$ is estimated as the following assuming HWE

|  | $\hat{n}_{j0}=\left( 1-\hat{p}_{j} \right)^{2}N$,  $\hat{n}_{j1}=2 \hat{p}_{j}\left( 1-\hat{p}_{j} \right)N$, and  $\hat{n}_{j2}=\hat{p}_{j}^{2}N$ | ( 15 ) |
| --- | --- | --- |

For SNP $m$, the total trait burden $z_{m}$

|  | $z_{m} = \sum_{i} y_{i}\times G_{m,i}^{'}=\bar{y}_{m1}n_{m1}+2\bar{y}_{m2}n_{m2}$ | ( 16 ) |
| --- | --- | --- |

where $G_{m,i}^{'}$ is the genotype matrix entry corresponding to individual $i$’s genotype for SNP $m$.

By plugging the estimates in Eq(14) and Eq(15) into Eq(16), we can obtain the estimate for the total trait burden $z_{m}$ for SNP $m$.

**Estimation of**$\boldsymbol{y'y}$

In the association analysis of a single SNP $j$, the residual variance $\boldsymbol{y'y}$ in the $i^{th}$ population can be estimated as

|  | ${\boldsymbol{y'y} \mathbf{=}D}_{j}S_{j}^{2}\left( N-1 \right)+D_{j}\hat{\beta}_{j}^{2}$ | ( 17 ) |
| --- | --- | --- |

where $\hat{\beta}_{j}$ is the marginal effect estimate for SNP $j$ and $S_{j}^{2}$ is the squared standard error of $\hat{\beta}_{j}$.^1^ Yang *et al.* suggested to take the median of $D_{j}S_{j}^{2}\left( N-1 \right)+D_{j}\hat{\beta}_{j}^{2}$ across all SNPs to get a single estimate of $\boldsymbol{y'y}$ for the $i^{th}$ population. However, for rare SNPs whose estimates that have relatively large standard errors, the median estimate of $\boldsymbol{y'y}$ tends to underestimate the residual variance in the one-SNP models. Thus, for all one-SNP models in mJAM, we propose a modified estimate of $\boldsymbol{y'y}$ that takes a weighted average between SNP-specific estimates and the median across all SNPs.

|  | $\boldsymbol{y}^{\boldsymbol{'}}\boldsymbol{y =}w_{j}\boldsymbol{\cdot}{\boldsymbol{y}^{\boldsymbol{'}}\boldsymbol{y}}_{\boldsymbol{j}}\boldsymbol{+}{(1-w}_{j})\boldsymbol{\cdot}{\boldsymbol{y}^{\boldsymbol{'}}\boldsymbol{y}}_{\boldsymbol{m}}$ | ( 18 ) |
| --- | --- | --- |

where ${\boldsymbol{y}^{\boldsymbol{'}}\boldsymbol{y}}_{\boldsymbol{j}}\boldsymbol{=}D_{j}S_{j}^{2}\left( N-1 \right)+D_{j}\hat{\beta}_{j}^{2}$ is the SNP-specific residual variance estimate for SNP $j$, ${\boldsymbol{y}^{\boldsymbol{'}}\boldsymbol{y}}_{\boldsymbol{m}}$ is the median across all ${\boldsymbol{y}^{\boldsymbol{'}}\boldsymbol{y}}_{\boldsymbol{j}}$, $w_{j}=\frac{{\boldsymbol{y}^{\boldsymbol{'}}\boldsymbol{y}}_{\boldsymbol{m}}}{{\boldsymbol{y}^{\boldsymbol{'}}\boldsymbol{y}}_{\boldsymbol{m}}\boldsymbol{+|}{\boldsymbol{y}^{\boldsymbol{'}}\boldsymbol{y}}_{\boldsymbol{m}}\boldsymbol{-}{\boldsymbol{y}^{\boldsymbol{'}}\boldsymbol{y}}_{\boldsymbol{j}}\boldsymbol{|}}$ is the relative weight of median $\boldsymbol{y'y}$ to SNP-specific $\boldsymbol{y'y}$.

**Posterior Model Probability**

To quantify how significant is a potential credible set SNP associated with the outcome, $\boldsymbol{Y,}$ we adopted a Bayesian posterior probability of models built upon Bayes factors for pairs of hypotheses.^1^ For a putative credible set SNP $j$, the posterior probability of the one-SNP model of SNP $j$, denoted as $M_{j}$, can be expressed as

|  | $\Pr\left( M_{j} \right\vert Data)=\frac{p(M_{j})BF\left[ M_{j}:M_{Null} \right]}{\sum_{j} p(M_{j})BF\left[ M_{j}:M_{Null} \right]}$ | ( 19 ) |
| --- | --- | --- |

where $p(M_{j})$ is the prior probability of model $M_{j},$and $BF\left[ M_{j}:M_{Null} \right]$ is the Bayes factor for comparing $M_{j}$ to $M_{Null}$, the null model. $BF\left[ M_{j}:M_{Null} \right]$ is simply the ratio of marginal likelihood of the data under model $M_{j}$ versus that under the null model:

|  | $BF\left[ M_{j}:M_{Null} \right]=\frac{P(\boldsymbol{Y}\vert M_{j})}{P(\boldsymbol{Y}\vert M_{Null})}$ | ( 20 ) |
| --- | --- | --- |

We further adopt Zellner’s $g$ prior in the derivation of $BF\left[ M_{j}:M_{Null} \right]$. It has been shown that the Bayes factor under $g$ prior formulation comparing $M_{j}$ to the null model, $BF\left[ M_{j}:M_{Null} \right]$, can be written as

|  | $BF\left[ M_{j}:M_{Null} \right]= \left[ \frac{1+g}{1+g(1-R_{j}^{2})} \right]^{\frac{(n-1)}{2}}\left( 1+g \right)^{\frac{-p_{j}}{2}}$ | ( 21 ) |
| --- | --- | --- |

where $R_{j}^{2}$ is the coefficient of determination of regression model $M_{j}$, $n$ is the sample size in $M_{j}$, and $p_{j}$ is the number of coefficients in $M_{j}$.^1^

With only summary statistics, the coefficient of determination $R_{j}^{2}$ can be expressed as

|  | $R_{j}^{2}=\frac{{\hat{\boldsymbol{b}}}^{\boldsymbol{'}}\boldsymbol{G}^{\boldsymbol{'}}\boldsymbol{y}}{\boldsymbol{y}^{\boldsymbol{'}}\boldsymbol{y}}=\frac{\left( \boldsymbol{G}^{\boldsymbol{'}}\boldsymbol{y} \right)^{'}\left( \boldsymbol{G}^{'}\boldsymbol{G} \right)^{-1}\boldsymbol{G}^{\boldsymbol{'}}\boldsymbol{y}}{\boldsymbol{y}^{\boldsymbol{'}}\boldsymbol{y}}$ $=\frac{\hat{\boldsymbol{b}}\boldsymbol{'D}\hat{\boldsymbol{\beta}}}{\boldsymbol{y'y}}=\frac{(\boldsymbol{D}\hat{\boldsymbol{\beta}})'{(\boldsymbol{G}'\boldsymbol{G})}^{-1}\boldsymbol{D}\hat{\boldsymbol{\beta}}}{\boldsymbol{y'y}}$ | ( 22 ) |
| --- | --- | --- |

where $\boldsymbol{G}'\boldsymbol{G}$ is variance-covariance matrix of SNPs within $M_{j}$, $\boldsymbol{D}$ is the diagonal matrix of $\boldsymbol{G'G}$**,** and $\hat{\boldsymbol{\beta}}$ is the vector of marginal effect estimates of SNPs within $M_{j}$. All these components can be estimated using the marginal summary statistics with the approach discussed in previous sections.

To build the credible sets for index SNPs selected conditional on the previously selected index SNP(s) in the region, we adjust the posterior model probabilities of putative credible set SNPs to also be conditional on the presence of any previous index SNP(s). Then for a putative credible set SNP $j$, model $M_{j}$ is now a multi-SNP model with SNP $j$ and all previous index SNP(s). We then replace $R_{j}^{2}$ with partial R^2^ of SNP $j$ conditional on all previous index SNP(s) to calculate $BF\left[ M_{j}:M_{Null} \right]$, that is,

|  | $R_{\gamma_{2}\vert\gamma_{1}}^{2}=\frac{SSR(\gamma_{2})-SSR(\gamma_{1})}{SSTO-SSR(\gamma_{1})}=\frac{{{\boldsymbol{(}\hat{\boldsymbol{b}}\boldsymbol{'D}\hat{\boldsymbol{\beta}}\boldsymbol{)}}_{\gamma_{2}}\boldsymbol{-(}\hat{\boldsymbol{b}}\boldsymbol{'D}\hat{\boldsymbol{\beta}}\boldsymbol{)}}_{\gamma_{1}}}{\boldsymbol{y}^{\boldsymbol{'}}\boldsymbol{y-}{\boldsymbol{(}\hat{\boldsymbol{b}}\boldsymbol{'D}\hat{\boldsymbol{\beta}}\boldsymbol{)}}_{\gamma_{1}}}$ | ( 23 ) |
| --- | --- | --- |

where $\gamma_{1}$is the model with all previous index SNP(s), $\gamma_{2}$ is the model with SNP $j$ and all previous index SNP(s). Note that $\gamma_{1}$is nested within $\gamma_{2}$. With each model ($\gamma_{1}$ or $\gamma_{2}$), $\boldsymbol{D}$ is the diagonal matrix of $\boldsymbol{G'G}$**,** and $\hat{\boldsymbol{\beta}}$ is the vector of marginal effect estimates of SNPs within $\gamma_{1}$ or $\gamma_{2}$. $\hat{\boldsymbol{b}}$ refers to the joint effect estimates which can be obtained using the approach discussed in previous sections.

**Posterior Mediation Probability**

As shown in Figure 1 in the main text, the mediation effect of an index SNP, $X$, on the relationship between a candidate credible set SNP, $W$, and the outcome, $Y$, can be evaluated through the difference in the total effect and the indirect effect, i.e. $\left| \tau_{W} -\tau_{W}^{'} \right|$, where $\tau_{W}$ is the total effect (the marginal effect of $W$ on $Y$) and $\tau_{W}^{'}$ is the direct effect (the adjusted effect of $W$ on $Y$ adjusted for $X$). Suppose the observed difference between the total effect and the direct effect is denoted as $t$. Then the mediation probability is expressed as

|  | $\Pr(Mediation\vert Data)=\Pr\left( \left\vert\tau_{W} -\tau_{W}^{'} \right\vert>\left\vert t \right\vert\right\vert\tau_{W} =\tau_{W}^{'})$ | ( 24 ) |
| --- | --- | --- |

We again adopt Zellner’s $g$ prior formulation and obtain a Wald-type statistics for $\left| \tau_{W} -\tau_{W}^{'} \right|.$ To be more specific, in the first round of index SNP selection in mJAM-Forward, we first evaluate the posterior mean effect and variance for $\tau_{W}$ by fitting a one-SNP model of only $W$ following Eq(10)-(12). Secondly, the posterior mean effect and variance for $\tau_{W}^{'}$ can be obtained by fitting a two-SNP model of $W$ and $X$ jointly under Eq(10)-(12) and then retrieving the effect of $W$ in the joint estimate. In subsequent rounds of index SNP selection, we can continue to fit two separate multi-SNP models, one with $W$ and previous index SNP(s) and the other with $W$,$X$ and previous index SNP(s), to obtain the posterior effect of $\tau_{W}$ and $\tau_{W}^{'}$ respectively.

Assume the posterior distributions of $\tau_{W}$ and $\tau_{W}^{'}$ are independent and denote the posterior mean of $\tau_{W}$ is $\tau_{a}$ and the variance in the posterior distribution of $\tau$ is $v(\tau_{a})$. Similarly, for $\tau_{W}^{'}$, the posterior mean is $\tau_{a}^{'}$ and the variance is $v(\tau_{a}^{'} )$. Then the Wald-type statistic for $\left| \tau_{W} -\tau_{W}^{'} \right|$ is

|  | $Z_{\tau}:=\frac{\left\vert\tau_{a} -\tau_{a}^{'} \right\vert}{\sqrt{v\left( \tau_{a} \right)+v(\tau_{a}^{'} )}}$ | ( 25 ) |
| --- | --- | --- |

where $\tau_{a}$, $\tau_{a}^{'}$, $v\left( \tau_{a} \right)$, and $v(\tau_{a}^{'} )$ estimated via the closed form approximate multivariate Gaussian distribution shown in Eq (6) to Eq (12). Then $Z_{\tau}^{2}$ approximately follows a Chi-squared distribution with 1 degree of freedom under the null hypothesis that $\tau_{W} =\tau_{W}^{'}$.

### Multi-population Fine Mapping Using Sum of Single Effect Model (mJAM-SuSiE)

To allow for multiple non-zero effects in the common joint effect $\boldsymbol{\beta}_{\mathrm{global}}$, the Sum of Single Effect Model (SuSiE)^5^ proposed a new approach to model the sparse vector of $\boldsymbol{\beta}_{\mathrm{global}}$ as a sum of “single-effect” vectors, each with one non-zero effect. Taken together the multi-population joint analysis setting (equation (3) and (4) in main text) and SuSiE, fine-mapping in terms of the joint effect $\boldsymbol{\beta}_{\mathrm{global}}$ can be expressed as

|  | $\boldsymbol{\beta}_{\mathrm{global}}=\sum_{l=1}^{L} \boldsymbol{\beta}_{\boldsymbol{l}}=\sum_{l=1}^{L} \beta_{l}\boldsymbol{\gamma}_{\boldsymbol{l}}$ | ( 26 ) |
| --- | --- | --- |
|  | $\boldsymbol{\gamma}_{\boldsymbol{l}}\sim\mathrm{Mult}\left( 1, \tau\right) \mathrm{and} \beta_{l}\sim N_{1}\left( 0, \sigma_{0l}^{2} \right),$ | ( 27 ) |

where $l$ is the index of credible sets, and $L$ denotes the largest number of credible sets allowed in fitting, and $\sigma_{0l}^{2}$ denotes the prior variance of the non-zero effect $\beta_{l}$. SuSiE is robust to overstating $L$; thus, in practice we recommend setting $L$ larger than the number of potential causal signals in a region.

The susieR package^6^ provides the implementation of SuSiE not only with inputs as individual-level data, but also with inputs as sufficient statistics (the statistics are sufficient for estimating the regression coefficients under the SuSiE model). When only summary data is available, we employ ${\boldsymbol{(}\boldsymbol{G}_{c} \boldsymbol{I}_{c}\boldsymbol{)'G}}_{c} \boldsymbol{I}_{c}$, $\boldsymbol{(}\boldsymbol{G}_{c} \boldsymbol{I}_{c}\boldsymbol{)'}\boldsymbol{y}_{c}$, and $\boldsymbol{y}_{c}'\boldsymbol{y}_{c}$, which can be estimated from population-specific marginal effect estimates and reference individual-level dosage data for each population, to obtain the posterior mean and posterior inclusion probability (PIP) of each SNP using the summary statistic version of SuSiE. See Supplemental Algorithm 1 below for fitting mJAM-SuSiE model.

Supplemental Algorithm 1 Pseudo algorithm for fitting mJAM-SuSiE and constructing credible sets using SuSiE PIP, modified from Wang *et al*. (2020).

| Input data:  ${\hat{\boldsymbol{\beta}}}^{(i)}$, $se\left( {\hat{\boldsymbol{\beta}}}^{(i)} \right),$ $N_{GWAS},$ $\mathbf{EA}\mathbf{F}^{\mathbf{(i)}},$ $\boldsymbol{G}_{\boldsymbol{R}}^{\boldsymbol{(i)}}$ for each study indexed by $i$  Input arguments: $L$, the maximum number of credible sets allowed; $r$, the minimum absolute correlation allowed in a credible set; requested coverage.  Function required:  (1) $susie\_suff\_stat(\boldsymbol{X}^{\boldsymbol{'}}\boldsymbol{X}, \boldsymbol{X}^{\boldsymbol{'}}\boldsymbol{y}; L) \to\boldsymbol{PIP}$ that computes a $p$ by $L$posterior probability matrix using SuSiE with sufficient statistics.   1. Compute mJAM statistics ${\boldsymbol{(}\boldsymbol{G}_{c} \boldsymbol{I}_{c}\boldsymbol{)'G}}_{c} \boldsymbol{I}_{c}$, $\boldsymbol{(}\boldsymbol{G}_{c} \boldsymbol{I}_{c}\boldsymbol{)'}\boldsymbol{y}_{c}$, and $\boldsymbol{y}_{c}'\boldsymbol{y}_{c}$ using ${\hat{\boldsymbol{\beta}}}^{(i)}$, $se\left( {\hat{\boldsymbol{\beta}}}^{(i)} \right),$ $N_{GWAS},$ $\mathbf{EA}\mathbf{F}^{\mathbf{(i)}},$ $\boldsymbol{G}_{\boldsymbol{R}}^{\boldsymbol{(i)}}$ 2. Fit $susie\_suff\_stat({\boldsymbol{(}\boldsymbol{G}_{c} \boldsymbol{I}_{c}\boldsymbol{)'G}}_{c} \boldsymbol{I}_{c}, \boldsymbol{(}\boldsymbol{G}_{c} \boldsymbol{I}_{c}\boldsymbol{)'}\boldsymbol{y}_{c}; L) \to\boldsymbol{PIP}$ 3. For $l$ in 1,…, $L$ do 4. Find a set of SNPs where cumulative $\boldsymbol{PIP}^{(l)}$ reaches the requested coverage and all pairwise absolute correlation are no less than $r$. If not, report no credible set.   Return credible set(s) with index SNP(s) and PIP. |
| --- |

**Incorporating missing variants in mJAM**

In genetic association studies with more than one cohort or study, it is common that a particular SNP might be available in some studies but missing in the others. A notable practical feature of the mJAM framework is that it allows for these SNPs with missing information to be analyzed without being filtered or removed. This is accomplished with a simple modification by substituting a value of zero in the identity matrix in Equation ( 3 ) and ( 4 ) in the main text. Such modification then allows for observed statistics from other populations to be used but removes the contribution from the population in which it is missing but does not alter the algorithm nor the fitting process. This modification is applicable either when the SNP is missing in the reference panel or when the population-specific GWAS summary statistics are not available for the SNP.

Suppose SNP $m$ is missing in population $k$. To analyze all available data without filtering this SNP, we can find the identity matrix of population $k$ in the mJAM model, then replace the diagonal term of the corresponding SNP with 0. Now the identify matrix is as:

$$\left( \begin{matrix} \boldsymbol{I}_{p}^{(1)} & 0 & 0 & 0 & 0 \\ 0 & \ldots& 0 & 0 & 0 \\ 0 & 0 & \boldsymbol{I}_{p}^{(k)} & 0 & 0 \\ 0 & 0 & 0 & \ldots& 0 \\ 0 & 0 & 0 & 0 & \boldsymbol{I}_{p}^{(K)} \end{matrix} \right)$$

and $\boldsymbol{I}_{p}^{(k)}=\left( \begin{matrix} 1 & & & & & \\ & 1 & & & & \\ & & \ddots& & & \\ & & & 0 & & \\ & & & & \ddots& \\ & & & & & 1 \end{matrix} \right)$ where the $m^{th}$ term on the diagonal is replaced by 0.

For SNP $m$, the resulting mJAM statistics become the following:

|  | $\sum_{i=1}^{K} {\boldsymbol{G}_{m}^{\left( i \right)}}^{'}\boldsymbol{y}_{m}^{(i)}={\boldsymbol{G}_{m}^{\left( 1 \right)}}^{'}\boldsymbol{y}_{m}^{(1)}+\ldots+{\boldsymbol{G}_{m}^{\left( k-1 \right)}}^{'}\boldsymbol{y}_{m}^{\left( k-1 \right)}+{\boldsymbol{G}_{m}^{\left( k+1 \right)}}^{'}\boldsymbol{y}_{m}^{\left( k+1 \right)}+\ldots+{\boldsymbol{G}_{m}^{\left( K \right)}}^{'}\boldsymbol{y}_{m}^{(K)}$ | ( 28 ) |
| --- | --- | --- |
|  | $\sum_{i=1}^{K} {\boldsymbol{G}_{m}^{\left( i \right)}}^{'}\boldsymbol{G}_{m}^{(i)}={\boldsymbol{G}_{m}^{\left( 1 \right)}}^{'}\boldsymbol{G}_{m}^{(1)}+\ldots+{\boldsymbol{G}_{m}^{\left( k-1 \right)}}^{'}\boldsymbol{G}_{m}^{\left( k-1 \right)}+{\boldsymbol{G}_{m}^{\left( k+1 \right)}}^{'}\boldsymbol{G}_{m}^{\left( k+1 \right)}+\ldots+{\boldsymbol{G}_{m}^{\left( K \right)}}^{'}\boldsymbol{G}_{m}^{(K)}$ | ( 29 ) |

**Overview of Simulation Studies**

We conducted simulation studies to compare the performance of mJAM-SuSiE, mJAM-Forward, with three commonly used alternative approaches: fixed-effect meta-analysis, COJO conditional stepwise selection and MsCAVIAR. Fixed-effect meta-analysis takes an inverse-variance weighted average of the marginal estimates from individual studies or populations. COJO approximates the conditional and joint effect from summary statistics and single reference LD and then implements a stepwise selection based on conditional *P*-values. Additionally, for use of COJO on multiple populations, the summary-level statistics come from the fixed-effect meta-analysis across all populations and the reference LD can be obtained from either the pooled individual-level genotype data or a subset of the meta-analysis sample. We used the former as the reference LD for COJO in our simulations. MsCAVIAR is built upon a Bayesian multivariate normal framework first described as CAVIAR^7^ and it accounts for between-study or between-population heterogeneity using a random-effects model.

The credible level for mJAM-SuSiE, mJAM-Forward and MsCAVIAR is set as 95%. MsCAVIAR uses binary vector $C= {\{0,1\}}^{p}$ to indicate whether a SNP truly has a non-zero effect where $p$ is the number of SNPs in a region. Depending on the maximum number of true causal SNPs specified in MsCAVIAR, the number of possibilities of this binary vector $C= {\{0,1\}}^{p}$ (i.e., the number of possible model configurations) will vary. MsCAVIAR will compute the posterior inclusion probability for every SNP under every model configuration and thus the maximum number of causal variants is a key parameter that not only affects how many variants are potentially identified but also greatly impacts computational time. In our simulation studies, we set the maximum number of causal SNPs to the number of true causal SNPs in each simulation (a best case situation). In mJAM-Forward, the LD threshold for index SNP selection is r^2^ $\geq$0.5 within populations (meaning SNPs whose r^2^ with the newly selected index SNP exceed 0.5 in *any* population will be pruned) and r^2^ $\geq$0.2 across populations (meaning SNPs whose r^2^ with the newly selected index SNP exceed 0.2 in *all* populations will be pruned). In simulation studies, since the total number of variants is much smaller than real GWAS, the conditional P-value cutoff in mJAM-Forward is set to the Bonferroni-corrected P-value, instead of $5\times{10}^{-8}$. In mJAM-SuSiE, we use SuSiE’s default value of minimum absolute correlation (r = 0.5, equivalent to r^2^ = 0.25) for constructing mJAM-SuSiE’s credible sets, which is a reasonable threshold since the pairwise correlation we set in the moderate and high LD scenarios is 0.6 and 0.9 respectively. We also use the SuSiE’s default value of the maximum number of non-zero effects (L = 10), which is also recommended by the SuSiE authors. In our simulation, the maximum number of causal variants is 3 across all scenarios so we believe L = 10 will provide a large enough cap for SuSiE. The conditional P-value cutoff in mJAM-Forward and COJO is set as the Bonferroni-corrected P-value. In FE, we also use a Bonferroni-corrected P-value cutoff for its marginal P-values. All other method-specific parameters follow the default settings suggested in each program.

We performed two sets of scenarios: 1) simulated correlation structures with the same block LD structures across populations; and 2) simulated correlation based on real genetic correlation structures observed in the study cohort from Elucidating Loci Involved in Prostate Cancer Susceptibility (ELLIPSE) OncoArray Consortium^8^. Each simulation scenario was repeated 500 times.

**Set up of Simulation Study on Structured LD**

For the first set of scenarios, we first simulated a baseline scenario where each population has 3 individual association studies with N = 5,000 each to closely represent the real-life situation where there are multiple association studies performed for each ethnic group (total sample size = 5,000 × 3 studies/population × 3 population = 45,000). A total of 50 SNPs are simulated in 5 blocks of 10 SNPs. Within each block of 10 SNPs, the pair-wise correlations are uniformly set to a constant value r^2^ across ancestries for simplicity. r^2^ varies from 0, 0.6^2^ and 0.9^2^ to represent independent, moderate LD and high LD scenarios. Corresponding LD heatmaps are shown in Figure S1. The effect allele frequency is set as 0.2, 0.4 and 0.6 for each population respectively. For the baseline scenario, we then selected a single SNP to be the causal variant and generated a continuous outcome from an additive phenotypic model following Equation (1) in the main text. The corresponding variance of the error term in Equation (1) is set as 1. Under such setting, we chose to set effect size of the causal variant to 0.03 because such an effect size provided 80.6% empirical power (across 500 simulations under the baseline scenario) of identifying the causal variants through fixed-effect meta-analysis as well as a heritability value of around 0.03%. Then marginal summary statistics were recorded from each simulation and these summary statistics served as the inputs for all methods being compared. Reference genotype matrices for each population were generated under the same LD structure and they had the same sample size as the genotype matrices used in generating the outcome. This is to minimize any impact due to poorly estimated LD on model performance. The baseline scenario was extended by varying parameters, including the ratio of sample sizes between each population, levels of LD, the number of causal SNPs, the total number of SNPs in a region, and corresponding effect sizes.

**Set up of Simulation Study on Real LD**

To better capture realistic LD patterns, we performed simulations based on real correlation within three ancestry groups (Europeans, African Americans, and East Asians) from the ELLIPSE OncoArray Consortium^8^. The available sample sizes for these three ancestry groups are 93,749 men of European ancestry, 9,531 men of African ancestry, and 2,075 men of Asian ancestry. We simulated 120 SNPs within a 1334 kb region from chromosome 2 using a multivariate normal model with an estimated correlation structure from individual-level genotypes. A continuous outcome was generated following the same procedure as in artificial LD simulation study and summary statistics were recorded after outcome was generated. The heatmap of this region for each ancestry groups is shown in Figure S2. In each simulation, we randomly chose one SNP out of a selected LD block to be the causal SNP with effect size being 0.04, resulting in an empirical average -log10(*P*-value) of the most significance variant of 7.75 (*P*-value $\approx1.8\times{10}^{-8}$) averaged across 500 simulations.

**Supplemental References**

1. Liang, F., Paulo, R., Molina, G., Clyde, M.A., and Berger, J.O. (2008). Mixtures of <i>g</i> Priors for Bayesian Variable Selection. Journal of the American Statistical Association *103*, 410-423. 10.1198/016214507000001337.

2. Fernández, C., Ley, E., and Steel, M.F.J. (2001). Benchmark priors for Bayesian model averaging. Journal of Econometrics *100*, 381-427. 10.1016/s0304-4076(00)00076-2.

3. Yang, J., Ferreira, T., Morris, A.P., Medland, S.E., Madden, P.A.F., Heath, A.C., Martin, N.G., Montgomery, G.W., Weedon, M.N., Loos, R.J., et al. (2012). Conditional and joint multiple-SNP analysis of GWAS summary statistics identifies additional variants influencing complex traits. Nature Genetics *44*, 369-375. 10.1038/ng.2213.

4. Newcombe, P.J., Conti, D.V., and Richardson, S. (2016). JAM: A Scalable Bayesian Framework for Joint Analysis of Marginal SNP Effects. Genetic Epidemiology *40*, 188-201. 10.1002/gepi.21953.

5. Wang, G., Sarkar, A., Carbonetto, P., and Stephens, M. (2020). A simple new approach to variable selection in regression, with application to genetic fine mapping. Journal of the Royal Statistical Society Series B-Statistical Methodology *82*, 1273-1300. 10.1111/rssb.12388.

6. Zou, Y., Carbonetto, P., Wang, G., Stephens, M.V.O.P., and Stephens, V.O.P.M. (2022). Fine-mapping from summary data with the “Sum of Single Effects” model. bioRxiv. <https://doi.org/10.1101/2021.11.03.467167>.

7. Hormozdiari, F., Kostem, E., Kang, E.Y., Pasaniuc, B., and Eskin, E. (2014). Identifying Causal Variants at Loci with Multiple Signals of Association. Genetics *198*, 497-508. 10.1534/genetics.114.167908.

8. Conti, D.V., Darst, B.F., Moss, L.C., Saunders, E.J., Sheng, X., Chou, A., Schumacher, F.R., Olama, A.A.A., Benlloch, S., Dadaev, T., et al. (2021). Trans-ancestry genome-wide association meta-analysis of prostate cancer identifies new susceptibility loci and informs genetic risk prediction. Nature Genetics *53*, 65-75. 10.1038/s41588-020-00748-0.
