# Supplementary material for: Hierarchical joint analysis of marginal summary statistics—Part I: Multipopulation fine mapping and credible set construction
Source: Genet Epidemiol. Author manuscript; Available in PMC 2025 Apr 9. (PMC11980956; doi:10.1002/gepi.22562)
Supplement: Supp Figures Tables [file NIHMS2066909-supplement-Supp_Figures_Tables.docx]

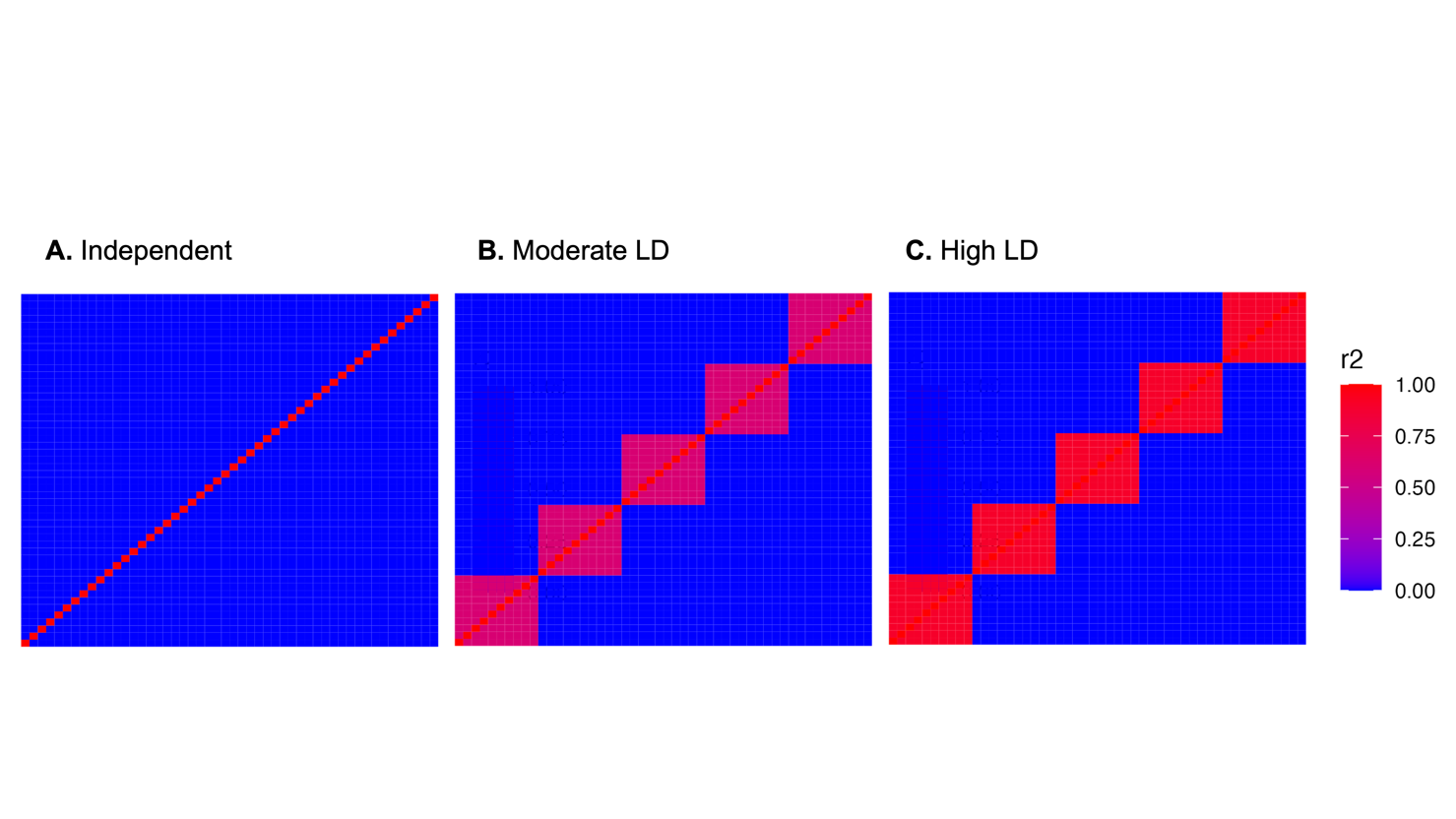


Figure S 1 LD structure in simulation studies

(A) Independent: all SNPs are independent with each other; (B) Moderate LD: 5 blocks of 10 SNPs each where pairwise r^2^ is uniformly set to 0.6; (C) High LD: 5 blocks of 10 SNPs each where pairwise r^2^ is uniformly set to 0.9.


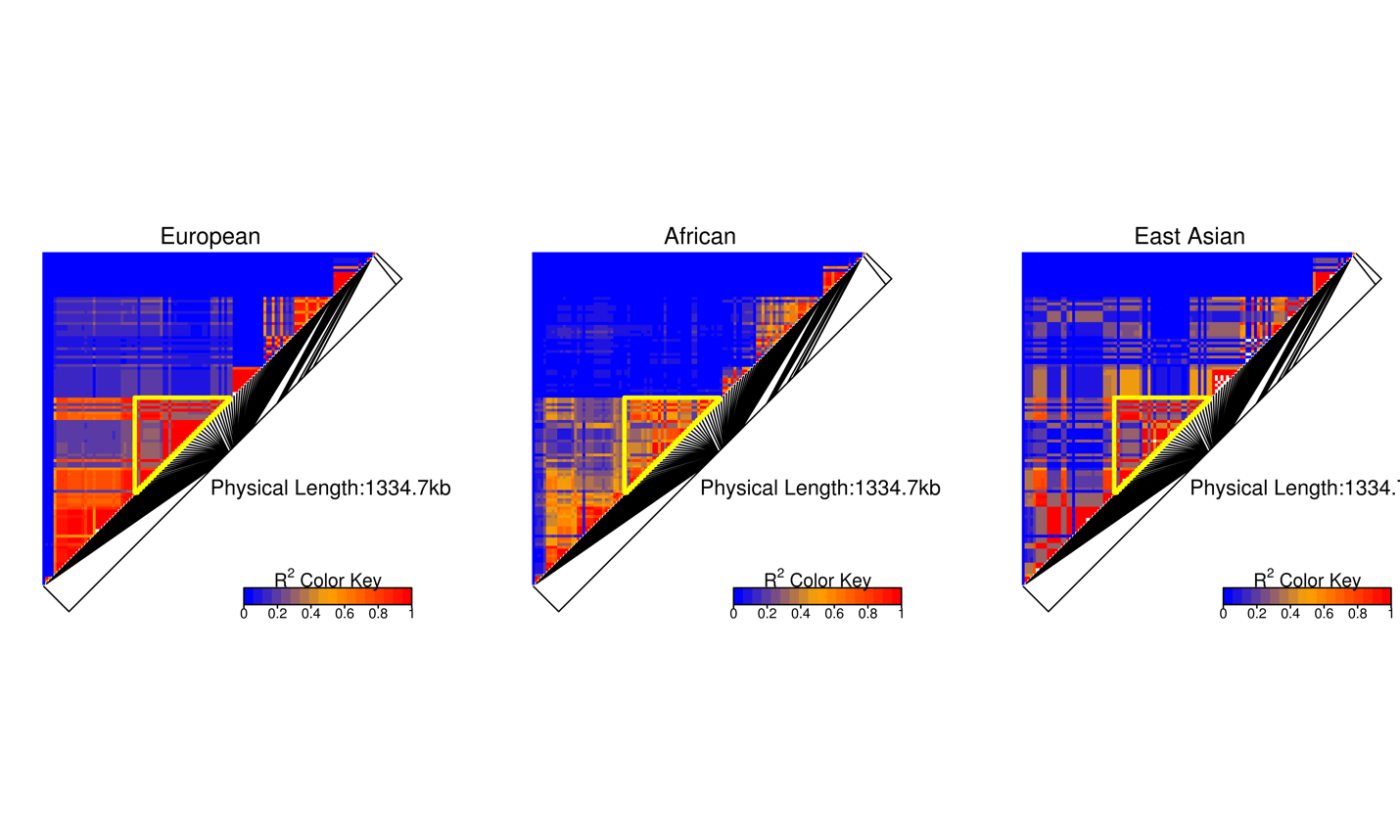


Figure S 2 LD structure of European, African and East Asian populations in chromosome 2 region from position 168212955 to 169812955.

Candidate causal SNP was drawn randomly from the block highlighted in yellow and satisfied meta P-value < 1e-9.


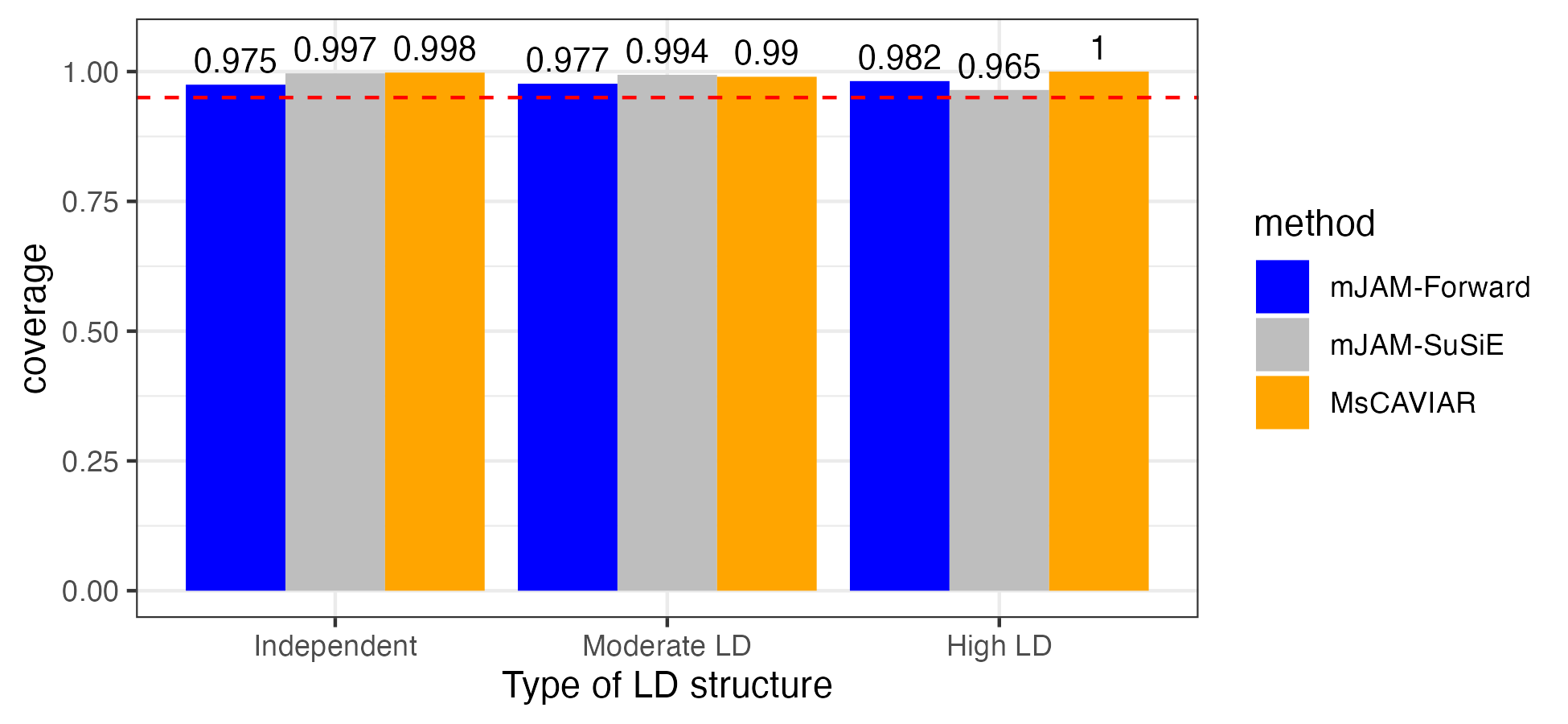


Figure S 3 Empirical coverage for mJAM-Forward, mJAM-SuSiE and MsCAVIAR 95% credible sets under baseline scenario with independent LD structure.

Baseline scenario: 50 SNPs in total, 1 causal SNP with an effect size of 0.03, 3 studies per ancestry group, balanced sample size across populations and independent LD structure. Empirical coverage is defined as the observed proportion of 95% credible sets that included at least one true causal SNP. The requested coverage level of 95% is indicated in red dashed lines.


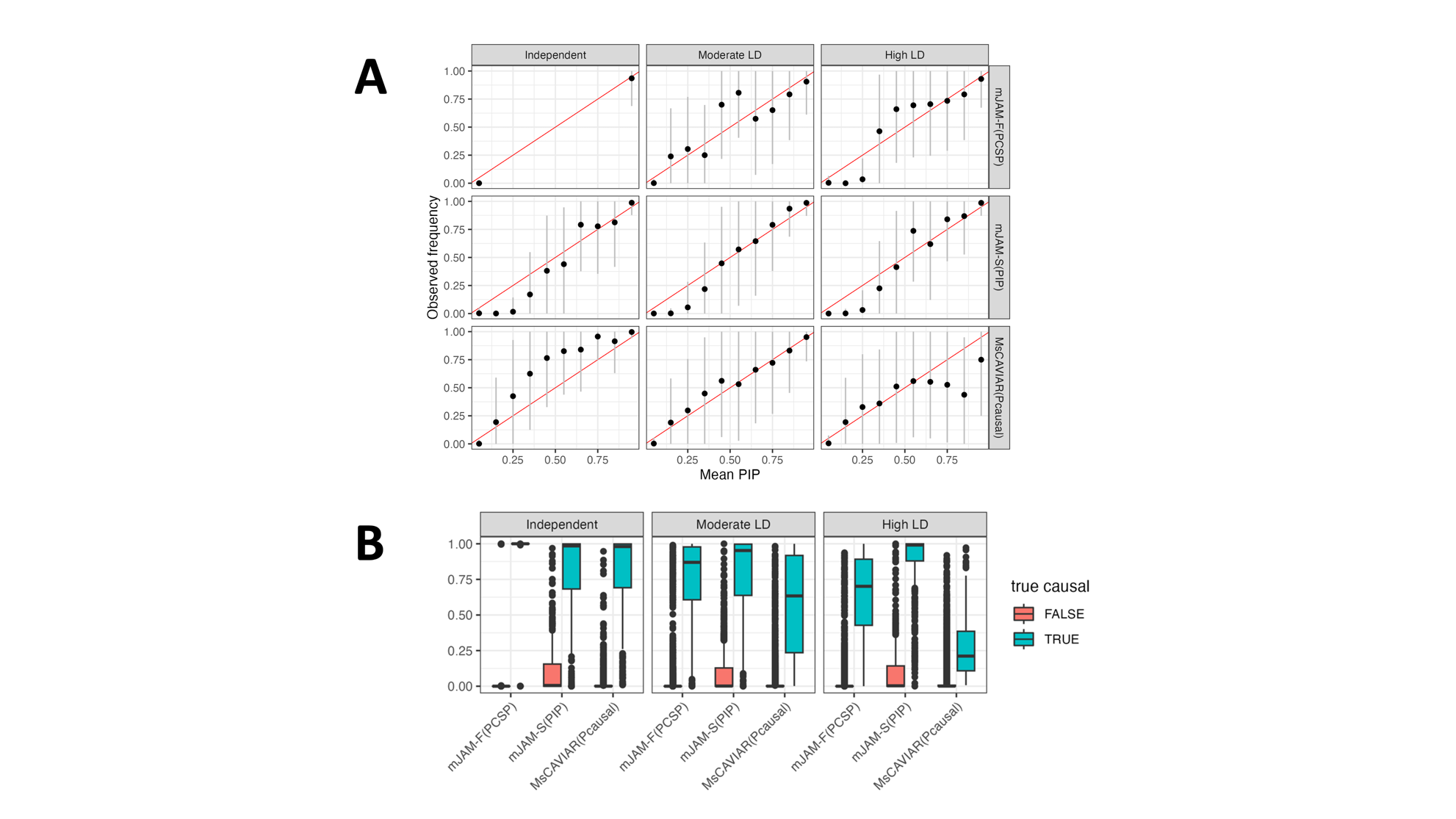


Figure S 4 Assessment of SNP-level probability for mJAM-Forward, mJAM-SuSiE and MsCAVIAR under baseline scenarios.

Baseline scenario: 50 SNPs in total, 1 causal SNP with an effect size of 0.03, 3 studies per ancestry group, and balanced sample size across populations. For independent, moderate, and high LD structure, the pairwise r^2^ between SNPs within each LD block is set to be 0, 0.6^2^, and 0.9^2^ respectively for all populations. mJAM-Forward provides only index-SNP-associated credible sets (i.e. the “PCSP” introduced in the manuscript), and this is different than variant-level PIP in mJAM-SuSiE and MsCAVIAR. For fair comparison on SNP-level probability, we restrict the analysis to simulation scenarios with only 1 causal SNP where mJAM-Forward mostly produced only 1 credible set. (A) Calibration of SNP-level probability. The plot shows the average probability for each bin (10 bins in total) against the proportion of true causal SNPs in that bin. Error lines show the mean PIP ± 1 standard deviation. (B) Distribution of SNP-level probability among true causals and non-causals in 500 simulations.


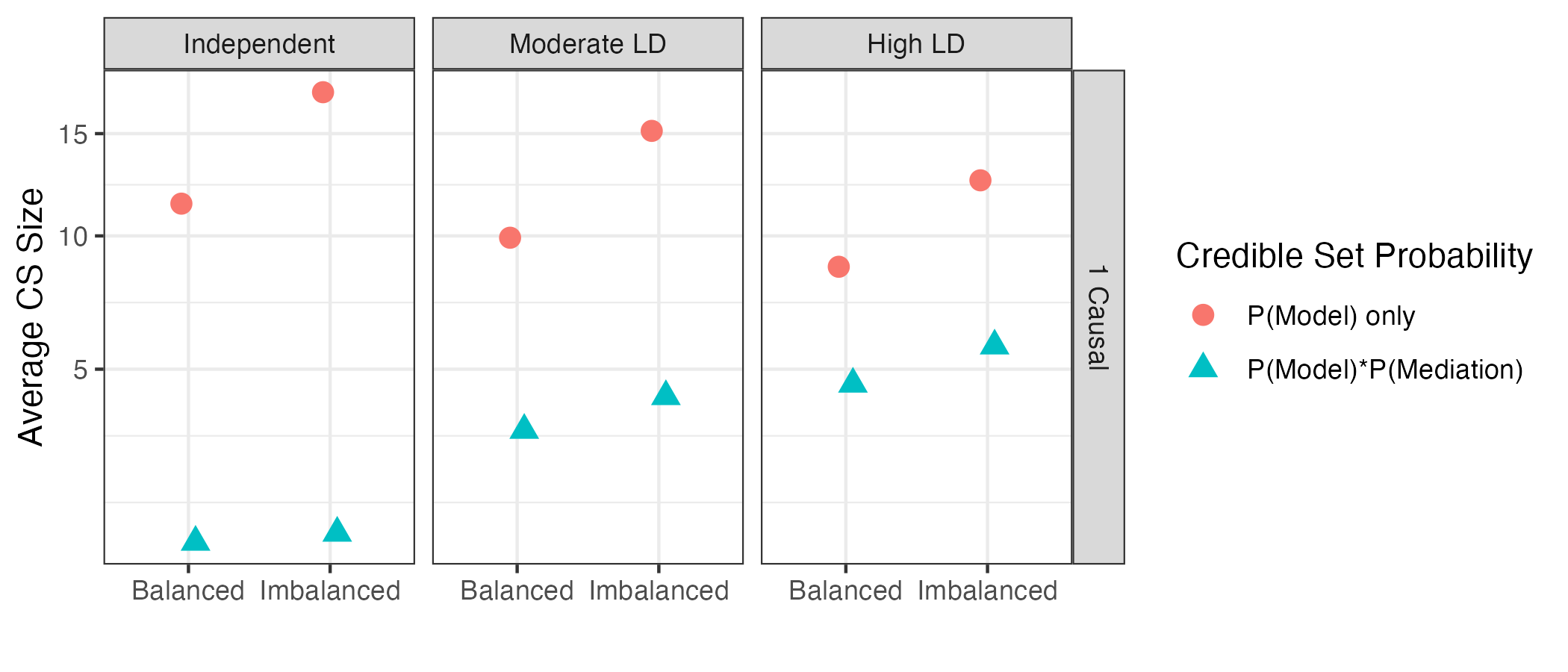


**Figure S 5 Comparison of credible set sizes between two definitions of credible set probability given that the true variant is selected.**

Credible set probability is defined as either using the product of model probability and mediation probability (as in mJAM-Forward) or using only the model probability. To focus on the difference between the two ways of credible set construction, we assume the true variant is selected and credible set is built for the true causal variant.


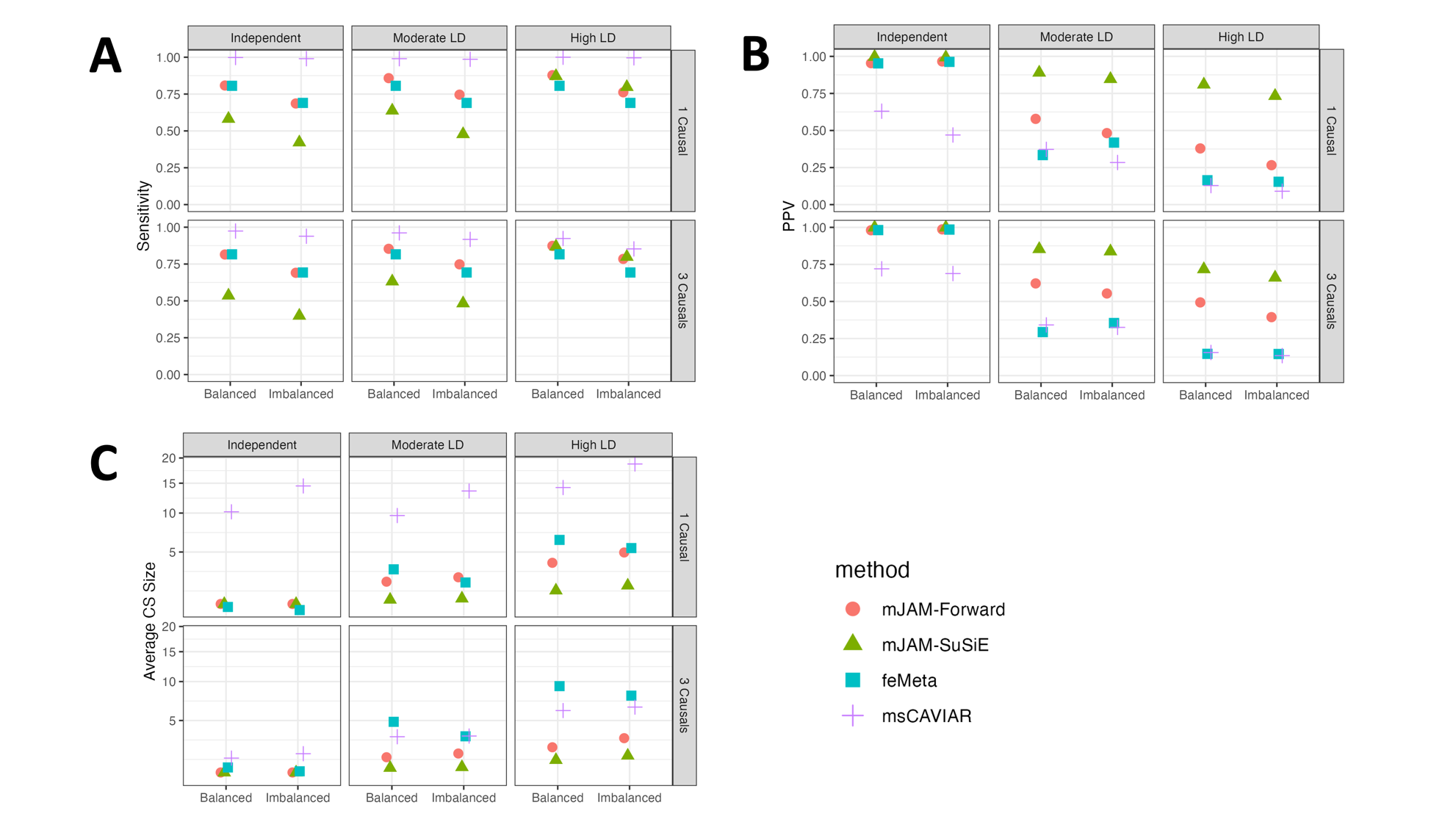


Figure S 6 Credible set performance in simulation studies with artificial LD structure.

For balanced sample size, all 3 populations have a total sample size of 15,000. For unbalanced sample size, the first population has a total sample size of 11,000 and the other two populations have a total sample size of 2,000. Both sample size scenarios have sample size added up to 45,000. For both sample size scenarios, there is either 1 causal SNP or 3 causal SNPs in separate LD blocks, each with an effect size of 0.03; the number of studies per population is set to be 3. (A) Sensitivity, the proportion of true causal SNPs being selected in a credible set, averaged over 500 simulations. (B) PPV, the proportion of true causal SNPs over the total number of selected credible set SNPs, averaged over 500 simulations. (C) Average CS size, the number of SNPs in each 95% credible sets, averaged over 500 simulations.


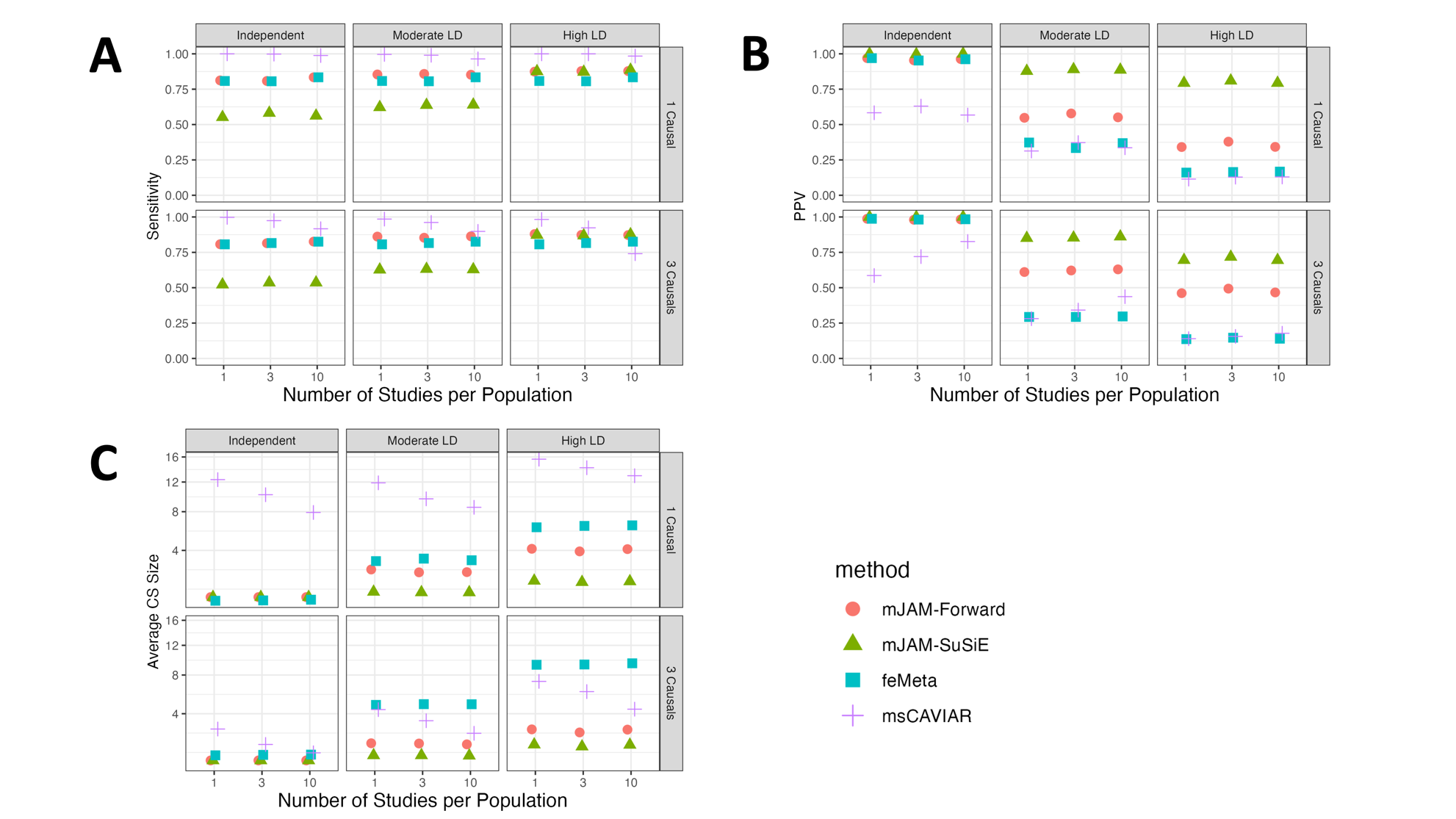


**Figure S 7 Credible set performance in simulation studies with various number of studies from each ancestry group.**

There are 3 ancestry groups in total, each with 1 or 3 or 10 studies. The total sample size for each ancestry group is fixed at 15,000 and sample size is the same across individual studies within each ancestry group. There is either 1 causal SNP or 3 causal SNPs in separate LD blocks, each with an effect size of 0.03. (A) Sensitivity, the proportion of true causal SNPs being selected in a credible set, averaged over 500 simulations. (B) PPV, the proportion of true causal SNPs over the total number of selected credible set SNPs, averaged over 500 simulations. (C) Average CS size, the number of SNPs in each 95% credible sets, averaged over 500 simulations.


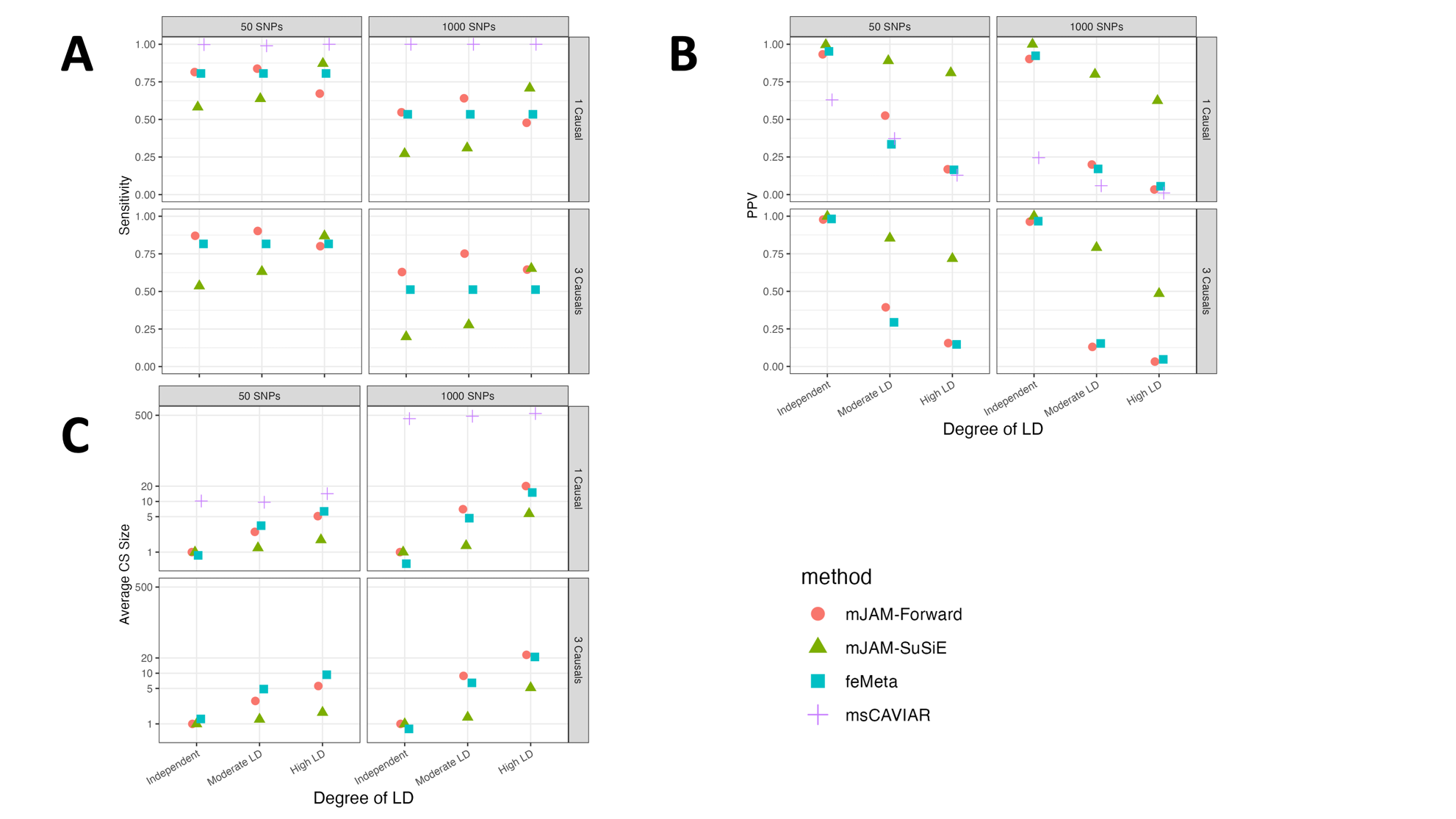


Figure S 8 Credible set performance in simulation studies in large regions with 1000 SNPs.

The left panels show simulation results under regions of 50 SNPs in total; the right panels show results under regions of 1000 SNPs in total. A 50-SNP region is split into 5 LD blocks, each with 10 SNPs. A 1000-SNP region is split into 10 LD blocks, each with 100 SNPs. The pairwise r^2^ within each LD block varies from 0 (independent), 0.6^2^ (moderate) to 0.9^2^ (high). For all scenarios, there is either 1 causal SNP or 3 causal SNPs in separate LD blocks, each with an effect size of 0.03; sample size for each population is fixed at 15,000; the number of studies per population is set to be 3. For scenarios with 3 causal variants, MsCAVIAR took more than 10 hours to finish on the computing node using one Intel Xeon Processor E5-2640 v4 CPU and 40GB memory, and thus we did not include MsCAVIAR’s results under these scenarios. (A) Sensitivity, the proportion of true causal SNPs being selected in a credible set, averaged over 500 simulations. (B) PPV, the proportion of true causal SNPs over the total number of selected credible set SNPs, averaged over 500 simulations. (C) Average CS size, the number of SNPs in each 95% credible sets, averaged over 500 simulations.


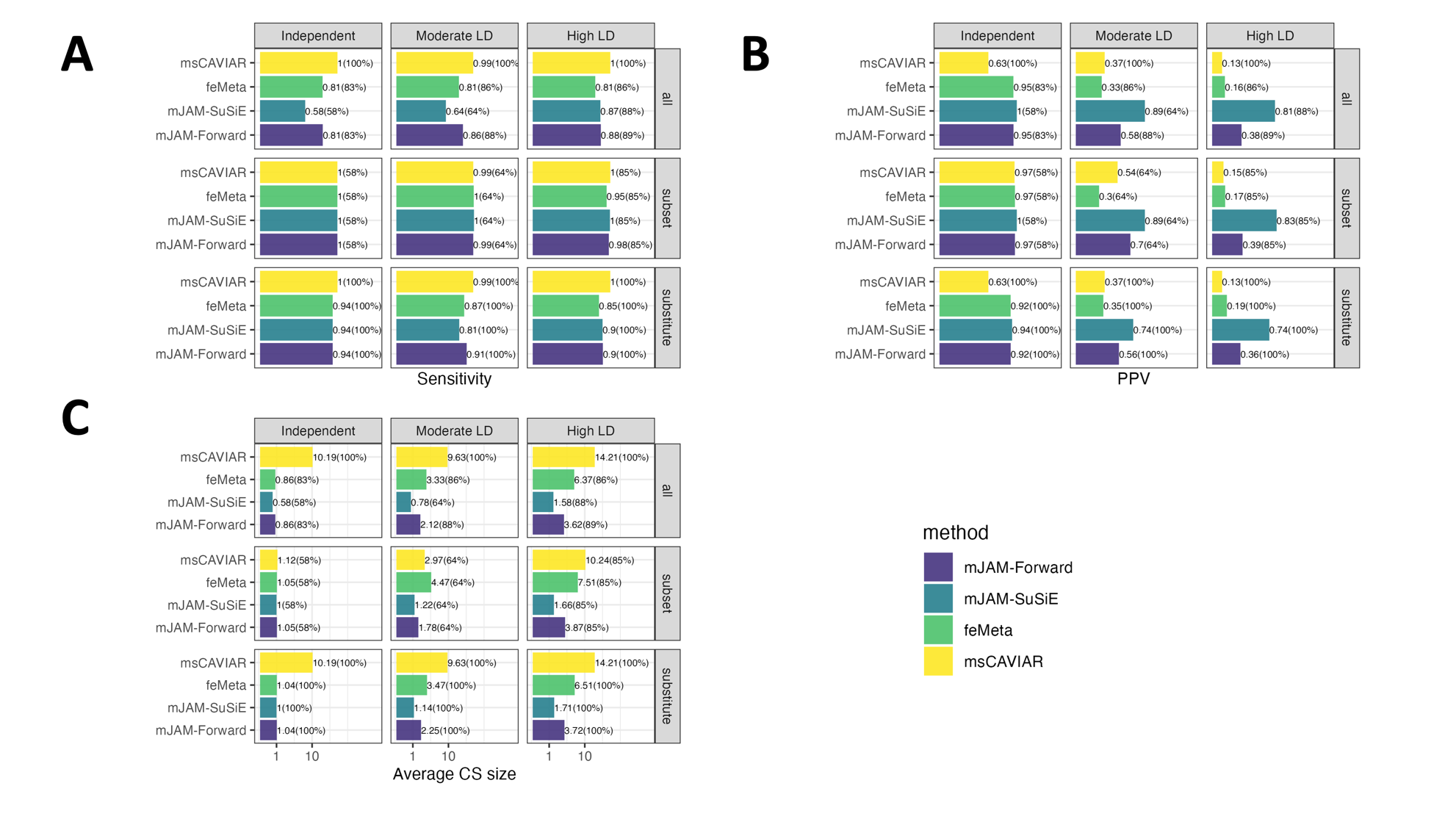


**Figure S 9 Credible set performance when analysis is restricted to simulations when all methods return at least one credible/ set.**

There are 3 ancestry groups in total, each with 3 studies. The total sample size for each ancestry group is fixed at 15,000 and sample size is the same across individual studies within each ancestry group. There is 1 causal SNP with an effect size of 0.03. The first row shows the results based on all 500 simulations under each scenario. In brackets shows the percentage of 500 simulations that there is at least one credible set returned by the method. The second row shows the results based on the subset of 500 simulations that all methods return at least one credible set. The third row shows the results of all 500 simulations where the leading SNP (SNP with the smallest meta P-value) is substituted as the sole credible set if the method returns nothing. (A) Sensitivity, the proportion of true causal SNPs being selected in a credible set, averaged over 500 simulations. (B) PPV, the proportion of true causal SNPs over the total number of selected credible set SNPs, averaged over 500 simulations. (C) Average CS size, the number of SNPs in each 95% credible sets, averaged over 500 simulations.


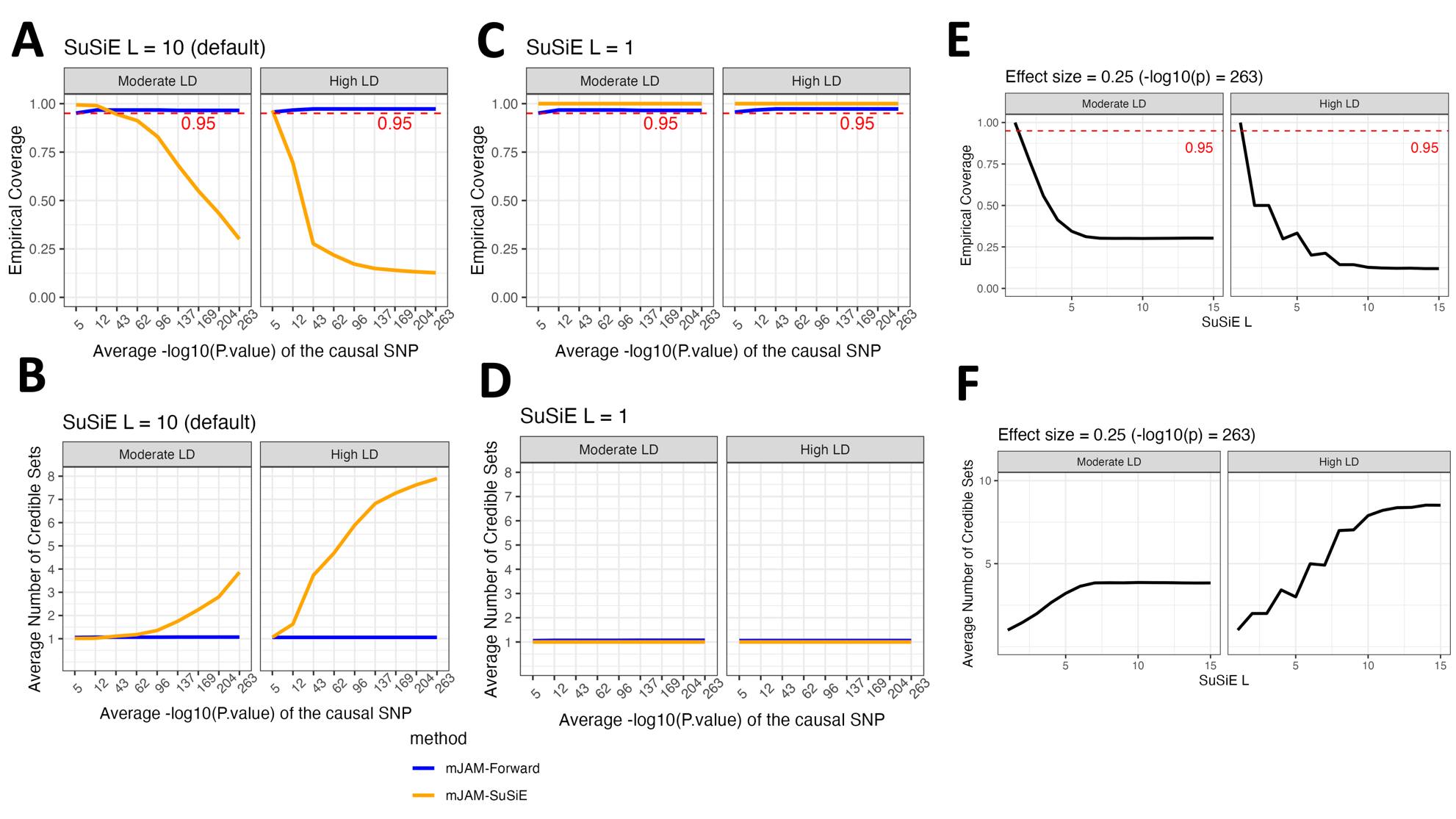


Figure S 10 Extended credible set behaviour of mJAM-SuSiE as causal SNP significance increases.

Simulations were conducted under the baseline scenario setting (1 causal SNP out of 50 SNPs in total which are divided into 5 LD blocks) with varying effect sizes. As a result of increasing effect sizes, the average empirical -log10(P-value) of the causal SNP ranged from 5 to 263, covering most situations seen in practice. Red dashed line indicates requested coverage which is set to be 0.95 for both methods. (A) Empirical credible set coverage when the maximum number of non-zero effects (L) in SuSiE is set to L = 10 (default value in SuSiE), same as Figure 4A; (B) Average number of credible sets selected among 500 simulations when setting L = 10, same as Figure 4B; (C) Empirical credible set coverage when setting L = 1; (D) Average number of credible sets selected when setting L = 1; (E) Empirical credible set coverage when L ranges from 1 to 15 while effect size is kept at 0.25 (largest effect size shown in A-D, equivalent to -log10(P) = 263); (F) Average number of credible sets when L ranges from 1 to 15 while effect size is kept at 0.25 (largest effect size shown in A-D, equivalent to -log10(P) = 263).


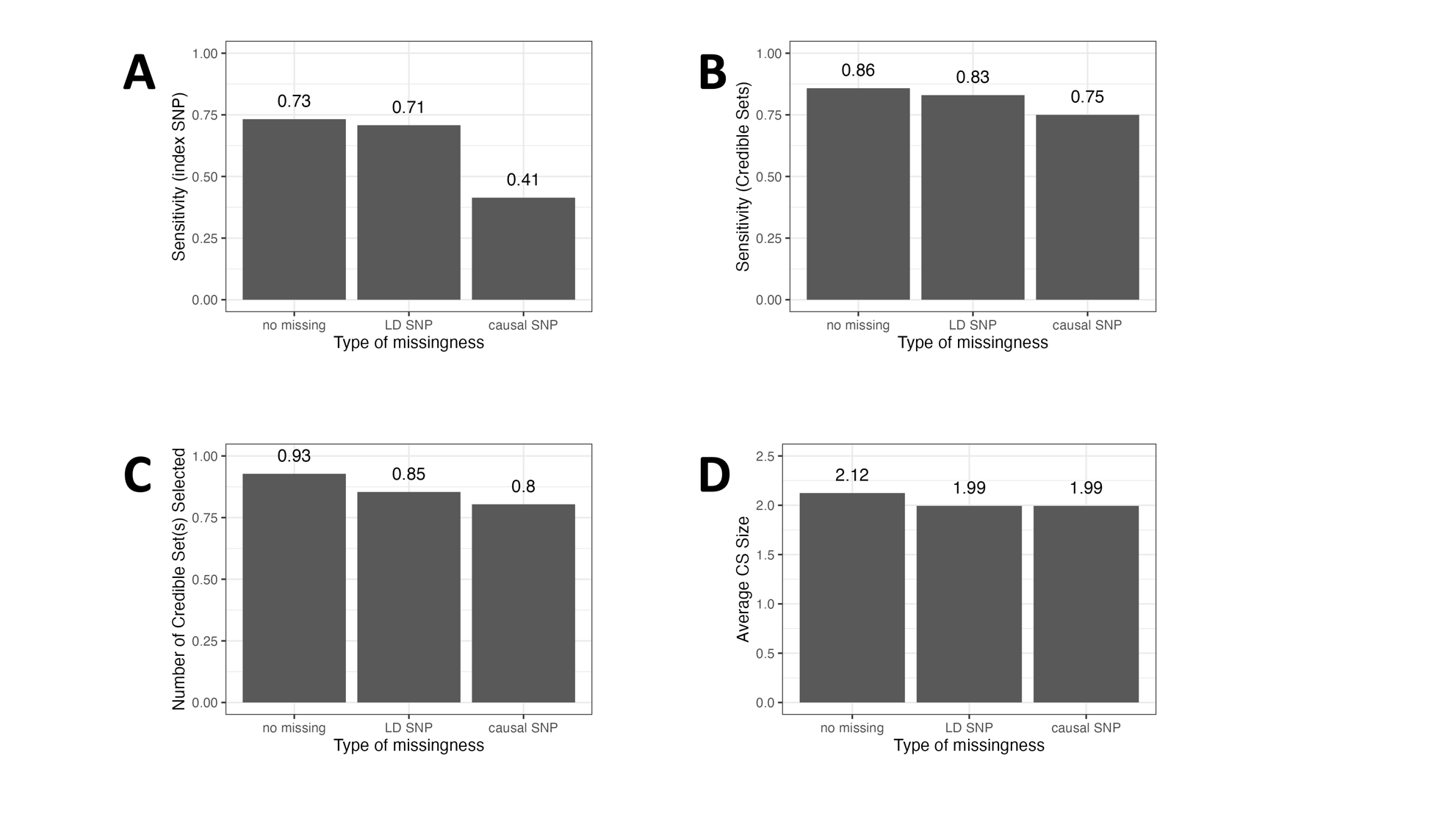


**Figure S 11 Performance of mJAM-Forward in simulation studies with various missingness under baseline scenario.**

Baseline scenario: 1 causal SNP out of 50 SNPs in total; 3 ancestry groups and 3 studies per ancestry group; sample size = 5,000 per study; pairwise r2 between SNPs within each LD block is 0.60. There are 3 types of missingness simulated: (1) no missing: all SNPs are available in each study; (2) LD SNP missing: one SNP that is in the same LD block with the causal SNP is missing in one study. Note that the other two studies of the same ancestry group have this SNP available. (3) causal SNP: the causal SNP is missing in one study but that the other two studies of the same ancestry group have the causal SNP available. (A) Index SNP sensitivity, the proportion of true causal SNPs being selected as an index SNP, averaged over 500 simulations. (B) Credible set sensitivity, the proportion of true causal SNPs being selected in a credible set, averaged over 500 simulations. (C) Number of 95% credible set(s) selected, averaged over 500 simulations. (D) Average CS size, the number of SNPs in each 95% credible sets, averaged over 500 simulations.


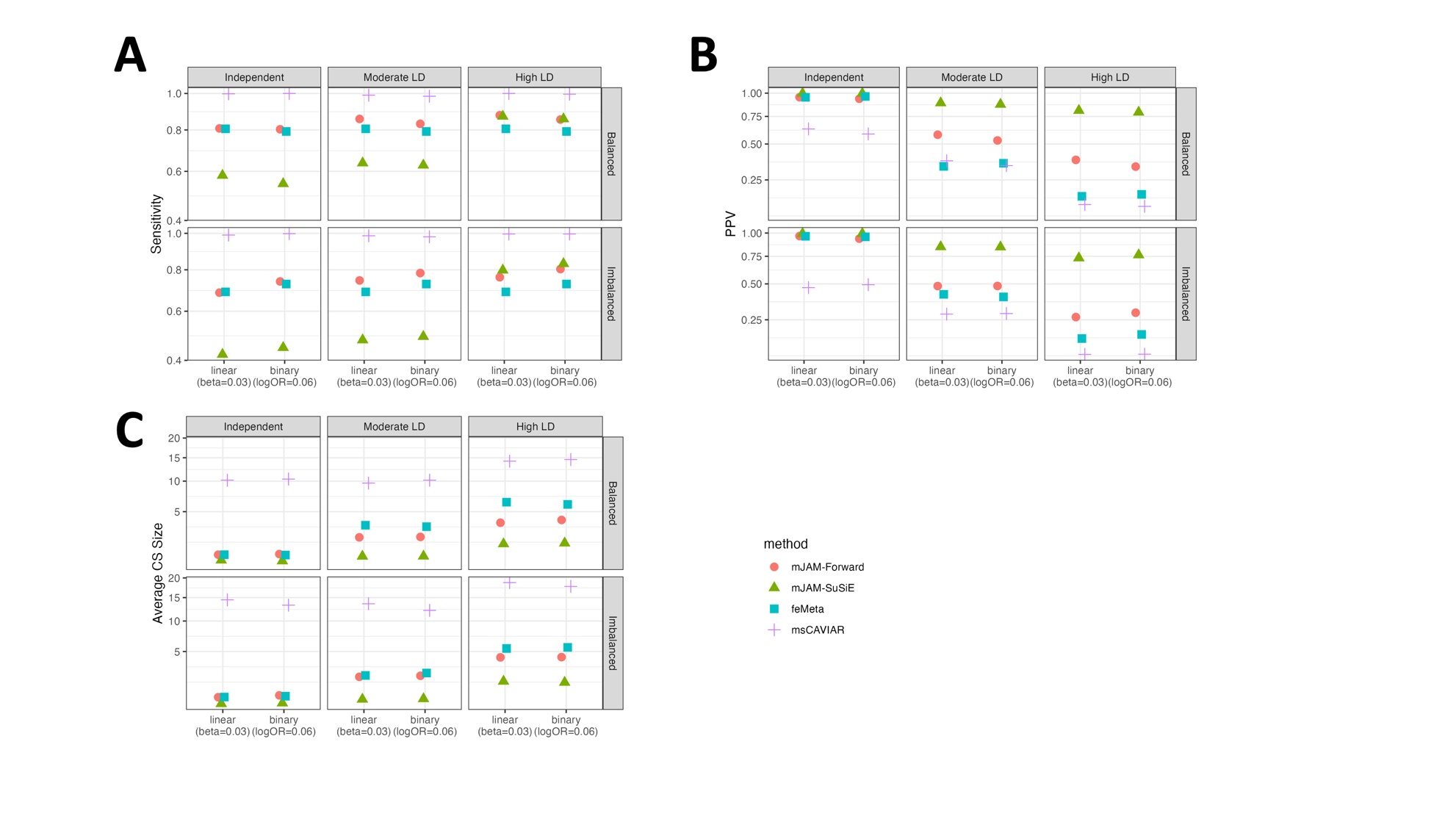


Figure S 12 Credible set performance in simulation studies with continuous outcomes and binary outcomes.

For linear-outcome simulations, an effect size of 0.03 is used; for binary-outcome simulations, a log odds ratio of 0.06 is used so that the empirical power of identifying the true causal SNP under both scenarios is kept at around 80%. For balanced sample size, all 3 populations have a total sample size of 15,000. For unbalanced sample size, the first population has a total sample size of 11,000 and the other two populations have a total sample size of 2,000. Both sample size scenarios have sample size added up to 45,000. The pairwise r^2^ within each LD block varies from 0 (independent), 0.6^2^ (moderate) to 0.9^2^ (high). (A) Sensitivity, the proportion of true causal SNPs being selected in a credible set, averaged over 500 simulations. (B) PPV, the proportion of true causal SNPs over the total number of selected credible set SNPs, averaged over 500 simulations. (C) Average CS size, the number of SNPs in each 95% credible sets, averaged over 500 simulations.


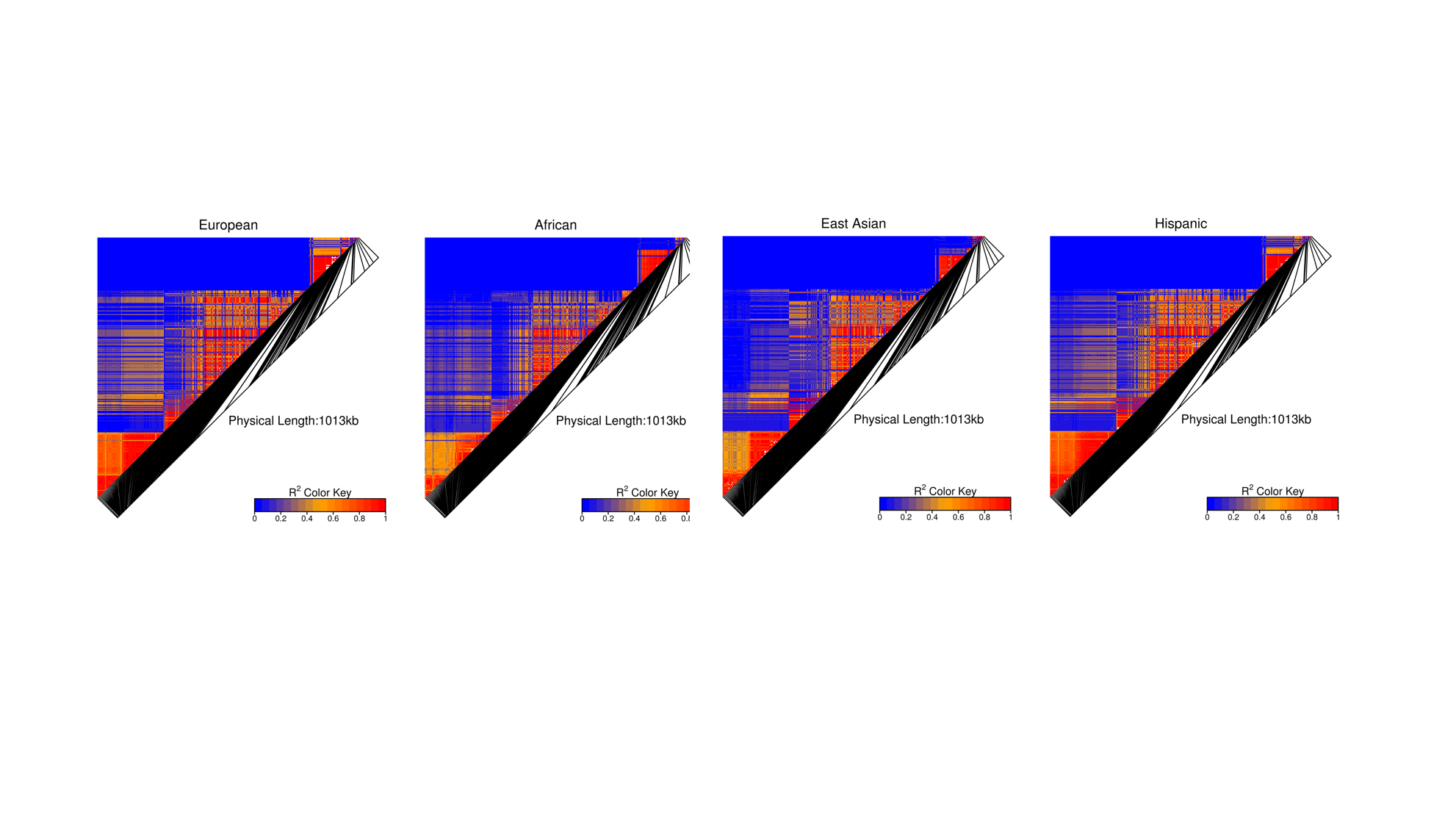


**Figure S 13 Ethnic-specific LD structure for 416 SNPs at chromosome 12 from position 109194870 to 110794870.**

Analysis restricted to SNPs with meta-analyzed P-value < 0.001 and MAF > 2%.


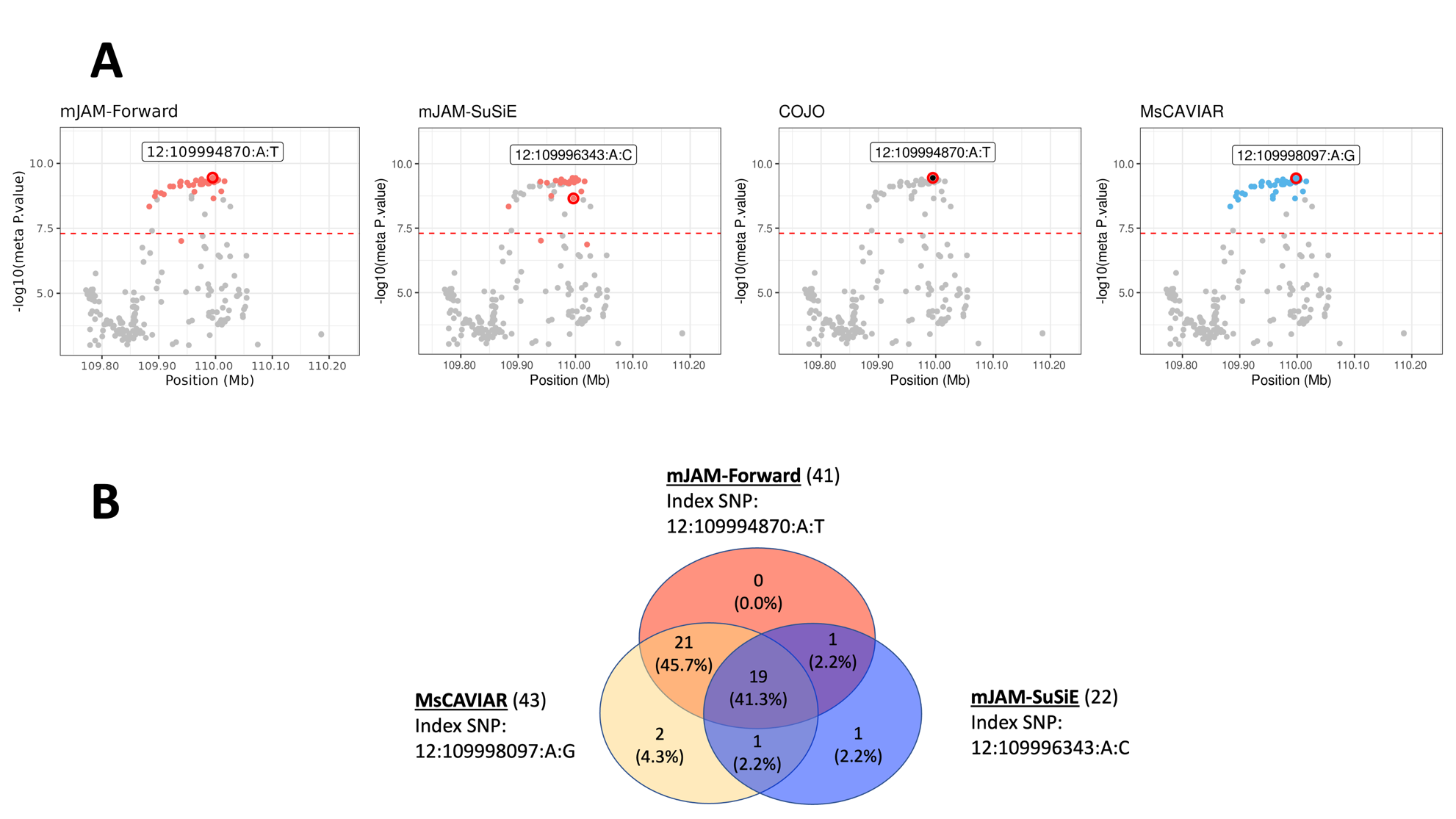


**Figure S 14 Fine-mapping results for chromosome 12 region from position 109194870 to 110794870.**

(A) From left to right is mJAM-Forward 95% credible set, mJAM-SuSiE 95% credible set, index SNP selected by COJO, and MsCAVIAR 95% credible set. Index SNPs are circled in red. SNPs in the same credible sets are highlighted in the same color. Genome-wide significance ( $5\times{10}^{-8}$ ) is shown in red dashed line. Index variants are labelled with GRCh37/hg19 reference assembly co-ordinates. (B) Venn diagram showing the overlap between 95% credible sets from mJAM-Forward, mJAM-SuSiE and MsCAVIAR.


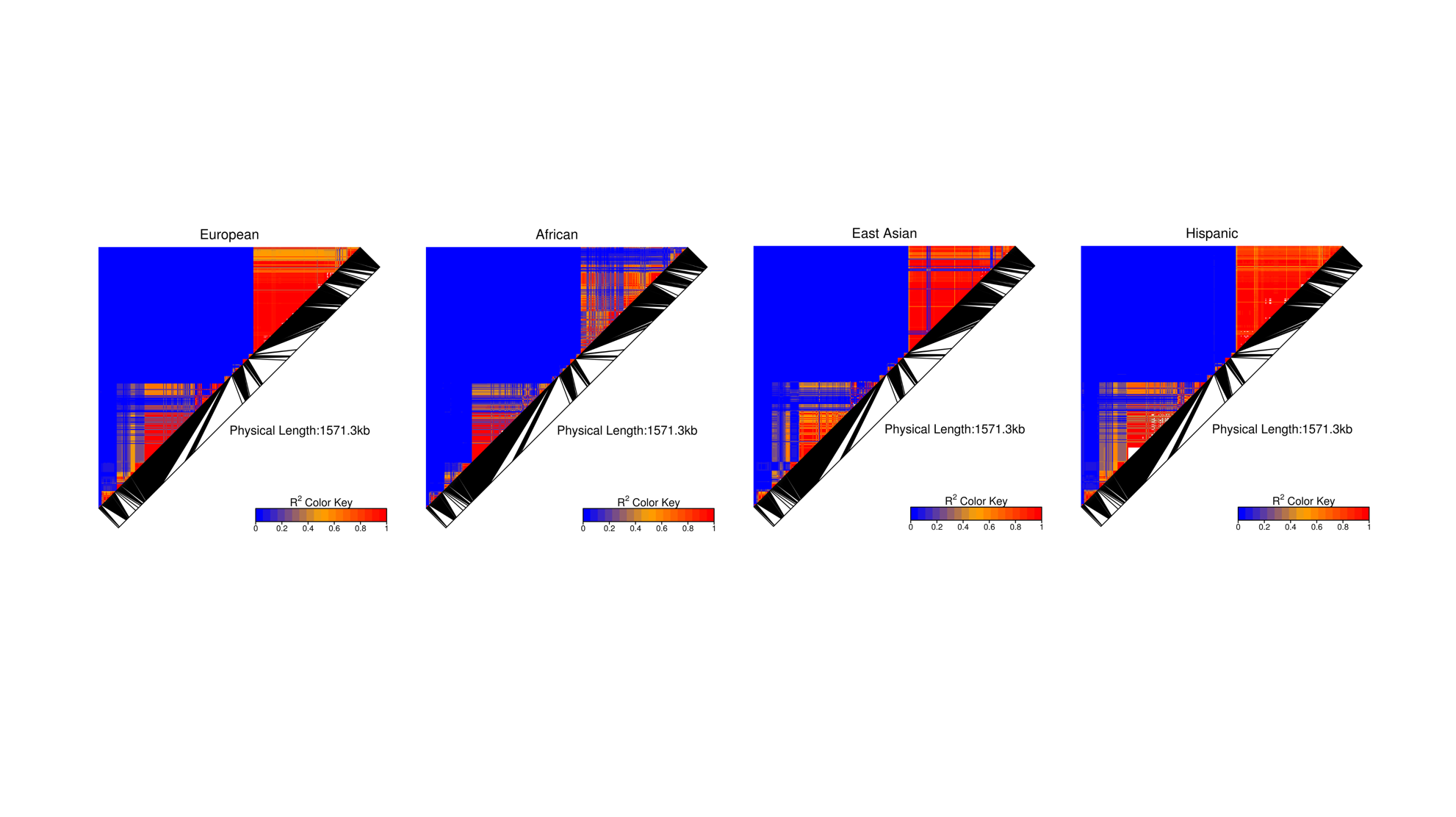


**Figure S 15 Ethnic-specific LD structure for 416 SNPs at chromosome 10 from position 79436999 to 81635998.**

Analysis restricted to SNPs with meta-analyzed P-value < 0.001 and MAF > 2%.


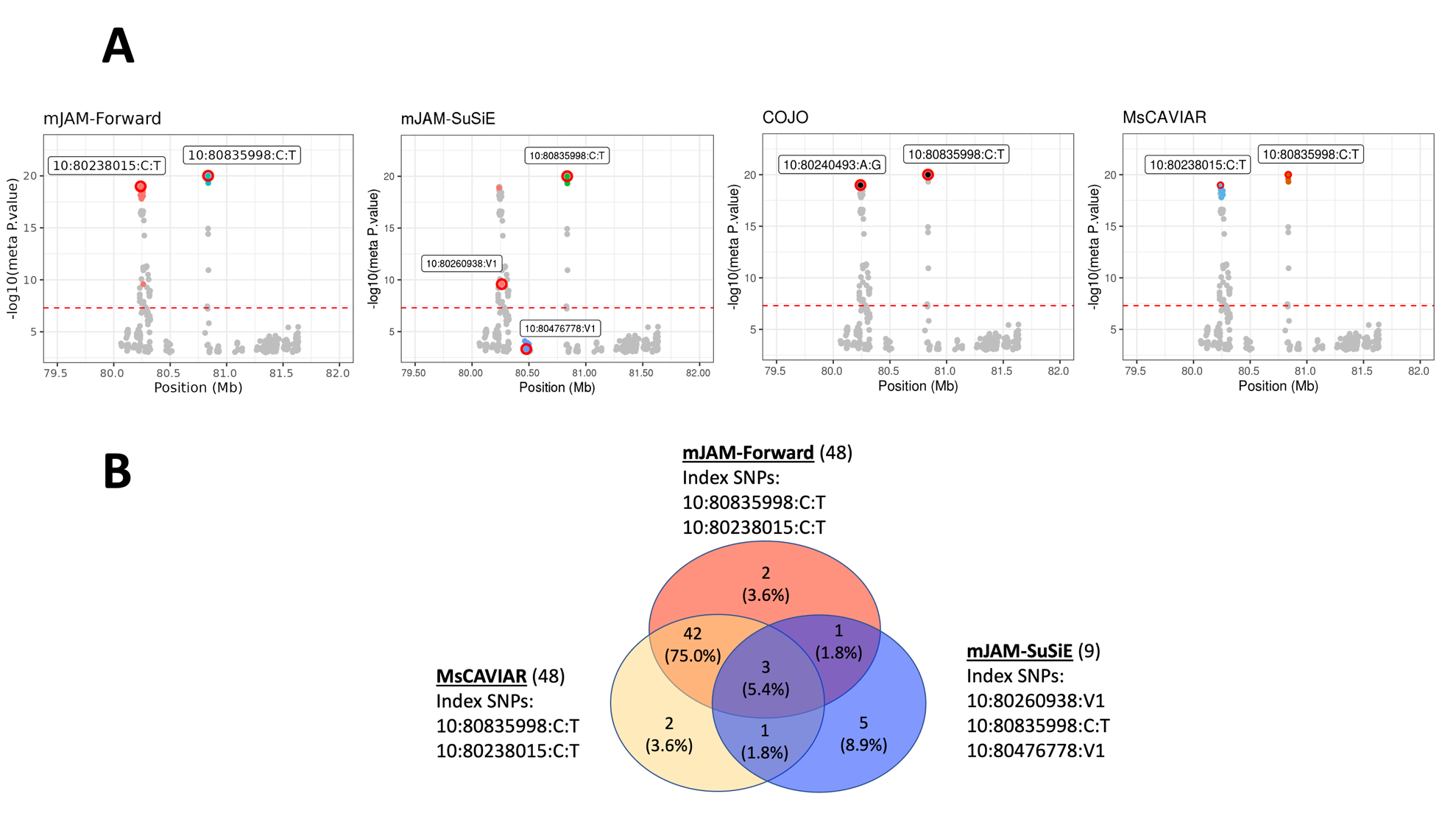


**Figure S 16 Fine-mapping results for chromosome 10 region from position 79436999 to 81635998.**

(A) From left to right is mJAM-Forward 95% credible set, mJAM-SuSiE 95% credible set, index SNP selected by COJO, and MsCAVIAR 95% credible set. Index SNPs are circled in red. SNPs in the same credible sets are highlighted in the same color. Genome-wide significance ( $5\times{10}^{-8}$ ) is shown in red dashed line. Index variants are labelled with GRCh37/hg19 reference assembly co-ordinates. (B) Venn diagram showing the overlap between 95% credible sets from mJAM-Forward, mJAM-SuSiE and MsCAVIAR.


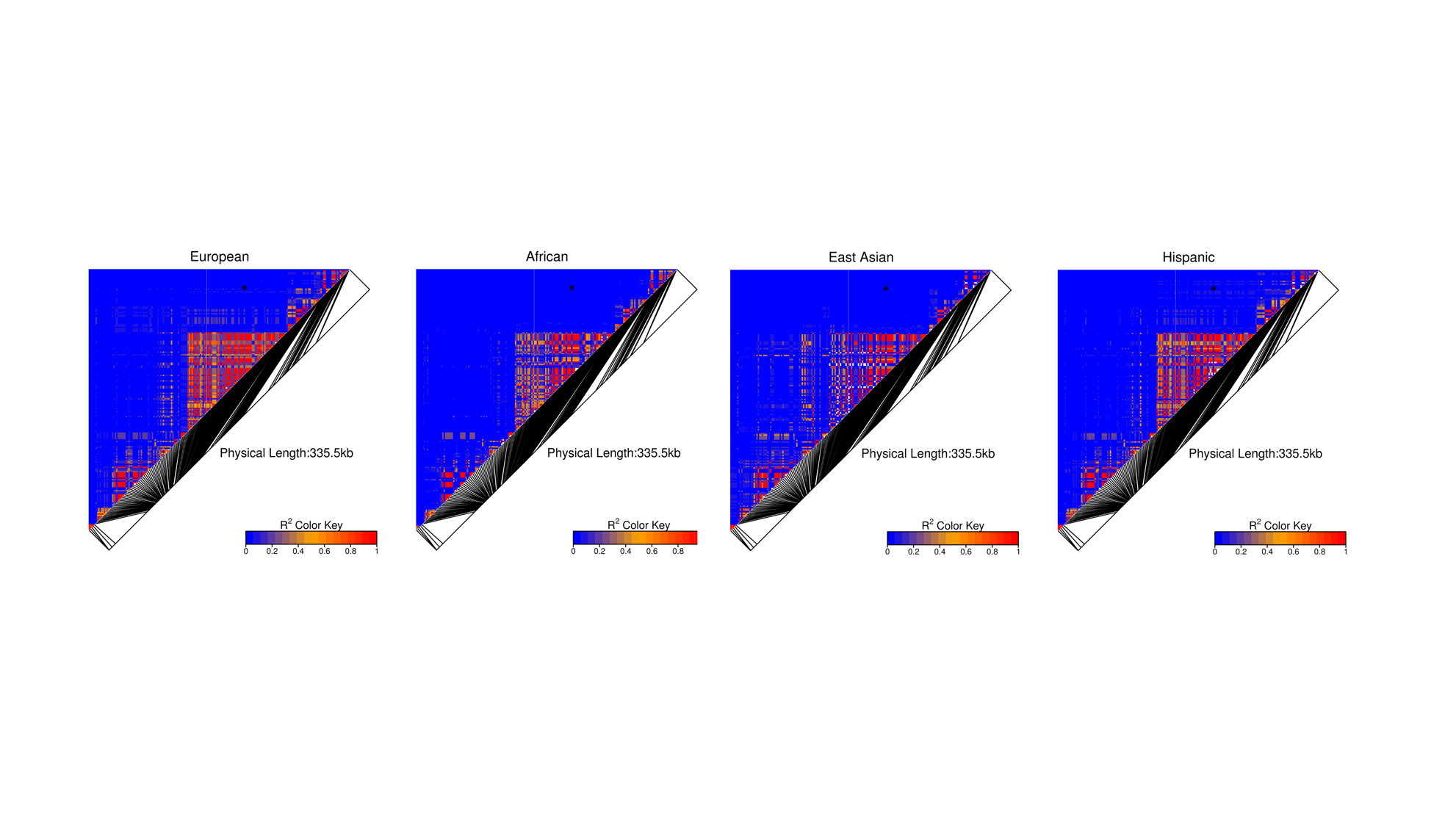


**Figure S 17 Ethnic-specific LD structure for 416 SNPs at chromosome 11 from position 101601661 to 103201661.**

Analysis restricted to SNPs with meta-analyzed P-value < 0.001 and MAF > 2%. The pairwise correlation between 11:102440927:A:G and 11:102401661:C:T is marked in black asterisk.


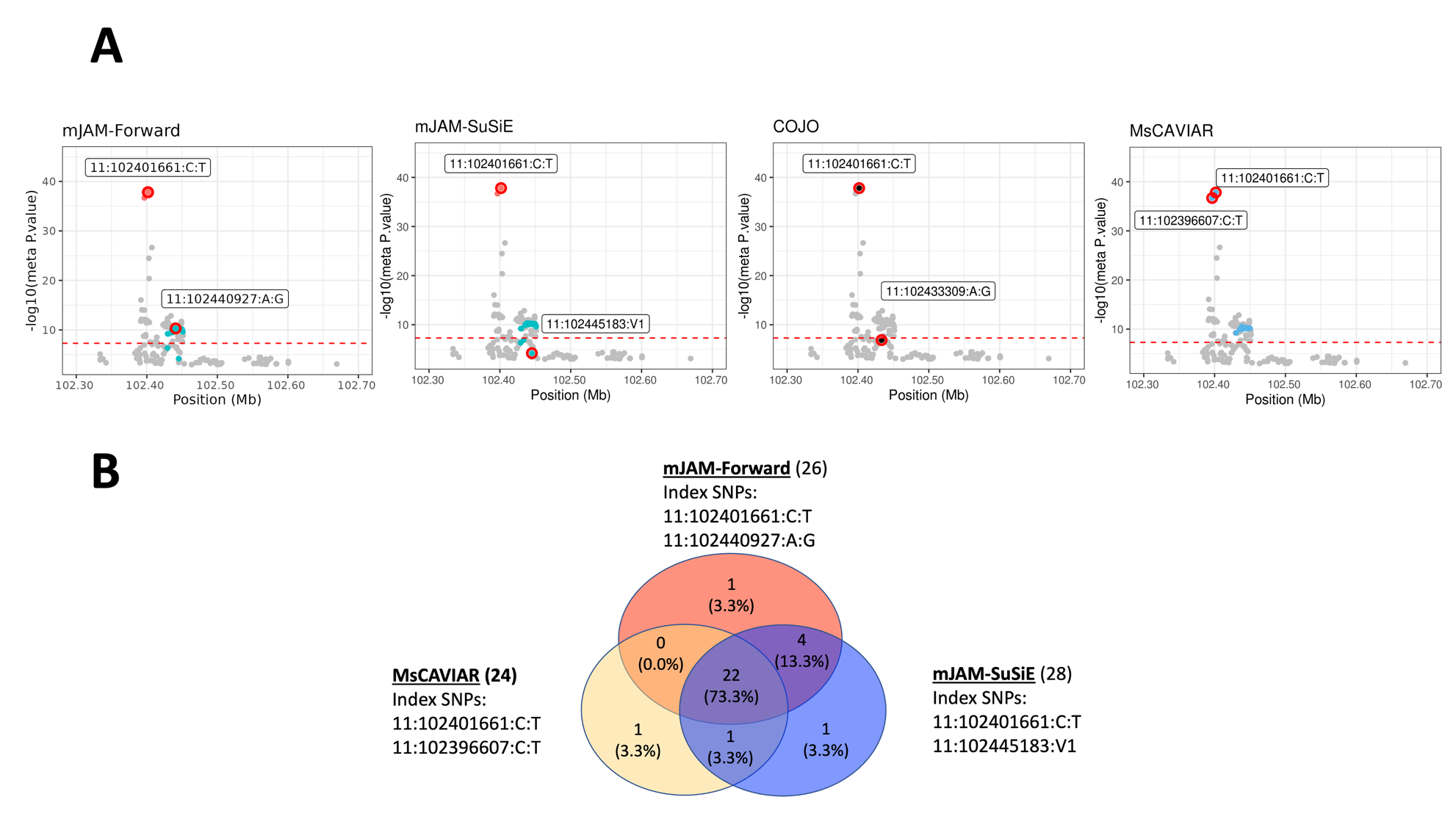


**Figure S 18 Fine-mapping results for chromosome 11 region from position 101601661 to 103201661.**

(A) From left to right is mJAM-Forward 95% credible set, mJAM-SuSiE 95% credible set, index SNP selected by COJO, and MsCAVIAR 95% credible set. Index SNPs are circled in red. SNPs in the same credible sets are highlighted in the same color. Genome-wide significance ( $5\times{10}^{-8}$ ) is shown in red dashed line. Index variants are labelled with GRCh37/hg19 reference assembly co-ordinates. (B) Venn diagram showing the overlap between 95% credible sets from mJAM-Forward, mJAM-SuSiE and MsCAVIAR.


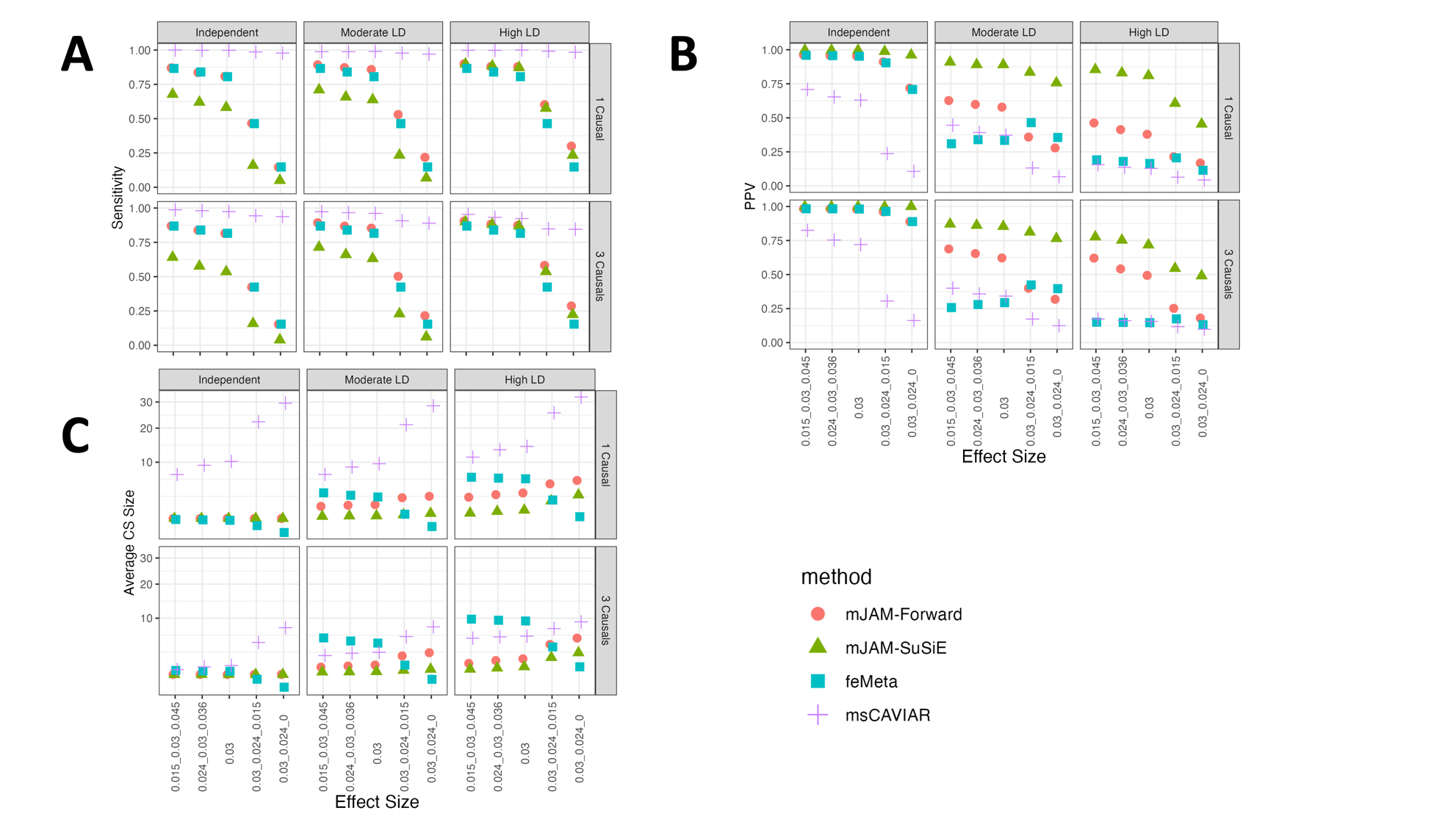


**Figure S 19 Credible set performance when true effect size is heterogeneous across populations.**

There are 3 ancestry groups in total, each with 3 studies. The total sample size for each ancestry group is fixed at 15,000 and sample size is the same across individual studies within each ancestry group. There is either 1 causal SNP or 3 causal SNPs in separate LD blocks. The pairwise r^2^ within each LD block varies from 0 (independent), 0.6^2^ (moderate) to 0.9^2^ (high). The x-axis shows the effect size settings: the left two settings are 20% or 50% variation in population-specific effects but the pooled average is kept at 0.03; the middle setting is the baseline scenario where all populations share the same effect size; the right two settings kept the first population’s effect size at 0.03 but reduced the other two populations’ effect sizes till below 0.03 and even to 0. (A) Sensitivity, the proportion of true causal SNPs being selected in a credible set, averaged over 500 simulations. (B) PPV, the proportion of true causal SNPs over the total number of selected credible set SNPs, averaged over 500 simulations. (C) Average CS size, the number of SNPs in each 95% credible sets, averaged over 500 simulations. Moderate degree of variation (up to 50%) in population-specific effect sizes does not change the credible set performance of all the methods substantially. Under some scenarios where one population have larger effect sizes and more significant findings of the true variant (effect size = {0.015, 0.003, 0.045} and {0.024, 0.03, 0.036}), the overall performance might be slightly better than common effect across populations.

**Table S 1 Minimum, mean and median pairwise r2 of credible set SNPs with corresponding index SNP for the chr 12 region.**

EUR, European; AFR, African American; HISP, Hispanic; ASI, East Asian.

|  | | EUR | AFR | HISP | ASI |
| --- | --- | --- | --- | --- | --- |
| mJAM-Forward | min | 0.62 | 0.35 | 0.74 | 0.33 |
|  | mean | 0.98 | 0.95 | 0.98 | 0.94 |
|  | median | 1.00 | 0.98 | 0.99 | 1.00 |
| mJAM-SuSiE | min | 0.60 | 0.58 | 0.62 | 0.41 |
|  | mean | 0.97 | 0.95 | 0.97 | 0.95 |
|  | median | 1.00 | 0.99 | 1.00 | 1.00 |
| MsCAVIAR | min | 0.62 | 0.35 | 0.74 | 0.33 |
|  | mean | 0.98 | 0.95 | 0.98 | 0.94 |
|  | median | 1.00 | 0.97 | 0.99 | 1.00 |

**Table S 2 Minimum, mean and median pairwise r2 of credible set SNPs with corresponding index SNP for the chr 10 region.**

EUR, European; AFR, African American; HISP, Hispanic; ASI, East Asian.

|  | | EUR | AFR | HISP | ASI |
| --- | --- | --- | --- | --- | --- |
| mJAM-Forward | min | 0.95 | 0.81 | 0.95 | 0.97 |
|  | mean | 0.96 | 0.98 | 0.99 | 1.00 |
|  | median | 0.96 | 0.98 | 0.98 | 1.00 |
| mJAM-SuSiE | min | 0.33 | 0.12 | 0.13 | 0.24 |
|  | mean | 0.79 | 0.69 | 0.73 | 0.72 |
|  | median | 0.97 | 0.88 | 0.97 | 0.99 |
| MsCAVIAR | min | 0.95 | 0.81 | 0.97 | 0.98 |
|  | mean | 0.96 | 0.98 | 0.99 | 1.00 |
|  | median | 0.96 | 0.98 | 0.98 | 1.00 |

**Table S 3 Minimum, mean and median pairwise r2 of credible set SNPs with corresponding index SNP for the chr 11 region.**

EUR, European; AFR, African American; HISP, Hispanic; ASI, East Asian.

|  | | EUR | AFR | HISP | ASI |
| --- | --- | --- | --- | --- | --- |
| mJAM-Forward | min | 0.35 | 0.16 | 0.38 | 0.35 |
|  | mean | 0.96 | 0.91 | 0.96 | 0.95 |
|  | median | 1.00 | 0.99 | 1.00 | 1.00 |
| mJAM-SuSiE | min | 0.22 | 0.07 | 0.22 | 0.13 |
|  | mean | 0.42 | 0.23 | 0.44 | 0.41 |
|  | median | 0.35 | 0.16 | 0.38 | 0.35 |
| MsCAVIAR | min | 1.E-05 | 3.E-03 | 7.E-03 | 9.E-02 |
|  | mean | 6.E-02 | 7.E-02 | 7.E-02 | 2.E-01 |
|  | median | 3.E-05 | 2.E-02 | 9.E-03 | 1.E-01 |

**Table S 4 Runtime (in seconds) of each method under baseline simulation scenario.**

Baseline simulation scenario: 50 SNPs in total consisting of 5 LD blocks (each with 10 SNPs); pairwise r^2^ within a LD block is 0.6^2^. 3 populations with 3 studies in each population; sample size for each population fixed at 15,000. All methods are run with one Intel Xeon Processor E5-2640 v4 processor and maximum memory space of 40GB.

|  | Runtime of each method (sec) | | | | |
| --- | --- | --- | --- | --- | --- |
| Number of Causal SNPs in a region | FE | mJAM_Forward | mJAM_SuSiE | COJO | MsCAVIAR |
| 1 | 0.89 | 0.97 | 0.18 | 0.19 | 0.46 |
| 2 | 0.77 | 1.14 | 0.18 | 0.20 | 9.22 |
| 3 | 0.88 | 1.82 | 0.19 | 0.22 | 150.26 |
